# Challenges and strategies for first-principles simulations of two-dimensional magnetic phenomena

**DOI:** 10.1039/d4nr05503a

**Published:** 2025-07-07

**Authors:** Jaime Garrido Aldea, Dorye L. Esteras, Stephan Roche, Jose H. Garcia

**Affiliations:** a Catalan Institute of Nanoscience and Nanotechnology (ICN2)CSIC and BIST Campus UAB Bellaterra Barcelona 08193 Spain jaime.garrido@icn2.cat; b Universitat Autànoma de Barcelona (UAB) 08193 Barcelona Spain; c Institució Catalana de Recerca i Estudis Avançats (ICREA) Spain

## Abstract

The discovery of intrinsic magnetism in two-dimensional (2D) materials has opened new frontiers in material science and technology. This review offers a detailed guide to modeling 2D magnetic materials using Density Functional Theory (DFT), focusing on both fundamental concepts and practical methodologies. Starting with the principles of magnetism, it examines the unique challenges of 2D systems, including the effects of anisotropy in stabilizing magnetic order, the limitations imposed by the Mermin–Wagner theorem, and the critical role of exchange interactions. The review introduces DFT basics, highlighting approaches to address electron delocalization through methods like DFT+*U* and hybrid functionals, and emphasizes the importance of incorporating van der Waals corrections for layered systems. Strategies for determining ground-state spin configurations for both collinear and non-collinear arrangements, are discussed, alongside advanced techniques like spin-constrained DFT and the Generalized Bloch Theorem for spin-spiral states. Methods for extracting magnetic exchange parameters and estimating critical temperatures from first-principles calculations are comprehensively covered. Practical insights are provided for applying these techniques to explore material databases and identify 2D magnets with promising properties for room-temperature applications. This review serves as a resource for theoretical and computational studies of 2D magnetic materials.

## Abstract 2

This review introduces key concepts and tools for modelling 2D magnets with DFT, covering magnetism basics, challenges like spin order, and methods to extract Hamiltonian parameters and critical temperatures for various anisotropies.

## Graphical abstract

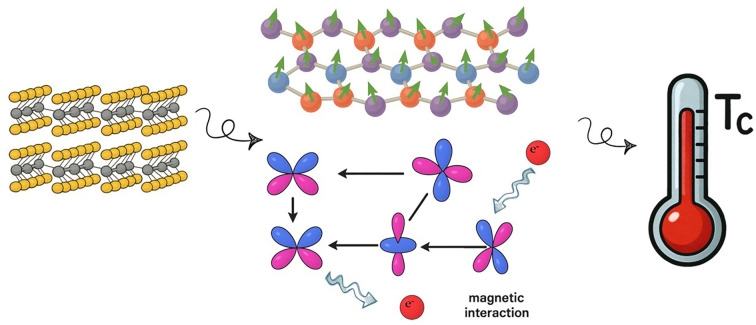

## 1. Introduction

The first experimental measurement of ferromagnetic order in a monolayer was carried out in 2017 for the out of plane ferromagnet CrI_3_,^1^ obtaining a Curie temperature (*T*_c_) of 45 K. Shortly after, ferromagnetism in bilayer Cr_2_Ge_2_Te_6_ was found,^2^ with a *T*_c_ that can be tuned by introducing small out-of-plane magnetic fields. In 2018, ferromagnetic order was found for Fe_3_GeTe_2_ up to 130 K for a single monolayer.^3^

2D magnetic materials have remained so elusive all this time as a consequence of the Mermin–Wagner theorem,^4^ known since 1966. This theorem states that any 2D material with infinite size and with a continuous symmetry cannot present long range magnetic order at nonzero temperature when considering an isotropic Heisenberg model with finite range exchange interactions. Fortunately, this theorem does not contemplate anisotropy, and we know as of today that introducing single ion anisotropy or exchange anisotropy can stabilize magnetic order in 2D at nonzero temperature.^5,6^

These groundbreaking discoveries have positioned 2D magnetic materials as pivotal components for emerging technologies, including spintronics,^7,8^ magnetic sensors,^9^ energy harvesting systems and green energy applications^10,11^ and nonvolatile magnetic memories.^12^ Moreover, their 2D nature introduces unique opportunities, such as stacking and twisting, to tailor properties for specific applications.^13^

For practical device applications, achieving magnetic stability above room temperature remains a crucial challenge. Efforts are underway to identify 2D magnets with high intrinsic *T*_c_.^5,14–19^ While various techniques exist to enhance magnetic stability,^20^ finding materials with intrinsic stability is paramount, as many enhancement methods can complicate fabrication or alter other key properties.

The rapid proliferation of novel 2D materials^21^ and the development of extensive databases^22,23^ have made individual analysis impractical. Consequently, research has shifted toward designing high-throughput workflows to efficiently model 2D magnetic materials and identify candidates with promising *T*_c_ values.^14–19,24,25^

Density Functional Theory (DFT)^26,27^ remains a cornerstone for modeling 2D magnetic materials, allowing the determination of magnetic ground states.^28,29^ However, the reduced dimensionality of 2D systems poses unique challenges, as critical magnitudes are often weak, ranging from meV to μeV. This energy scale necessitates careful parameter tuning and introduces difficulties in capturing the coupled nature of electronic structure and spin order.^30^

Beyond determining magnetic ground states, DFT enables the extraction of exchange parameters through total energy calculations^30^ or related methods.^31^ These parameters can be integrated into complementary approaches like spin-wave theory,^32^ Metropolis Monte Carlo simulations,^33^ or Green's function methods^34^ to estimate critical temperatures.

In this review, we aim to equip readers with the theoretical and practical tools necessary for modeling 2D magnets using DFT. We begin by introducing basic principles of magnetism, followed by an overview of DFT and its associated challenges, such as electron delocalization and spin-order determination. Subsequently, we describe techniques for deriving parameters for magnetic Hamiltonians and estimating critical temperatures, providing a comprehensive comparison of methods tailored to different material anisotropies. This review serves as a foundational guide for researchers navigating the complex landscape of 2D magnetism.

## 2. Magnetic interactions in 2D magnets

It would be futile to attempt performing accurate simulations of the magnetic properties of two-dimensional magnets without first grasping their fundamental aspects. Fortunately, there are many books^32,35,36^ and comprehensive reviews^13,37^ that cover this topic in detail. In this section, we aim to highlight the essential concepts needed to understand the core challenges.

As an initial step, we begin by recalling that a generic Hamiltonian for electrons in a crystalline array of atoms can be written, within the Born approximation, considering three main contributions:

1 *Ĥ* = *H*_kin_ + *H*_lat_ + *H*_e–e_

where the first term corresponds to the kinetic energy of the electrons, the second to their interaction with the atomic lattice, and the last term models the electron–electron interaction arising from the Coulomb field. The first two terms are single-particle energies and can be significantly simplified using Bloch's theorem,^38,39^ which describes the system as a collection of non-interacting electrons with an effective mass and constrained momentum defined by the crystal structure. Therefore, the single-particle contribution favors delocalized electrons. The last contribution in the second quantization framework can be written as2
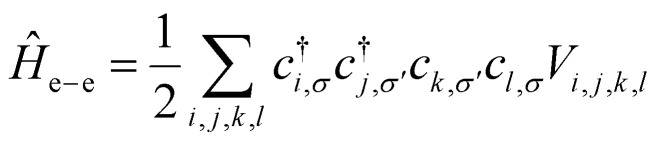
where *c*^†^_*i*,*σ*_ and *c*_*i*,*σ*_ represent the creation and annihilation operators of an electron in the *i*-th orbital with a given spin *σ* = ±1, respectively. These orbitals can be any single-particle basis, but for the purposes of this discussion, it is useful to think of them as localized orbitals at each atom in the crystal. In this sense, the Coulomb field term, characterized by the matrix 
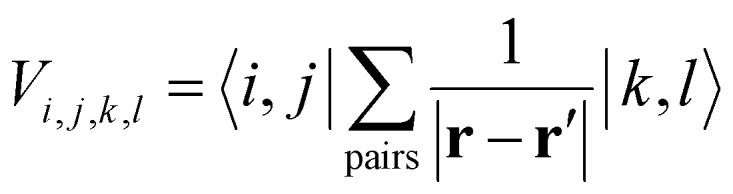
, contains the Coulomb interaction between pairs of electrons.

The Coulomb interaction can be further split into three contributions:

• **The direct or Hartree term***V*^H^_*ij*_ = *V*_*i*,*j*,*i*,*j*_, which, after some manipulation, can be expressed as the classical repulsive field between electron densities located at sites *i* and *j* of the lattice. Due to its classical origin, this term modulates the kinetic energy of the electron and alters the single-particle band structure of the system.

• **The exchange term***V*^Ex^_*ij*_ = *V*_*i*,*j*,*j*,*i*_, which arises due to the Pauli exclusion principle and favors lower energy for electrons with the same spin, owing to the antisymmetry of their orbital wavefunctions. This term is the essential source of ferromagnetism in materials.

• **Correlation terms**
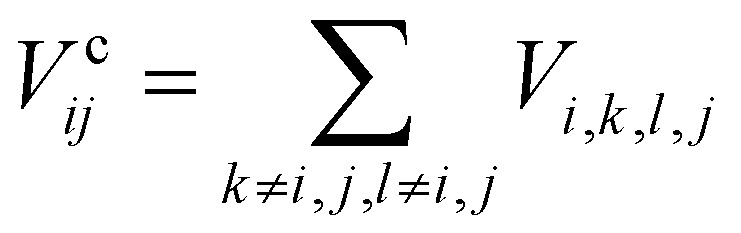
, which represent the dynamical response of an electron's transition from site *i* to site *j* to all the remaining electrons in the system. Correlations can substantially modify the ground state and induce phase transitions, such as superconductivity.

Dealing with the full many-body Hamiltonian is a formidable task. As a result, many alternative approaches and approximations have been developed to handle different aspects of the problem. The exchange interaction can be treated exactly within Hartree–Fock mean field theory, while the former plus correlation effects are approximated in different ways within density functional theory.^26,40,41^

In the subsequent sections we will briefly discuss some of the effective Hamiltonians which are used to model magnetism and its relation to the all electron Hamiltonian, since this will help to understand the proper way of modelling it.

### 2.1. Localized electrons and atomistic magnetic models

At the beginning of this section we remind that the operator associated with the exchange interaction can be expressed as:3
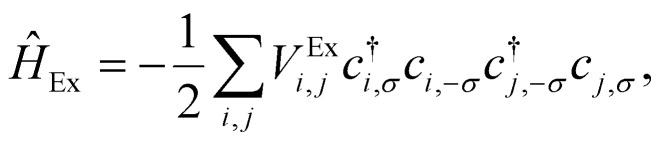
where *V*^Ex^_*i*,*j*_ is the exchange integral between sites *i* and *j*, and *c*^†^_*i*,*σ*_ and *c*_*i*,*σ*_ are the creation and annihilation operators for electrons at site *i* with spin (*σ*) = ±1. This form emphasizes the role of the Pauli exclusion principle and the antisymmetry of the wavefunction.

Using the number operator *n̂*_*i*,*σ*_ = *ĉ*^†^_*i*,*σ*_*ĉ*_*i*,*σ*_, we can define the local spin density operators at site *i*:4
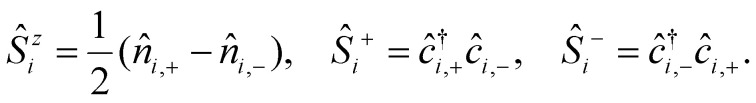


These operators represent the *z*-component of the spin and the spin–flip processes, respectively. Defining 2*Ŝ^x^_i_* = *Ŝ*_*i*_^+^ + *Ŝ*_*i*_^−^ and 2*iŜ^y^_i_* = *Ŝ*_*i*_^+^ − *Ŝ*_*i*_^−^ we can rewrite the exchange contribution to the Hamiltonian as:5
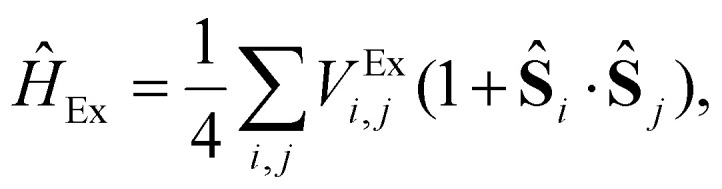
where *Ŝ*_*i*_ = (*Ŝ^x^_i_, Ŝ^y^_i_, Ŝ^z^_i_*) the spin vector operators at sites *i*.

For systems with tightly bound d- or f-electrons, the kinetic energy is significantly reduced due to the strong localization of the wavefunctions. Under these circumstances, the magnetic interaction is dominated by the exchange interaction between nearest neighbors. Moreover, the quantum spins in the Heisenberg model can be approximated as classical spins for large spin quantum numbers (*S* ≫ 1) given that quantum fluctuations become negligible. Similarly, at finite temperatures where thermal fluctuations dominate, or in systems with long-range magnetic order, the spin deviations are small enough to justify a classical description. Additionally, in Density Functional Theory codes, the magnetic moment of the atom is usually calculated by integrating the continuous spin density in a region centered around the atom, motivating even more the adoption of a classical approximation in which the spin operator is approximated by a continuous magnetic moment vector. This assignment of a continuous magnetic moment localized around an atom is what makes this kind of model and approximation receive the name of atomistic magnetic models. These approximations simplify the magnetic models and make it a versatile tool for studying magnetic properties in many systems.

Then, in practice, the magnetism in the material is studied with a parametric model, the most simple being the Heisenberg model:^36^6
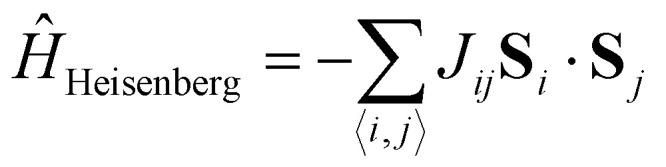
where *J*_*ij*_ is the isotropic exchange constant and **S**_*i*_ and **S**_*j*_ are the total magnetic moments around ions *i* and *j*. In practice, **S**_*i*_ and **S**_*j*_ are regarded as unit vectors so that the exchange constant can be expressed in energy units. The isotropic exchange is the dominant interaction in magnetic materials. The energy associated with the isotropic exchange interactions depends solely on the relative orientation of neighbouring spins and the existent overlapping between orbitals that form an interaction path.^30^ In most cases, it is way higher than the rest of the interactions and it controls the parallel or antiparallel alignment of the spins. With our notation, *J*_*ij*_ > 0 favours parallel alignment of the spins (ferromagnetism) where as *J*_*ij*_ < 0 favours antiparallel alignment (antiferromagnetism).

Depending on the symmetry of the system and the anisotropy of the exchange interaction, the Heisenberg model can be reduced to simpler forms, the most prototypical being:^13,37^

• **Ising model**: the Heisenberg model transitions to the Ising model^42^ when there is strong anisotropy along one spin direction (*e.g.*, *z*-axis), making the spin components perpendicular to this axis negligible. The resulting Hamiltonian becomes:7
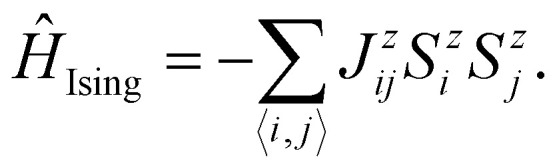
here, 〈*i*,*j*〉 stands for a summation that runs over the nearest neighbors of *i*.

• **XY model**: the Heisenberg model reduces to the XY model when the spin interactions are restricted to the *x*–*y* plane, with negligible contributions from the *z*-component. The Hamiltonian in this case is given by:8
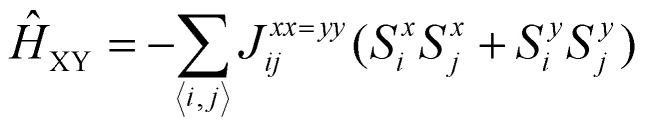


With an exchange constant *J*_*ij*_ that is independent of the direction *x* or *y*. In this case, the magnetism is said to be of the easy-plane type.

This rationale can be extended to more complex interactions, giving birth to a variety of atomistic magnetic models in which the interactions are modeled by means of parameters that can be calculated from first principles. Again, the adjective “atomistic” is used in this context to highlight the fact that this construction assumes the localization of the magnetic moments around the atoms. In addition to the isotropic exchange interaction discussed above, some important effects to keep in mind are those arising from spin–orbit coupling interactions, which introduce anisotropy in the system. Some of these are:^13,37^

• **Single ion anisotropy (SIA):**9
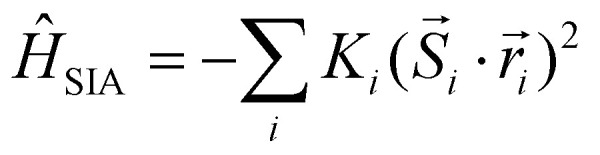
where the vector 
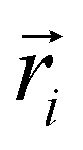
 denotes the direction that minimizes the total energy contribution for the magnetic moment 
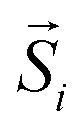
, equivalently, the preferential alignment for the spin 
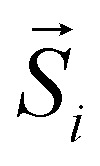
. 
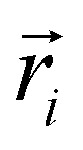
 is called the easy axis. The parameter *K*_*i*_ is the SIA energy for the spin 
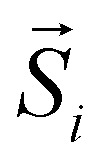
. Single-ion anisotropy is originated from the interaction between the SOC and the crystal field. It tells us about the spin interacting with the environment and then it is a local property, that involves a magnetic atom and the surrounding coordination sphere formed commonly by the ligands.

• **Anisotropic exchange:**10
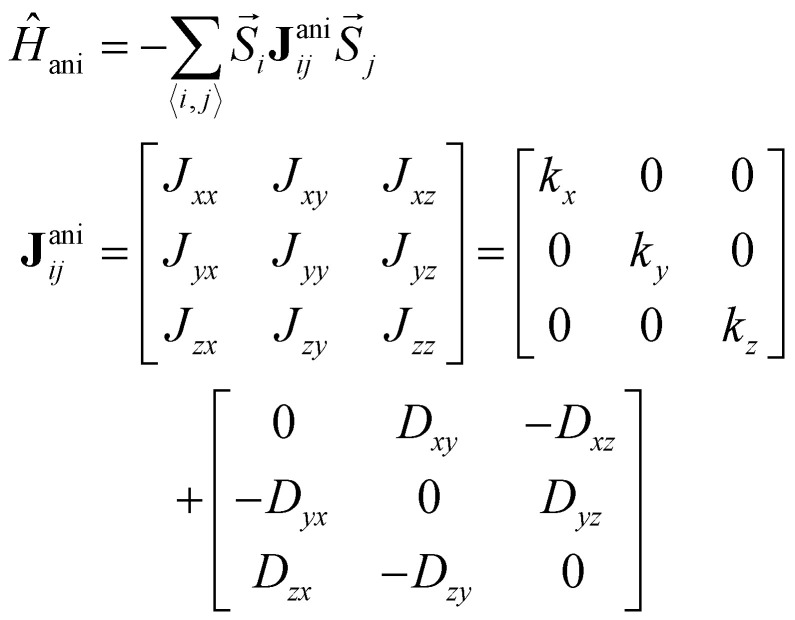
where **J**^ani^_*ij*_ is a 3 × 3 matrix. The anisotropic exchange tensor can be divided in two different contributions. The first one is the so called two-ion anisotropy,^41^ given by the terms *k*_*i*_ on the diagonal. When the two ion-anisotropy goes along bond directions, it is called Kitaev exchange. These terms introduce two-spins exchange anisotropy. The other contribution is given on the antisymmetric terms of the tensor. This contribution is the so-called Dzyaloshinski–Moriya interaction (DMI)^43–45^ and it can be rewritten as:11
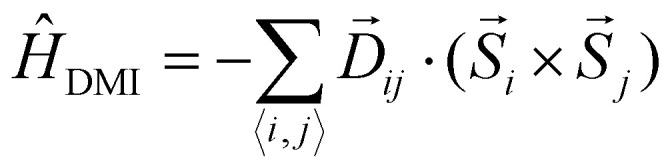
where 
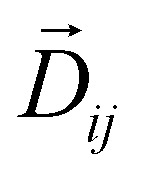
 is the so-called DMI vector. The DMI interaction is usually much weaker than the isotropic exchange interaction and therefore, it introduces a small canting of the spins with respect to the direction forced by the isotropic exchange.

These are not the only interactions frequently used in atomistic magnetic Hamiltonians. Some other less frequent but still worth mentioning are:^46^

• **Biquadratic exchange:**12
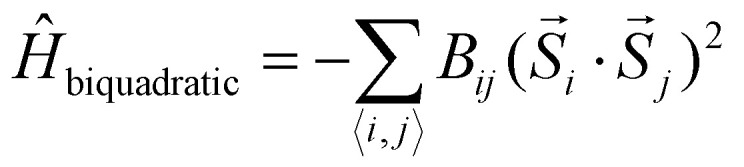


Which differs from the isotropic exchange in the square dependence on the dot product of the spins. Therefore, when *B*_*ij*_ > 0, the biquadratic exchange favours a collinear alignment independently on whether it is ferromagnetic or antiferromagnetic. However, its dependence on *ij* can help to lift the degeneracy of states that would be degenerate with a Heisenberg Hamiltonian. Biquadratic exchange is usually expected to be less important than its bilinear counterpart (isotropic and anisotropic exchange). Nevertheless the work of Kartsev *et al.*^47–49^ showed in detail how it can have a value of an important fraction of the bilinear exchange. Additionally, magnetic properties such as the Curie temperature were calculated for different 2D magnets with and without biquadratic exchange; and the inclusion of this interaction was found to lead to the best agreement with experimental measurements. The inclusion of the biquadratic exchange also showed a big impact on the shape of the spin-wave spectra.

• **Four-spin interaction:**13
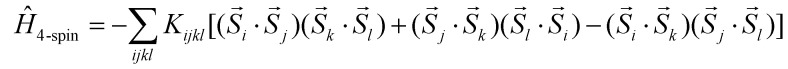


Similarly to the Biquadratic exchange, the four-spin interaction serves to break the degeneracy on the Heisenberg Hamiltonian.

• **Dipole–dipole interactions:**14
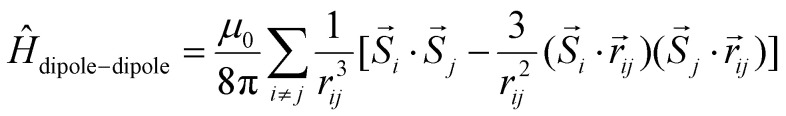
where 
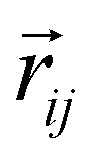
 is the vector connecting sites *i* and *j*. Magnetic dipole–dipole interactions help to stabilize magnetic orders, specially in 2D vdW systems.^50–52^ However, these interactions are usually negligible when calculating critical temperatures since their value is much smaller than the exchange interactions. This energy contribution is usually refered as shape anisotropy and it is SOC independent.

### 2.2. Itinerant electrons and stoner magnetism

While the Heisenberg and the atomistic models describe localized magnetic moments around the atoms, Stoner magnetism provides a framework for understanding magnetism in itinerant electron systems. In these systems, the magnetic moments arise not from localized spins but from the collective behavior of delocalized electrons in a metallic band structure. The origin of this magnetism lies in the interplay between the kinetic energy of electrons and their Coulomb interaction, which can be expressed within a mean-field framework.

As previously mentioned the Kinetic and lattice contribution can be combined into a single particle non-interactive Hamiltonian15
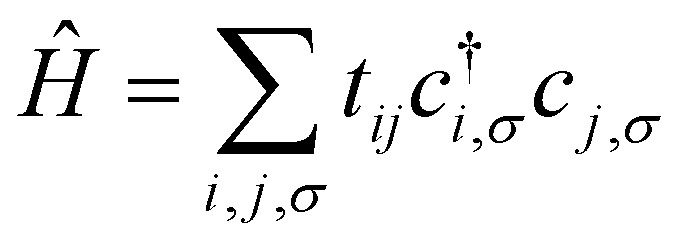
where: *t*_*ij*_ is the hopping integral between sites *i* and *j*, describing the kinetic energy of the delocalized electrons. Moreover, in systems where correlations are negligible, one can approximate the Coulomb interaction as an onsite-term16
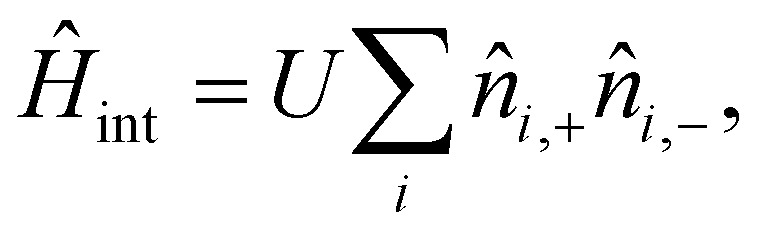
where *U* is the on-site interaction strength. Using the mean-field approximation, this term can be linearized as:17
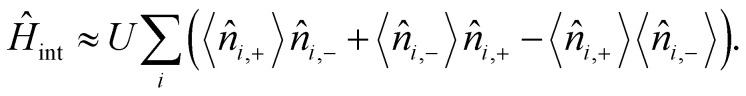


Here, 〈*n̂*_*i*,*σ*_〉 represents the average occupation for spin *σ*. The difference in spin populations defines the magnetization:

18 *M* = 〈*n̂*_*i*,+_〉 − 〈*n̂*_*i*,−_〉.

The competition between the kinetic energy, which favors equal spin populations, and the exchange interaction, which lowers the energy for unequal populations, leads to the Stoner criterion for ferromagnetic instability:

19 *UD*(*E*_F_) > 1,

where *D*(*E*_F_) is the density of states at the Fermi energy. When this condition is satisfied, the system favors a spin-polarized ground state, resulting in spontaneous magnetization.

Stoner magnetism arises because the exchange interaction reduces the energy for electrons with parallel spins, lowering the total energy of the system when the spin populations are unequal. This mechanism is distinct from the localized spins in the Heisenberg model, as it depends on the delocalized nature of the electronic wavefunctions.

The Stoner model provides a simple and intuitive picture of itinerant magnetism, particularly in metallic systems such as ferromagnetic transition metals (*e.g.*, Fe, Co, Ni). However, it neglects electron correlation effects beyond the mean-field approximation, which can significantly influence magnetic properties, particularly in strongly correlated systems. Extensions to the Stoner model, such as dynamical mean-field theory (DMFT), address these limitations and provide a more complete description of itinerant magnetism.

### 2.3. Magnetic order and the role of exchange interaction

The exchange interaction is the fundamental mechanism governing the emergence and type of magnetic order in materials. Depending on the nature of the electronic system and the interplay of additional interactions, different forms of magnetic order can arise. This section provides an overview of three main categories of magnetic order: ferromagnetism, antiferromagnetism, and non-collinear magnetism, discussing their origins within localized and itinerant frameworks and their relation with the exchange interaction.

• **Ferromagnetic order**. Is characterized by the parallel alignment of magnetic moments, resulting in a net macroscopic magnetization. The key condition for ferromagnetic order is that the exchange interaction favors parallel spin alignment. In localized electron systems, ferromagnetism arises due to direct exchange or superexchange mechanisms while for itinerant systems, ferromagnetism is driven by the Stoner criterion.

• **Antiferromagnetism order**. Is defined by an alternating spin alignment that results in no net magnetization. This order arises when the exchange interaction favors antiparallel spin alignment (*J*_*ij*_ < 0). In localized systems, antiferromagnetism is often stabilized by superexchange interactions. For example, in Mott insulators, virtual hopping processes between neighboring sites lower the energy when spins are antiparallel. In itinerant systems, antiferromagnetism can emerge due to Fermi surface nesting, where certain wavevectors **q** connect regions of the Fermi surface. This enhances the susceptibility at **q**, leading to spin-density waves (SDWs) with periodic modulation of spin density. In some cases, *J*_*ij*_ < 0 produces an antiparallel alignment of the spins but with a nonzero net magnetization because one of the atoms of the two sublaticces has a greater magnetic dipole moment. These cases lead to the so-called ferrimagnetic order.^53^

• **Non-collinear magnetic orders**. Non-collinear systems are arrangements of magnetic moments that are not oriented in the same direction. The most fundamental case of non-collinear magnets are the in-plane systems, where the spins are contained in an easy plane, with a hard perpendicular axis. Non-collinear magnetism can also present exotic configurations giving rise to phenomena such as skyrmions or spin spirals that arise when the spins form angles with respect to each other rather than aligning parallel or antiparallel. These exotic orders tend to be stabilized by other kind of effects, that compete with the isotropic exchange interaction such as the DMI induced by spin–orbit coupling, magnetic frustration due to competing exchange interactions or the shape anisotropy originated by the magnetic dipoles.

**Fig. 1:**
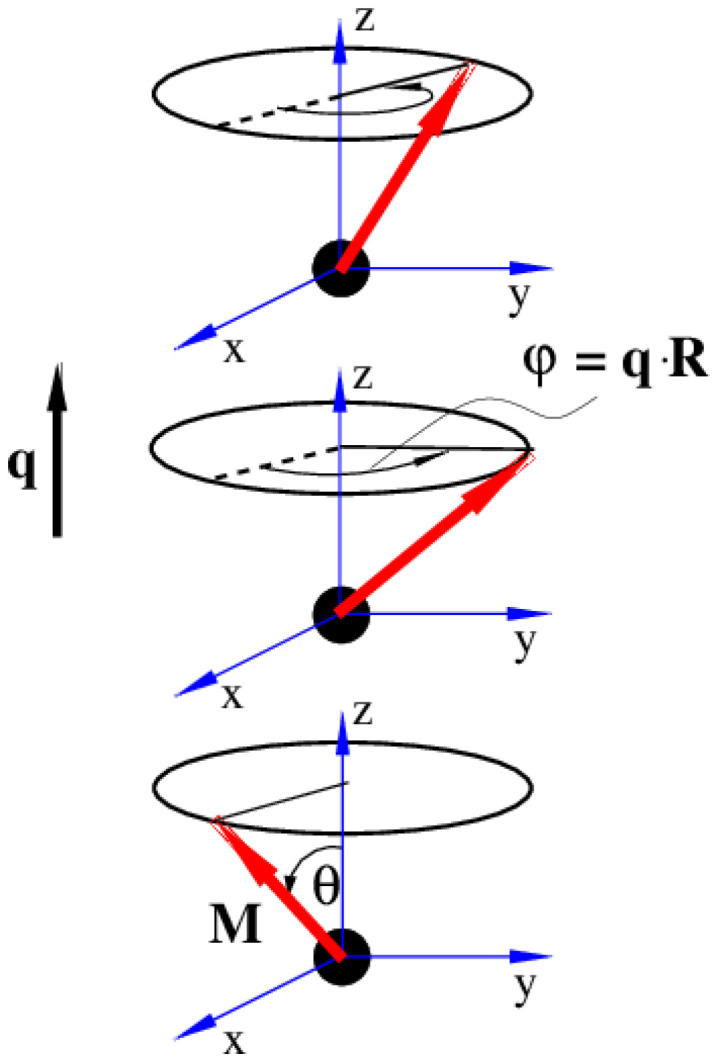
Sketch of a spin-spiral. The precession axis is taken to be the *z*-axis. The cone angle *θ* is specified as the angle between the spin direction and the precession axis. The spin-spiral moves along the direction of the **q** vector and the difference in phase between two consecutive spins is Δ*φ* = **q**·**R** where **R** is the vector connecting the two sites. Figure extracted from ref. 57. Reprinted figure with permission from S. Mankovsky, G. H. Fecher and H. Ebert, *Phys. Rev. B*, 2011, **83**, 144401. Copyright 2025 by the American Physical Society.

A special case among the possible magnetic configurations is the case of spin-spirals. These states have spins that rotate with respect to the initial alignment a certain angle over a specific direction given by the spin-spiral wavevector **q**. Spin spirals have a wavelength 
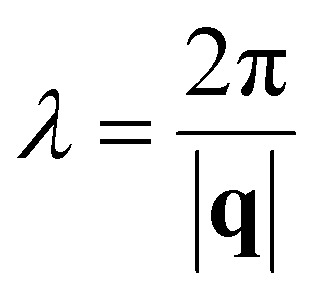
 separating two sites with the same spin direction (phase). A spin spiral showing these features is sketched in Fig. 1. Spin spirals are special because incommensurate spin-spirals such as the ones in 3D γ-iron^54–56^ or long wavelength spin-spirals cannot be handled in any exploratory supercell approach. Additionally, collinear magnetic order can be regarded as a particular case of spin spiral order when the wavevector lies on the Γ point of the first Brillouin zone or at some points of its high symmetry path.^29^

### 2.4. Special features in 2D magnetism

So far, our discussion about magnetism has been independent of the dimensionality of the material. However, the dimensionality of the 2D magnetic materials makes magnetism different from the 3D counterparts. One of the most notable differences arises as a consequence of the Mermin–Wagner theorem.^4^ This theorem states that for a 2D material with infinite size and modelled with an isotropic Heisenberg model with finite range interactions, long range magnetic order is not possible at nonvanishing temperature. This theorem is sometimes formulated saying that long range magnetic order is not possible for a 2D material with infinite system size and with any continuous symmetry.^13,58^ However, this theorem does not contemplate what happens when anisotropy is present.

What we know as of today is that anisotropy can stabilize magnetic order at nonvanishing temperature as shown for CrI_3_ experimentally^1^ in 2017. This material presents ferromagnetic behaviour up to 45 K that is stabilized owing to the presence of exchange anisotropy in the *z*-direction, causing an out of plane spin orientation that produces ferromagnetism.^6^ Monolayer Fe_3_GeTe_2_ presents a similar feature with a ferromagnetic phase driven by strong out of plane anisotropy up to 130 K.^3^ Hence, in 2D magnets, the isotropic exchange usually dominates the parallel/antiparallel alignment of the spins but the long range magnetic order is stabilized by a source of anisotropy.

Another special feature of 2D magnets is that the diverse composition of 2D van der Waals materials tends to present different species that do not directly participate in the exchange. The presence of these ligands foments indirect mechanisms that allow interactions between neighbouring metallic centers that are too far to interact directly. The overlapping between metal–ligand–metal orbital connections creates new channels of interactions that are called indirect or super-exchange interactions.^37^ Moreover, more complex overlapping of orbitals can be present in these systems originating super–super or even super–super–super exchange.

In the end, the special keys about 2D magnetism are the important role anisotropies play and the variety of possible exchange paths. Consequently, the core of its *ab initio* studies focuses on the identification of the many possible mechanisms that produce that anisotropy/exchange and the calculation of its strength. When doing so in the framework of Density Functional Theory (DFT), some difficulties appear due to two main factors:

• The intrinsic problems within DFT††And mainly those of the current approximate exchange correlation functionals. when it comes to the accurate modelling of certain interactions such as exchange, correlations or long range van der Waals interactions.

• The reduced dimensionality of 2D materials makes the energy scales of the anisotropies very low: from meV to μeV. For example, the isotropic exchange constant, which is usually the greatest in magnitude, is usually on the order of the meV where as in 3D materials can be of the order of 100 meV. The calculation of these parameters is strongly influenced by the structure and Hubbard parameters and can also be importantly influenced by more fundamental computational details, such as the pseudopotentials or the approximation used to describe the exchange correlation functional, which is especially important in the case of itinerant magnets.

In the following section, we give a brief introduction to Density Functional Theory and provide a compilation of the necessary tools to simulate 2D magnetic materials within DFT.

## 3. Introduction to DFT

The hydrogen atom was the first important benchmark to illustrate the success of quantum mechanics and the Schrödinger equation. Regretfully, more complex systems such as heavier atoms or solids were still intractable at the time, mainly because of the many body nature these systems. As Dirac said, *the fundamental laws necessary for the mathematical treatment of a large part of physics and the whole of chemistry are thus completely known, and the difficulty lies only in the fact that application of these laws leads to equations that are too complex to be solved*. In the particular case of a solid, solving the Schrödinger equation for a wavefunction *Ψ*(**r**_1_…,**r**_*N*_) with *N* spatial coordinates (3*N* scalar variables) is completely out of reach, even computationally. This is why the birth of Density Functional^26,27,40,59,60^ (DFT) is one of the most important milestones in condensed matter physics since it provides an exact theory to describe many-body systems with interacting particles.^27^

**Fig. 2:**
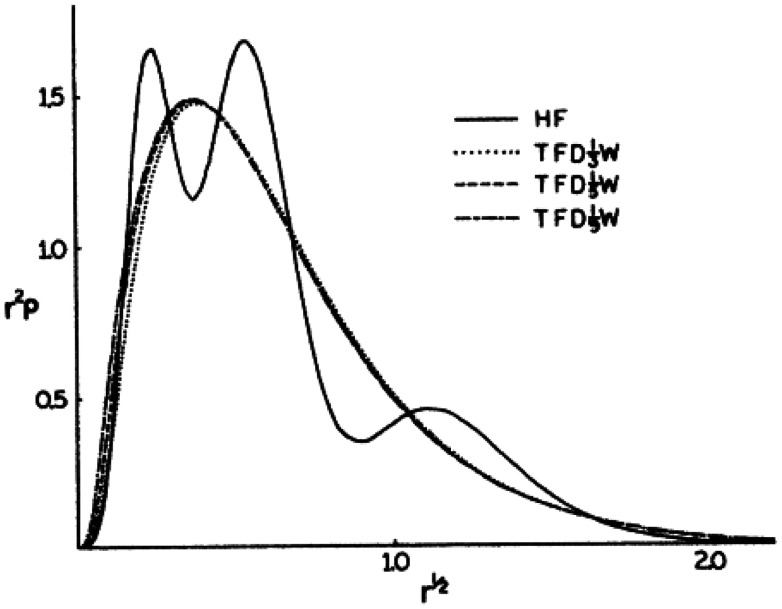
Comparison of the electron density of Argon using the LDA-Thomas–Fermi model and Hartree–Fock. The TF approach results in an overall good description of the electron distribution, but importantly fails in the description of the peak structure. Figure extracted from ref. 64. Reprinted figure with permission from W. Yang, *Physical Review A*, 1986, **34**, 4575. Copyright 2025 by the American Physical Society.

The first attempts to establish an alternative formulation to the Schrödinger equation based on the use of total electronic density *n*(*r*) and the density functional were introduced in 1927–1930 by Thomas, Fermi and Dirac.^61–63^ This formulation laid the origins of DFT, with the so-called Thomas–Fermi (or Thomas–Fermi–Dirac) model that introduced the LDA approximation to describe the kinetic energy of electrons. The TFD model paved the way for significant advances in the history of electronic structure, although this nascent model was not able to provide the required quantitative accuracy (Fig. 2) and did not provide a formal and complete theory of electronic structure.

It was in the 1960s, when the ideas behind an alternative formulation of the electronic problems, based on the total charge density of the system, were formally written, giving birth to DFT in 1964–1965 in the hands of Hohenberg, Kohn and Sham.^26,59,65^ The solid principles of DFT are in the present expressed in terms of the Hohenberg–Kohn theorems (HK). The first HK theorem proves that for any system of electrons interacting *via* Coulomb interactions in an external potential, the external potential is fully determined (up to a constant shift) by the ground state electron density *n*_0_(**r**). The second theorem states that a functional of the energy *E*[*n*] exists for any external potential, that the ground state energy is its global minimum and the electron density that minimizes it is *n*_0_(**r**). Unfortunately, the theorem does not give any hint about how to obtain such functional *E*[*n*], but we can express the total energy without loss of generality as:^27^20
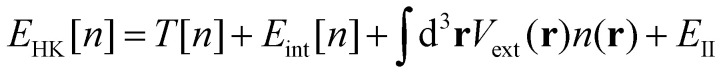
where *T*[*n*] is the kinetic energy contribution, *E*_int_[*n*] is the contribution of the electron–electron interaction, *E*_II_ is the repulsion between cores and the term of the integral corresponds to the external potential contribution (core-electron interaction).

The important consequence of these two theorems comes after the realization that the wavefunction of the system is determined once the external potential is known (since the form of the Coulomb repulsion is known already). But the first theorem states that the external potential is determined by *n*_0_(**r**). As a result, knowing the ground-state electronic density *n*_0_(**r**) is equivalent to knowing the wavefunction of the system. This establishes the electronic density as the fundamental variable of the system, determining all its properties. And this result is extremely convenient since the electronic density is a function of three variables where as the wavefunction depends on 3*N* variables.

The next key result came in 1965 by Kohn and Sham.^26^ They proposed to assume that there is a system of non-interacting particles, called auxiliary system, with the same electronic ground-state density as the real system. Then, to obtain *n*_0_(**r**), we can work with the auxiliary system and the many-body effects can be incorporated *via* an external exchange–correlation potential expressed as a functional of the density. This way, the total energy of the auxiliary system can be expressed by rewritting (20) as:21
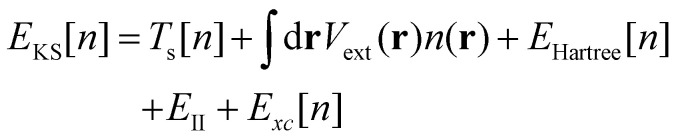
where *T*_s_[*n*] represents the kinetic energy of the particles of the auxiliary system, *E*_xc_[*n*] represents the contribution of the many-body effects written as a functional of the density and *E*_Hartree_[*n*]:22
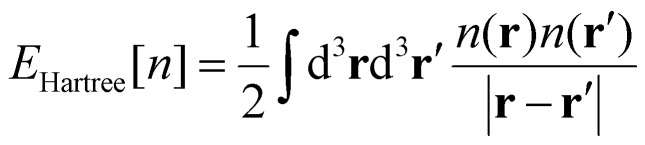


Is the classical interaction energy of the charge density with itself. Since (21) is just (20) rewritten, by making them equal and solving for *E*_xc_[*n*]:

23 *E*_xc_[*n*] = *T*[*n*] − *T*_s_[*n*] + *E*_int_[*n*] − *E*_Hartree_[*n*]

Expression (23) shows explicitly how the exchange–correlation functional aims to capture all the many-body effects of the real system in an external potential so that the auxiliary system can be of non-interacting particles. Unfortunately, there is no way to know the exact form of the exchange–correlation functional and DFT ends up being exact in theory but approximate in practice. Most approximations are based on the exchange–correlation energy density of the uniform electron gas^26,27,66,67^ and then, the success of DFT comes from how extremely simple approximations to the exchange–correlation functional give very good results for many systems making DFT extremely popular.^68^

The practical development of the Kohn–Sham approach leads to a set of equations:24
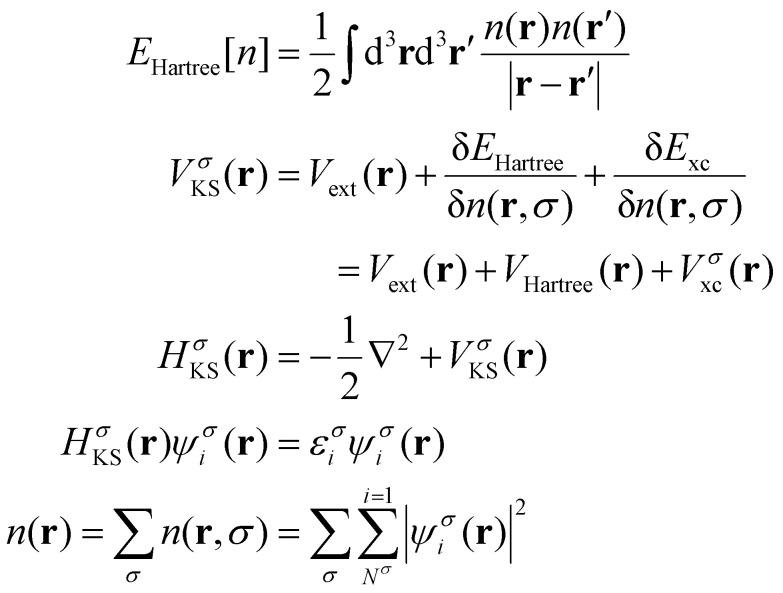


That must be solved self-consistently in the given order since the Hartree term and the exchange–correlation term depend on the electronic density. The eigenstates *ψ^σ^_i_* are the so called Kohn–Sham states. These states are in principle nothing else but the eigenstates of the auxiliary system but the success of DFT has made standard the description of the systems in terms of these single particle states. Nevertheless, we remark that these states do not necessarily have any connection with reality, they simply provide a useful and simple language *via* single particle states that serve to describe a complex system. In practice, the Kohn–Sham states are expanded in a basis set either made by plane waves, localized atomic orbitals or a smart combination of both.

To achieve an accurate electronic structure using practical implementations of DFT, several considerations must be taken into account. These include the choice of an appropriate exchange–correlation functional, the selection of pseudopotentials, adequate convergence parameters, the inclusion of noncollinear spin configurations^69^ and spin–orbit coupling,^70,71^ as well as smearing techniques.^72–74^ Incorporating van der Waals (vdW) interactions can be essential for modeling 2D materials, van der Waals heterostructures, and layered systems, as these weak forces strongly influence structural stability and electronic properties.^75–77^ They are crucial for capturing interlayer coupling and stacking-dependent behaviors, making them indispensable for such simulations.

While most of these settings are standard features of any DFT tutorial^78^ and will not be elaborated here, we consider it critically important to address the delocalization problem inherent to all approximations of the exchange–correlation functional^79–82^ and discuss potential improvements due to their inherent relation to magnetic materials as we will discuss below.

The delocalization error is a well-documented limitation of standard functionals and becomes particularly problematic in systems with strongly localized d and f electrons.^83–85^ These electrons play a key role in the properties of magnetic materials, as they directly influence exchange interaction, the fundamental mechanism driving magnetic ordering. Furthermore, the Coulomb interaction, which underpins these exchange effects, also determines the degree of electron localization, closely linking it to magnetic phenomena.^86,87^

### 3.1. Improving the localization by using DFT+*U*

It was shown back in 1982 that the exact exchange–correlation functional follows the constraint that the total energy behaves in a linear piecewise manner as a function of the total number of electrons.^88^ It has been covered already in previous reviews^79–82^ how the violation of this constraint makes approximate-exchange correlation functionals delocalize electrons exceedingly, leading to poor description of the electronic structure for certain systems, huge underestimations of the bandgap or even the contradictory prediction of a metal instead of an insulator. Some typical examples of this failure are metal oxides such as NiO,^84,89,90^ FeO and MnO.^91–93^ These examples have all in common that the partially filled strongly localized d or f shells play an essential role in the electronic structure. The approximate exchange–correlation functionals lead then to a particularly bad description of these *l*-shells. In order to correct this prominent excess of delocalization, DFT+*U* was invented.^94^ Inspired by the Hubbard model,^95^ DFT+*U* aims to favor localization of the electrons by introducing an energy penalty when the orbitals have fractional occupation. This way, DFT+*U* favours cases in which the orbitals are either with one electron or empty, avoiding the cases in between. This is done by rewritting the exchange–correlation functional as:^71,86^25
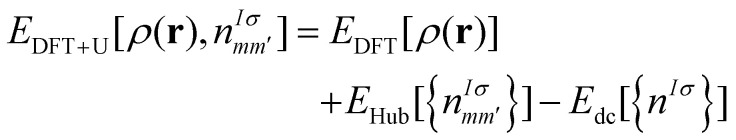
where *E*_DFT_[*ρ*(*r*)] is the total energy given by the used exchange–correlation functional, 
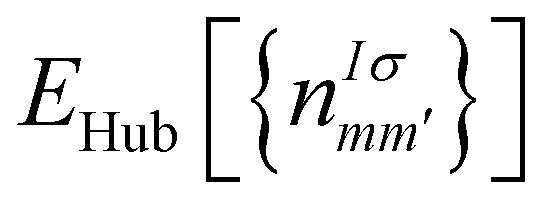
 is the Hubbard-like correction and *E*_dc_[*n*^*Iσ*^] is the double counting term that aims to remove the contribution of the corrected orbitals from *E*_DFT_[*ρ*(*r*)]. By construction, the computational cost of the DFT+*U* approach is practically the same as a usual DFT calculation. *I* is an index running over the atoms with correlated/localized electrons, and the indices *m* and *m*′ run over the localized states of atom *I*. Most of the times, *m* and *m*′ run over states with the same angular momentum quantum number *l i.e.* they belong to the same *l*-shell. 
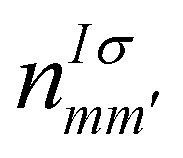
 are the occupation numbers of the localized states so that 
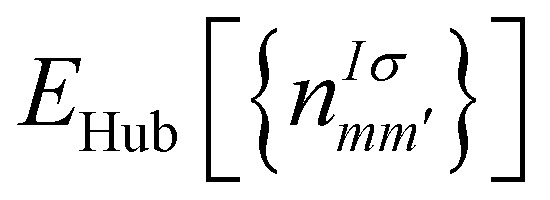
 adds an energy correction depending on how populated they are. The details on how 
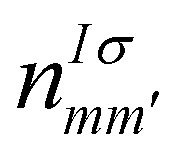
 is calculated are code-specific, but a common way is projecting the Kohn–Sham eigenstates {*ψ*^*σ*^_**k***i*_} on a basis set of localized functions {*φ^I^_m_*}.^71,86^

This aspect results crucially important to consider in the modelling of materials that present strongly localized electrons, such as the ones present in d or f orbitals. Given these kind of orbitals tend to present natural open shells with unpaired electrons, they are directly behind the origin of magnetism and thus, a correct description of the electron localization is fundamental for the computation of magnetic properties of 2D materials.

From the different methodologies available, Hubbard corrections are among the most significant methodologies to improve the approximation of the electron behaviour in the density functional and have demonstrated to provide an excellent compromise between improvement and computational efficiency,^86^ being implemented in most of the DFT packages (see Table 1).

**Table 1:** List of popular Density Functional Theory (DFT) codes with capabilities and features. These features have been compiled after searching through each code's manual

DFT code	License	Basis set	sc-DFT	GBT	vdW corrections	DFT+*U*	*U* obtention	DFT+*U*+*V*	*V* obtention
VASP^150,151^	Commercial	Plane-waves (pseudopotentials)	Yes^166^	Yes	DFT-D2^167^	Yes	Yes^100^	No	No
					DFT-D3^168,169^				
					DFT-D4^170^				
					Method of libMBD^171^				
					Tkatchenko–Scheffler^172–174^				
					MBD@rSC^175,176^				
					MBD@rSC/FI^177,178^				
SIESTA^179^	GPL (academic)	Localized atomic orbitals (pseudopotentials)	Yes^134^ (but not public)	Yes (but not tested)	DRSLL^180^	Yes^71^	No	No	No
					LMKLL^181^				
					KBM^182^				
					C09^183^				
					BH^184^				
					VV^185^				
FLEUR^152,153^	GPL (academic)	FP-LAPW (all electron)	Yes	Yes^186^	DFT-D3^168,169^	Yes^187^	No	No	No
					DRSLL^180^				
Quantum ESPRESSO^114,118^	GPL (academic)	Plane-waves (pseudopotentials)	Yes	No	DFT-D2^167,188^	Yes	Yes^100,105^	Yes^87^	Yes^100,105^
					DFT-D3^168^				
					Tkatchenko–Scheffler^172^				
					MBD^176^				
					XDM^189,190^				
OPENMX^154,155^	GPL (academic)	Localized atomic orbitals (pseudopotentials)	Yes	Yes^157,158^	DFT-D2^167^	Yes^191,192^	No	No	No
					DFT-D3^168,169^				
GPAW^146,147^	GPL (academic)	Plane-waves (pseudopotentials)	Yes	Yes	DRSLL^180,193^	Yes	No	No	No
					LMKLL^181^				
					BH^184^				
					KBM^182^				
					C09^183^				
ABINIT^194^	GPL (academic)	Plane-waves (pseudopotentials)	Yes^195^	No	Yes^196^	Yes^197^	Yes^100,108^	No	No
					DRSLL^180^				
					DFT-D2^167^				
					DFT-D3^168^				
CASTEP^198^	Commercial	Plane-waves (pseudopotentials)	Yes	Yes	Tkatchenko–Scheffler^172^	Yes	Yes	No	No
					MBD@rSC^175,176^				
					DFT-D2^167^				
					DFT-D3^168^				
					OBS^199^				
					XDM^200^				
ELK^201^	GPL (academic)	FP-LAPW (all electron)	Yes	Yes	No	Yes	No	No	No
WIEN2K^202^	Academic (paid)	FP-LAPW (all electron)	No	No	DRSLL^180^	Yes	No	No	No
					LMKLL^181^				
					SCAN+rVV10^203^				

Inspired by the role of the Hubbard model in the description of strongly correlated electrons, DFT+*U* initially aspired to improve the description of the electrons present in strongly localized orbitals. However, the active implementation of the Hubbard *U* parameter in the exploration of new materials, has revealed to the community that DFT+*U* can have a broader impact, by restoring the piecewise-linear relationship between the total energy and the electronic occupations, thereby helping to mitigate the errors arising from the violation of this constraint.

In this sense, DFT+*U* does not directly address localization and strong correlations. Instead, it helps to solve a more fundamental issue: the piecewise-linear constraint of the exact exchange–correlation functional. The violation of this constrain heavily affects electron localization which is especially relevant in these cases and more importantly, in 2D magnetism in which electron localization and magnetism are extremely correlated.

The Hubbard corrective term and the double counting term can take different expressions depending on the formulation,^86,96,97^ but we want to highlight how one of the most simple formulations resembles the Hubbard model and the energy penalty for partial occupation of the orbitals within the same *l*-shell:26
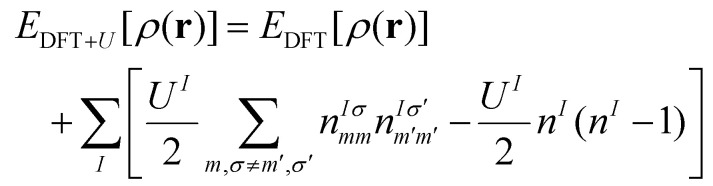


We can see a similarity between the product 
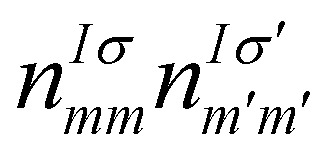
 and the Hubbard term 
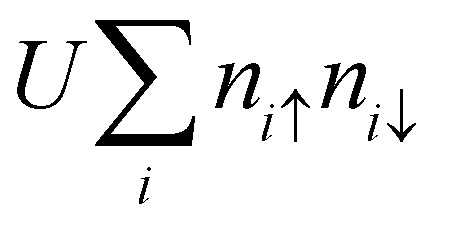
 of the Hubbard model. Here 
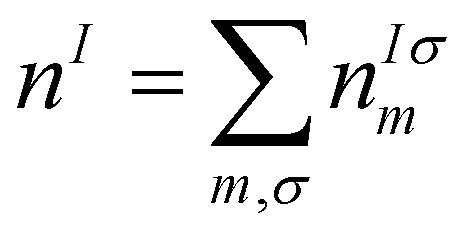
 is the sum of all the occupation numbers of the localized states of atom *I*. The second and third terms of the right hand side of the equality correspond to the energy correction and the double-counting correction respectively. This equation shows that the new exchange–correlation functional has a set of parameters, the set of {*U*^*I*^}, one for each atom with localized orbital states. These parameters are called the Hubbard parameters or simply the *U* parameters. They are nonnegative and they represent the mean coulomb repulsion energy between two electrons in the same atom and belonging to the same *l*-shell. As one can see from the second term in the right hand side of (26), the correction term introduces an energy penalty if the occupation numbers are not 0 or 1, favouring localization. The energy penalty has a value of *U*^*I*^ independently of the value of the quantum number *m*. This assumption is justified when the localized orbitals retain atomic-like symmetry *i.e.* spherical symmetry.^86^ Therefore, the DFT+*U* approach has a worse performance when there are physical features removing the approximate equivalency of the orbitals with the same *l* such as strong SOC or crystal field.^86^

DFT+*U* schemes are parametrized by a *U* input parameter for every single *l*-shell to which the correction is applied, removing the *ab initio* character of the simulation. This introduces a problematic situation from a first-principles perspective. Over several years of intense research, different alternatives to deal with this parameter have been explored by the community. Its consistent obtention is indeed of extreme relevance since the value of *U* can have great influence on the observed results.^25,98,99^

One of the most extended approaches involves empirically extracting the Hubbard *U* through the fit to specific properties computable within the DFT framework. Usually one of the most representative target parameters that can be found in literature, is the gap of an electronic band structure. Despite the correction introduced by DFT+*U* has proved to play an important role in the improvement of the electronic structure description,^86,100^ DFT was never designed to predict spectroscopic properties. The solely use of a band gap to benchmark the correct DFT+*U* modelling of a material is a disregarded approach that in addition, strongly limits the predictive capabilities of the first principles approach. As it will be discussed below, there are many other properties that result more reliable and that can considered in addition to the band gap to ensure a correct modelling of the materials under the framework of DFT+*U*.

A more complete approach consists of performing an extended exploration of the Hubbard *U*, broadening the window to the computation of different important properties in addition to the electronic structure, such as magnetic moments, exchange parameters, energies and shape of magnon dispersions to name a few. A very robust addition to this approach, is to scan also other external conditions such as, the geometrical distortions or the charge modifications induced by strain and electrostatic doping simulations, respectively. The computation of different sophisticated properties and their evolution under these external stimuli, easily accessible from the DFT calculations, provides a very valuable dataset of information. The critical analysis of this information is an effective technique for assessing the DFT+*U* model and determining the Hubbard parameter from a broad perspective that accounts for both the model's performance and limitations.

Fortunately, the *U* parameter represents the average Coulomb repulsion energy between electrons within the same *l*-shell and its physical meaning can be exploited in order to design ways to estimate it. Hence, a third approach to determine it is simply calculating it with a systematic method. The most famous methods are the Hartree–Fock (HF) approach to calculate it,^101,102^ the linear response approach,^86,100,103–105^ the constrained random phase approximation^106–108^ (cRPA) and some machine learning approaches^109,110^ that include Bayesian optimization.^111,112^ All these methods propose a fully systematic manner to calculate *U*, making the DFT+*U* flavour a zero free parameters approach and going back to the first principles philosophy.

**Fig. 3:**
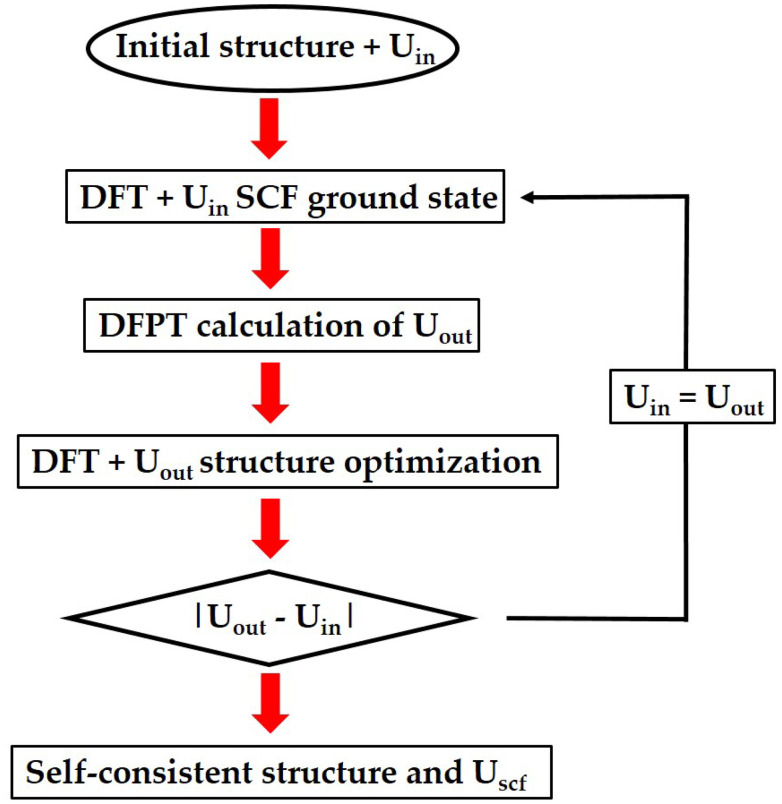
Scheme of the algorithm to obtain the *U* parameter in a self-consistent procedure.

The HF approach basically calculates the average Coulomb repulsion energy from unrestricted HF calculations. The linear response approach aims to tune the *U* parameter so that the total energy as a function of the number of electrons gets to be linear-piecewise, following the constraint of the exact functional.^88^ This linear response approach has the advantage of offering a fully self-consistent way of calculating the parameters within DFT. Moreover, it has been reformulated recently in Density Functional Perturbation Theory (DFPT),^103,104,113^ offering better performance and accuracy and even a fully automated package to compute it called HP^105^ available as part of the DFT code QUANTUMESPRESSO.^114^ This process is shown in Fig. 3. The structure is initially relaxed for an initial value of *U* = *U*_in_, which can be zero. Then, the ground state is obtained and a value of *U* = *U*_out_ is calculated for that ground state. Afterwards, a new ground state is calculated with the obtained *U*_in_ = *U*_out_. For this new ground state, a new value of *U*_out_ is calculated. The process is then repeated until the difference between the input *U* and the output *U* is less than a threshold.

The DFT+*U* approach serves to describe more localized regimes. Following a similar idea, DFT+*U*+*V* was born:^87^ a different approach capable of describing more general localization regimes by introducing an energy term that favours partial occupation of orbitals of different atoms that are hybridized. This additional parameter adds more flexibility to force the linear-piecewise constraint by allowing a more general case of localization to be corrected and improved. The DFT+*U*+*V* correction without the double counting correction is:^86,87^27
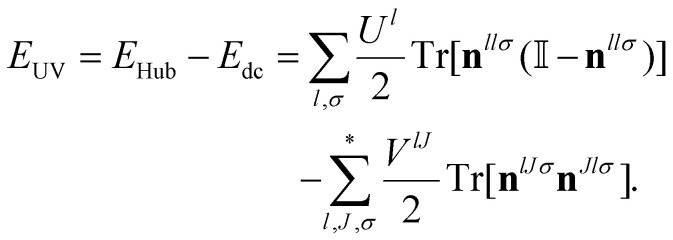
where the star over the sum denotes that the sum is taken over all the neighbours up to a given shell. The value *V*^*IJ*^ is the *V* Hubbard parameter between shells *I* and *J*. The Hubbard *V* favors states with localization in the neighbouring atoms, positively affecting hybridization that is usually suppressed by the exceed of localization introduced by the Hubbard *U* in the atomic centers.^104^

The DFT+*U*+*V* approach was used for NiO, Si and GaAs in its original publication.^87^ For NiO, it slightly improved the resulting bandgap and it greatly improved the description of the DOS. In the case of Si and GaAs, it improved the resulting values of different magnitudes, including the bandgap, with respect to DFT+*U*. The authors conclude that this approach performs better in Si and GaAs because they are more isotropic. Recall that the DFT+*U*+*V* assumes orbital independent electron–electron interactions and by construction it will perform better in highly isotropic systems.

The DFT+*U*+*V* approach is widely used^110,115–117^ and the obtention of *V* is fully automated thanks to the code **HP**^105^ which is part of QUANTUM ESPRESSO^114,118,119^ and its integration into the automation infrastructure AiiDA.^120–122^

Despite having ways to computes the Hubbard parameters, the approach has intrinsic limitations that could make it fail when describing some systems. The introduction of *V* parameter clearly illustrates how the approach can be generalized to consider more general regimes of localization, leaving then room for further improvements. Another possibility involves the implementation of the orbital-resolved Hubbard^123^*U* and *V* to more correctly describe the Hubbard manifold, thereby circumventing the limitations of the current shell-averaged approximation.

As a general conclusion, the self-consistent computation of the Hubbard parameters is a promising way to go, which recovers the predictive capabilities of DFT and results in an overall good modelling of the materials. However, a self-consistent Hubbard *U* does not always guarantee a good description of the properties of the system^124^ and thus, performing a complete analysis of various properties and external conditions provides a complementary methodology to determine the computational details of the DFT+*U* simulations.

Magnetic properties are heavily influenced by correlation effects^47^ and the overlap between electronic orbitals. The adequate description of these correlation effects can only be done with DFT+*U* methodologies and a good estimation of the Hubbard parameter. Additionally the *U* value affects electron localization. For all these reasons, there is a strong dependence of magnetic properties such as magnetic anisotropy energy (MAE) or exchange constants on the value of *U.*^25,30,47,124–126^ These magnetic properties determine the critical temperature of the material (as explained in section 4) and therefore, following this logic, the *U* parameter affects the final calculated critical temperature as well.

### 3.2. Improving the localization by using hybrid functionals

We already discussed when introducing DFT+*U* how common functionals tend to delocalize charge and suffer from the self-interaction error. A different approach to compensate the self-interaction error and to improve the performance of the functionals is by using the so called hybrid functionals.^41^ Hybrid functionals treat the exchange–correlation energy following:

28 *E*^hybr^_xc_ = *a*_0_*E*^exact^_x_ + (1 − *a*_0_)*E*^DFT^_x_ + *E*^DFT^_c_, 0 ≤ *a*_0_ ≤ 1.

where *E*^exact^_x_ is the exact exchange, *E*^DFT^_x_ is the exchange energy coming from a traditional DFT functional (LDA, GGA…) and *E*^DFT^_c_ is the correlation energy which comes from the DFT exchange–correlation functional. *a*_0_ is a parameter to be tuned to balance between the exact exchange contribution and the usual DFT exchange. The exact exchange energy is taken from a Hartree–Fock-like equation:29
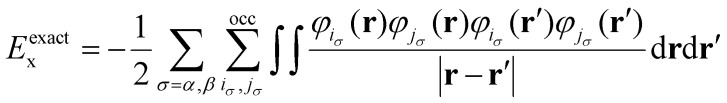
where *φ*_*iσ*_ are the Kohn–Sham eigenstates labelled by band index and spin. Therefore, hybrid functionals act over all electrons where as in DFT+*U*, the correction was applied only on those electrons considered as correlated or localized (usually d and f electrons). Moreover, DFT+*U* assumes an orbital independent value of the interaction energy among electrons with the same *l*-number. This assumption comes from the idea that localized states should retain atomic character and therefore, spherical symmetry.^86^ However this assumption can break down in cases where there is a mechanism capable of removing the energy degeneracy of those states such as situations with strong crystal field or strong SOC.^86,127^ On the other hand, hybrid functionals treat those electrons with the same *l* individually.

Hybrid functionals can alleviate some of the deficiencies of standard DFT with the caveat of a higher computational cost due to the computation of (29) and the need of tuning the a_0_ parameter. Hence, this approach removes the *ab initio* character of the simulation. This parameter is usually fitted semiempirically and its value is usually set around 0.2–0.3.^86^

It is important to remark that a higher amount of exact exchange (increasing the a_0_ parameter) does not necessarily imply a better result. This is because the exchange contribution from DFT, *E*^DFT^_x_ has also a contribution for the correlation, the so called nondynamic correlation (more details in ref. 41). Therefore, eliminating part of the correlation by setting *a*_0_ = 1 can be critical.

The mixing scheme of hybrid functionals can be extended even further by letting the mixing coefficient be spatially dependent:30
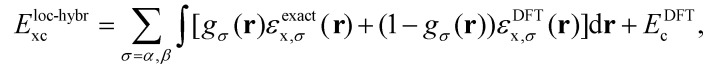
where *ε*^exact^_x,*σ*_(**r**) is the exact exchange–correlation energy density:31
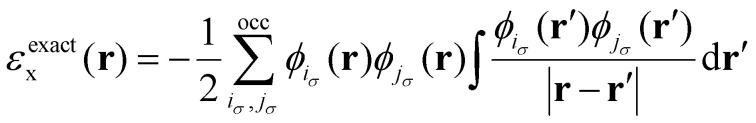
*ε*^DFT^_x,*σ*_(**r**) is the DFT exchange–correlation energy density and *g*_*σ*_(**r**) is the so called local mixing function (LMF), which follows 0 ≤ *g*_*σ*_(**r**) ≤ 1.

Hybrid functionals can help describing magnetic materials in a similar manner as DFT+*U*: improving the description of the electronic density by improving the description of the localization of the electrons. Hybrid functionals have shown to improve the results obtained with standard DFT functionals.^41,127–129^ Specially for systems with strongly correlated electrons in which the usual functionals fail. Unfortunately, they have the caveat that its usage introduces an additional input parameter in charge of balancing how much exchange is obtained from HF or from the usual functional. Additionally, the calculation of the exact exchange makes the usage way more expensive than with a standard functional.

### 3.3. van der Waals corrections in 2D magnetic materials

One of the issues of semilocal exchange–correlation functionals is their inability to account for long range correlation interactions such as London dispersion,^130,131^ resulting in an overrepulsive potential energy between atoms as a function of the interatomic distance.^130^ This interaction is one of the van der Waals type interaction and it can be of special relevance in condensed systems.

In order to compensate for this inaccuracy, several dispersion corrections for DFT exchange–correlation functionals have been designed in the last 20 years. Some of them are included in Table 1. These corrections calculate the dispersion correction in a semiclassical manner. In practice, the total energy computed within the self-consistent process is’:^131^

32 *E*_DFT+vdW_ = *E*_DFT_ + *E*_disp_

where the term on the left side is the total energy, *E*_DFT_ is the total energy obtained from standard DFT and *E*_disp_ is the energy correction term to account for the long range corrections. This energy contribution is calculated as:33
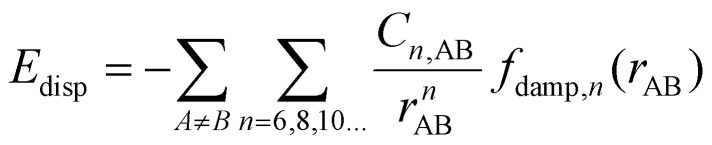
where the subindex AB means that the quantity refers to the atoms A and B. This way, the energy is the sum of potentials proportional to *r*_AB_^−*n*^ with dispersion coefficient *C*_*n*,AB_ and damping function *f*_damp,*n*_(*r*_AB_). The damping functions appear because every energy contribution applies only for long range. The damping function then makes sure that the contribution vanishes at short distances, avoiding potential overbinding effects.^130^Eqn (33) is obtained from the second order perturbation theory of the correlation energy after writing the Coulomb potential in a multipole expansion.^130^ This multipole expansion is the reason for the appearance of the sum 
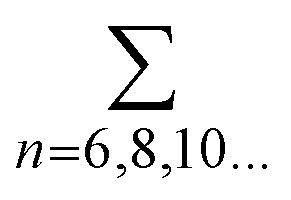
, being the term with *n* = 6 the one corresponding to the dipole–dipole interaction.

The details of the calculations of the terms *C*_*n*,AB_ are beyond the scope of this review, but we invite the interested reader to start with published reviews in the topic.^130,131^

In the particular case of 2D magnetic materials can be stacked to form bilayers or multilayers that are bonded by van der Waals forces. Additionally, the reduced dimensionality in monolayers makes subtle effects more important since they are not as easily quenched by other interactions as it happens in 3D materials. Therefore, when working with 2D magnetic materials, specially when working with more than one layer, it is a good practice to include any of these corrections to properly account for the long range correlations.

### 3.4. Obtaining the magnetic order of the ground state

Modelling a magnet within DFT is about finding its ground-state electronic density, but the electronic density and the spin order are not decoupled. The spin order influences the electronic density and structure. Therefore, a crucial step of the modelling process is focusing on finding the ground state magnetic order of the material under study.

**Fig. 4:**
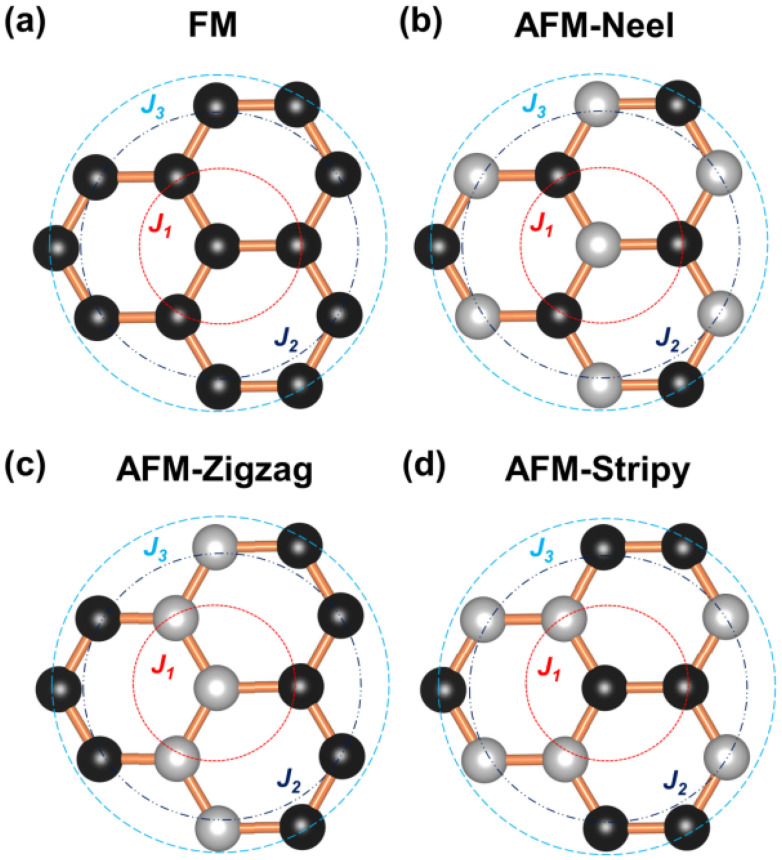
Sketch of the principal magnetic configurations considered in the exploration of the magnetic ground state in honeycomb materials. Black (white) spheres represent spin up (down). The illustrated configurations are: (a) ferromagnetic, (b) Néel antiferromagnetic, (c) zigzag antiferromagnetic, and (d) stripy antiferromagnetic. Figure extracted from ref. 133. Reprinted figure with permission from B. L. Chittari, Y. Park, D. Lee, M. Han, A. H. MacDonald, E. Hwang and J. Jung, *Phys. Rev. B*, 2016, **94**, 184428. Copyright 2025 by the American Physical Society.

In simple terms, finding the magnetic ground state is, in all cases, finding the spin arrangement on the lattice that minimizes the total energy of the system. The most widely used approach to find the magnetic ground state is the exploration of different magnetic configurations of the material, being the most frequently simulated magnetic configurations the FM and Néel AFM. However, a good practice is to extend this analysis to more complex arrangements of spins, such as the zigzag and stripy AFM (Fig. 4), that usually require the simulation of supercells. On the other hand, bulk materials demand the exploration of the FM/AFM coupling between adjacent layers. In addition to this conventional approach, the community has also explored alternative methods like crystal orbital Hamilton population (COHP) analysis to investigate the role of magnetic ordering in the structural stabilization of quasi-two-dimensional transition metal compounds.^132^

Some spin configurations such as spin-spirals or spin-canted might be difficult to simulate with a supercell approach in standard DFT due to the potential possible change in both the magnitude and the direction of the spins during the self-consistency algorithm. In those cases in which we need an special effort to maintain the desired spin arrangement, we can rely on spin-constrained DFT^134^ (sc-DFT) which allows to constrain the spins of the system, both in direction and magnitude. We highlight that one rule of thumb when initializing a spin arrangement is to always introduce as an input a value for the spin of the atom slightly higher to the one we expect. This is because DFT implementations tend to decrease this value during the process while the cases in which it increases are less common. So in summary, finding the ground state is about exploring all possible configurations to determine the one minimizing the energy.

The problem of this approach of exploring the landscape of possible spin arrangements is that the number of possible configurations is computationally unreachable,^7,13,30,135^ even when considering collinear configurations since the magnetic ground state can show a magnetic order with a periodicity that goes beyond the primitive unit cell, requiring the usage of supercells that can dramatically raise the cost of the calculation. This difficult downside can be partially solved by establishing a smart criteria to decide in advance which magnetic configurations are more likely to be the ground state while skipping the calculations of those configurations that are very unlikely to be the ground state. This idea resembles the idea of Bayesian optimization^111,112^ of balancing exploration and exploitation, being in this case exploitation the idea of skipping some configurations while progressing with those that seem to be close to the actual magnetic ground state.

This idea is put into practice in published workflows such as ref. 136 and 137. In ref. 136, a genetic evolution algorithm is designed in such a way only those configurations with low energy survive. The next generation of configurations is obtained from their ancestors, in such a way the new magnetic configurations to try will inherit partially the order of the parents. In principle, this would maintain the “likelihood” of a configuration to be the actual ground state. This workflow is named Magnene and it is designed to work for both collinear and noncollinear spin configurations.

In ref. 137, the workflow is designed only for collinear spin configurations. They set a ranking of most common experimentally found magnetic ground states and they set the likelihood of a new magnetic configuration based on this ranking. This way, the workflow starts calculating the most probable configurations leading to less time consumption most of the times.

With the guidelines given above, the only way to simulate spin-spirals would rely on a supercell approach and potentially using sc-DFT. This would make the calculations extremely expensive and potentially unreachable for long wavelength spin-spirals. Fortunately, there is an approach capable of considering these cases within a simple unit cell: the Generalized Bloch Theorem (GBT).^28,29,54,138–141^ This approach has one huge advantage and is the fact that it can model a spin spiral within a primitive unit cell. The GBT takes into account not only the translational symmetry of the lattice but also collective spin rotations over the same axis and along a given direction. The GBT extends the usual Bloch theorem stating that, considering the spin symmetry, the one electron wavefunctions can be written as:^140^34
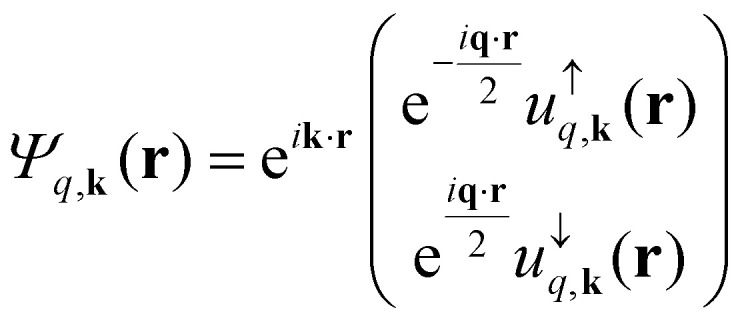
where **q** is the spin-spiral wavevector and **k** labels the state as done in the traditional BT. This result allows to simulate spin-spirals within the primitive cell. Its biggest caveat is that its derivation assumes that all directions are equivalent and therefore, the rotation axis can be set to the *z*-axis in such a way the spins behave as:35
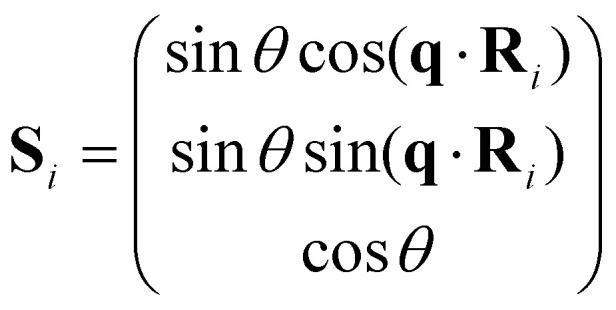
where *θ* is the cone angle (angle between the precession axis and the spin direction). The problem of this assumption is that important effects such as SOC can destroy the equivalency of directions and therefore the GBT is no longer a fully valid approach. At least, the effect of SOC can be incorporated perturbatively.^28,142,143^

Using the GBT produces a spin-spiral spectra along the high symmetry path of the BZ. The inspection of this energy spectra gives the wavevector **q** that minimizes the energy and consequently, with this vector we obtain the corresponding magnetic order that minimizes the energy for the given initial spin alignment.

It is important to remark that the GBT requires an initial spin alignment in the computational cell. Therefore, the GBT should be applied for several different initial spin alignments. Additionally, some collinear configurations can be regarded as limiting cases of spin spirals^28^ and therefore, the GBT can be applied to the study of collinear magnetic ground states as well.

The GBT+DFT has been already used in previous works such as ref. 28, 29, 144 and 145 where it is applied for several 2D materials and for 3D γ-iron in ref. 56 and 140. The GBT within DFT is already available in codes such as GPAW,^146–149^ VASP^150,151^ and FLEUR.^152,153^ The localized atomic orbitals DFT code OPENMX^154–156^ has also a GBT implementation.^157,158^

### 3.5. Magnetic ground states in 2D magnetic materials

**Fig. 5:**
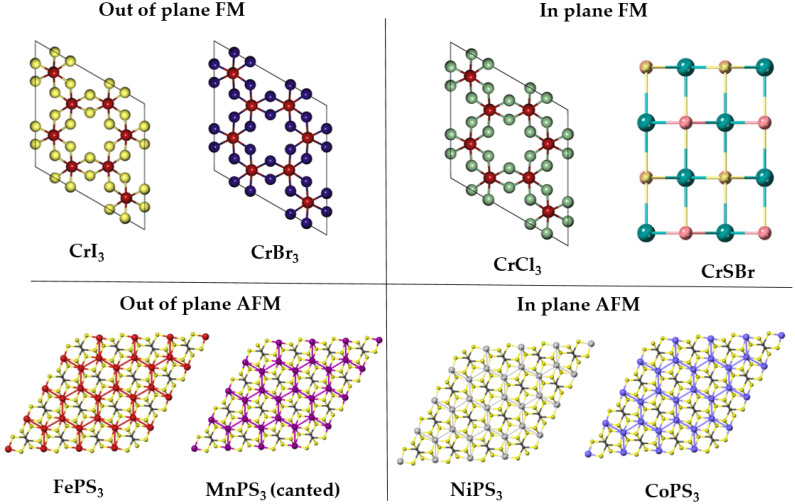
Classification of some important 2D materials in the monolayer limit.

2D magnetic materials can show a variety of magnetic orders in their ground state (see Fig. 5). In ref. 29, 192 magnetic materials from C2DB^22,23^ were studied with the GBT. 50 of them were found to be FM, 21 AFM, 34 commensurate non-collinear spin-spirals, 36 incommensurate spin-spirals and 15 chiral spin-spirals. This results exemplifies the many possibilities that can appear when trying to find the magnetic order of a 2D magnet.

Unfortunately, exploring that many spin configurations is an unreachable task. That is why it is common to see works that explore just a small number of collinear spin configurations and the ground state is then chosen among them. This approach can easily be implemented in high-throughput workflows such as^19,159^ that look for 2D ferromagnets from well established databases of materials.

When experimental information about the magnetic order is known beforehand, the reach of the magnetic order with DFT usually turns more into a verification rather than an exploration of all the possibilities. Instead of exploring multiple spin configurations until the energy minimum is found, one usually simply verifies that the experimentally obtained magnetic order is more stable than other similar magnetic configurations.

The realm of 2D materials provides a plethora of different magnetic scenarios, making this characteristic one of its most attractive features. There are many examples of 2D materials with the magnetic orders previously introduced, from the most basic to the most exotic. During a large part of the history of 2D magnetic materials, the scientific community has focused in the exploration of ferromagnetic materials such as the CrX_3_ (X = I, Cl, Br) or CrSBr monolayers. Other materials are well known by their intralayer-AFM such as the MPS_3_ (M: Mn, Fe, Co, Ni). Fe, Co and Ni compounds are examples of the zigzag AFM introduced in Fig. 4, by the other side MnPS_3_ presents an example of a Néel AFM. Also these materials provide a good example of different AFM (FePS_3_) and FM (CoPS_3_, NiPS_3_ and MnPS_3_) bulk phases. The exploration or verification of the magnetic orders from a simple collinear point of view, is typically applied in 2D materials, and tends to easily find agreement with the experimental findings.^133,160,161^

Noncollinear magnetic orders add more complexity to the determination of the ground state. The addition of SOC is usually enough to determine the noncollinear behaviour of a material. However, there are important exceptions such as the CrCl_3_ or CrSBr, where SOC is very weak and the in-plane nature of the spins is a consequence of the exchange and shape anisotropy and thus, the magnetic dipoles.

For example, the monolayer of CrI_3_ is an out of plane ferromagnet up to 45 K.^1^ The addition of SOC is able to verify the out of plane easy axis of this material and correctly estimate the difference in energy between the in-plane and out of plane spin alignment.^6^ In contrast, the ground state magnetic order of monolayer CrSBr is described as an in-plane ferromagnet in the presence of SOC.^51,162^ The stability hierarchy between the different possible in-plane spin directions agrees with the experimental results^163^ only when shape anisotropy is considered.^160^

More complex ground states are represented by the spin-spirals, where the spins are not aligned uniformly, but instead, they rotate continuously in space, originating helical patterns in the spin orientation across the material. Spin spirals are a manifestation of complex magnetic interactions and are often found in 2D materials with competing magnetic exchange interactions or broken inversion symmetry. Particularly well studied cases of spirals in 2D materials are the metal dihalides (MX_2_) such as the Ni or Co compounds.

**Fig. 6:**
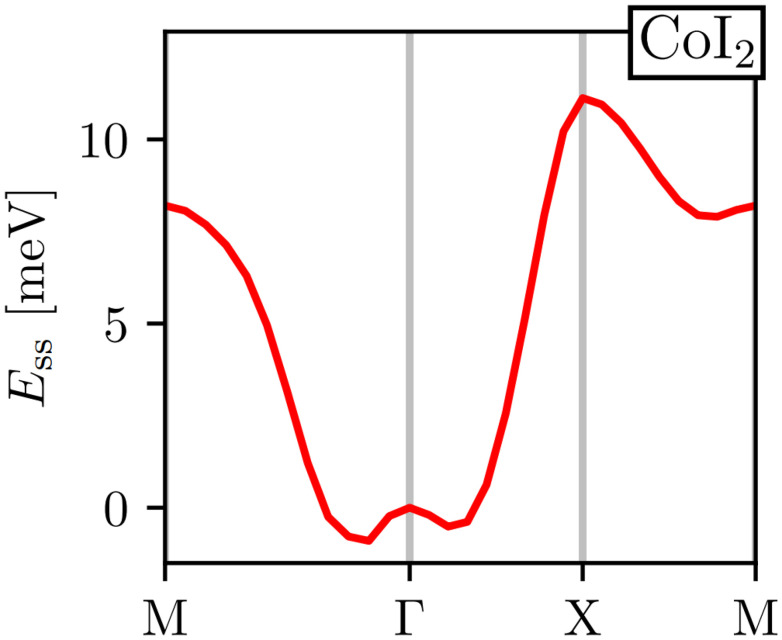
Spin-spiral spectra of CoI_2_. Figure extracted from ref. 29 without changes under Creative Commons Attribution 4.0 International License https://creativecommons.org/licenses/by/4.0/.

In Fig. 6 we show one of the results of ref. 29 for CoI_2_. The figure shows the energy spectra obtained after doing DFT calculations with the generalized Bloch theorem. This material presents its energy minima between the M and Γ points. Therefore, under the approximations of the GBT, its magnetic ground state is a non-collinear spin-spiral. Ideally, Fig. 6 should be repeated for different initial spin alignments within the computation unit cell. However, that would require even more exploration of configurations and therefore, more time and resources. It is always necessary then to stop the exploration of more configurations based on the quality of the results and commit to what you already have.

Other 2D materials present an strong itinerant magnetism, which complicates their modelling and simulation, such as Fe_3_GeTe_2_ and Fe_3_GaTe_2_. These materials have attracted the attention of the community, given their very high critical temperature.^3,164,165^

In conclusion, finding the magnetic order of 2D magnets with DFT involves exploring several different configurations *i.e.* a lot of time and computational effort. However, in practice, experimental knowledge or simply common sense limit that huge exploration to simply the calculation of a small set of spin arrangements from which the magnetic order will be chosen.

### 3.6. What DFT codes can I use?

There are many DFT codes available and not all of them offer the same relevant features in the context of magnetism. That is why we summarize the availability of some of the features we discuss in this review in Table 1 for some of the most known and widespread DFT packages. These features have been compiled by inspection of the manuals of the corresponding codes.

## 4. Obtaining exchange parameters

Once the magnetic ground state is known, the parameters of an atomistic magnetic Hamiltonian can be obtained from DFT. In this review, we will describe in simple terms the usual approaches to do so.

### 4.1. Energy mapping method

The energy mapping method is the most popular technique to calculate exchange interactions.^6,14,16,17,30,204–207^ The fundamental approximation behind this method consists in dividing the contributions to the total energy into two components:36
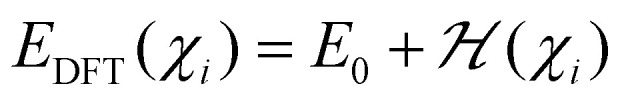
where *E*_0_ is just a reference energy which is assumed to be independent of the spin configuration, *χ*_*i*_ denotes a spin configuration and 
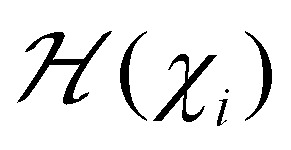
 corresponds to the magnetic spin-dependent Hamiltonian for that specific spin configuration. It is obvious that this assumption is quite strong: a change in the spin configuration can also change the energy contributions of the bands, for example, and hence, the decoupling in (36) is very naive. Nevertheless, the assumption works well for insulators and semiconductors.

Now, consider a different spin configuration: *χ*_*j*_. Then: 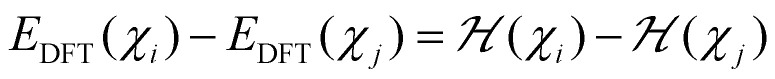 and since the spin configuration is known beforehand, the last equality can be expressed in terms of the Hamiltonian parameters. With this approach, for a Hamiltonian with *n* parameters, one needs *n* energy differences *i.e. n* + 1 DFT results. This way, we create a system of *n* equations with *n* variables that we can solve. Solving the system will give the expression of the magnetic parameters. In practice, the final expressions depend on the type of spin lattice under consideration and therefore they are geometry-dependent. Most of the times, it will be necessary to use a bigger computational unit cell in order to capture more exchange interactions such as nearest or second nearest neighbours depending on the specific material. This is one big caveat since it can increase a lot the computational cost of the method. This method can be applied for both collinear^30^ and noncollinear configurations.^47^

The important limitation of this method is that it frequently requires to construct supercells to consider the different magnetic configurations, a task that strongly affects the computational efficiency of this method. In different materials, the computation of supercells imposes limitations in the convergence threshold to converge the charge density, compromising both the convergence effort and the quality of the calculations. Moreover, this method importantly relies on the extraction of *n* + 1 different energies, and often configurations can reach apparently correct local minima with importantly wrong energies, seriously affecting the extraction of the sensitive meV energies of the exchange interactions. Last but not least, in order to achieve meV–μeV accuracy, the convergence parameters required are usually very large. In conjunction with SOC to capture anisotropy effects, the calculation becomes very expensive. In the end, obtaining exchange parameters by total energies analysis requires both a vast amount of computational resources and a good control over the inputs and parameters so that the DFT calculations can achieve the necessary accuracy.

It is important to remark that this method has the implicit assumption that all spin configurations are eigenstates of the magnetic Hamiltonian 
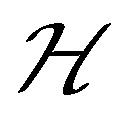
. This way, the energy from DFT for a certain spin configuration is mapped to an eigenstate of the same spin symmetry. This puts a significant constraint on the magnetic configurations that can be run in DFT. Hence, most of the times the spins in 
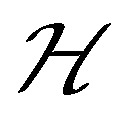
 are treated as classical vectors so that all spin configurations are regarded as eigenstates of 
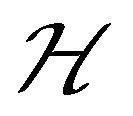
. This approximation works best when quantum effects are not important *i.e.* for high values of *S* and/or high temperatures. This issue has been discussed already in ref. 208. In this work, they highlight that the AFM configuration is not an eigenstate of the Heisenberg Hamiltonian and therefore, it should not be considered for rigorous energy mapping purposes. Instead, one should use the non-interacting magnon state (NIM).^32^ Considering this state and the FM state for the energy mapping, the resulting expression of the exchange parameter differs from the one considering FM and AFM states:37
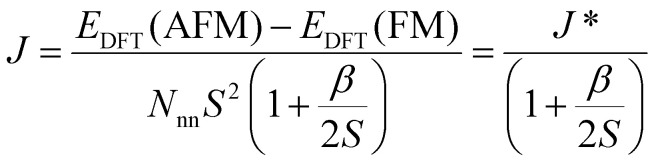
where *S* is the value of the spin (only one magnetic ion per unit cell was considered) J* is the exchange constant considering the FM and AFM configurations and *β* is a factor depending on the type of latticem, *β* ≥ 0. Hence, it is clear that the bigger the value of *S*, the smaller the correction is. Nevertheless, the exact expression of the parameter will change between different cases.

Another relevant remark in ref. 208 is that the value of *β* is bigger in 2D than in 3D materials. This hints that the accuracy of the energy mapping would likely be worse in 2D materials.

### 4.2. Fitting the spin-spiral spectra

Another way to calculate the exchange parameters is by fitting the spin-spiral energy spectra obtained after using the GBT. This approach is perfectly illustrated in ref. 144, 145 and 209. By inserting eqn (35) in the magnetic Hamiltonian, an expression of the energy as a function of **q** can be obtained. Then, the spin-spiral spectra can be fitted with the parameters of the atomistic magnetic Hamiltonian.

One relevant remark is that since the GBT does not consider SOC explicitly but perturbatively, the fitting of the spin-spiral spectra must be done only with those interactions that are present without SOC. For those that arise with SOC such as SIA or DMI,^37^ a similar approach can be done but with the spectra that contains only the perturbative contribution to the total energy.

One advantage of this method is that it allows to verify if all the interactions included in the magnetic Hamiltonian are enough to describe the system. If the resulting fit is not capable of describing some features of the spectra, it is likely that one necessary interaction has not been considered. The main disadvantages are those of using the GBT.

### 4.3. Approaches based on the magnetic force theorem and the LKAG approach

The approaches that can circumvent the problems of the previous methods are those based on the so called LKAG (Liechtenstein, Katsnelson, Antropov and Gubanov) approach and the magnetic force theorem.^210,211^

In 1987, LKAG obtained the Heisenberg exchange constants by considering small changes in the total energy of the system due to small perturbations of the spins of the ground state.^31^ When the perturbation is small enough, the energy variation can be calculated using the so called magnetic force theorem:^31^38
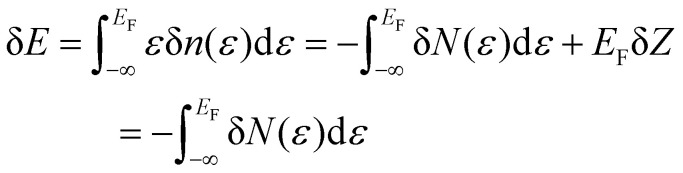
where 
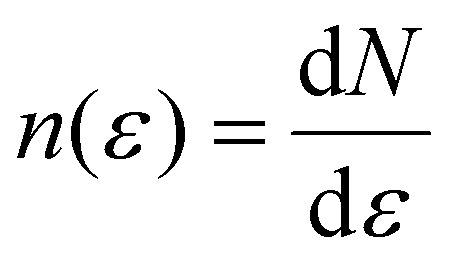
 is the density of states of the single-particle states (in our case, the Kohn–Sham states), *E*_F_ is the Fermi energy and δ*Z* is the total number of electrons, which is equal to zero for changes in the spin configuration. The intuitive idea of the approach is to write the left-hand side of the equation in terms of the magnetic Hamiltonian parameters when considering two infinitesimal spin variations at sites *i* and *j i.e.* δ*E*_*ij*_. The right hand side of the equation can be expanded in terms of Green function's^212–214^ of a Hamiltonian obtained in the last iteration of the DFT calculation. The spin rotations can be introduced in this Hamiltonian by rotating some of its parts^215^ and then, expressions for the exchange parameters can be deduced.^214^ This rotation can be done easily only if the basis sets used in the DFT calculation is of localized character. This is why practical implementations usually require LCAO calculations or a Wannierization although a plane-waves implementation of the magnetic force theorem exists.^216^ The LKAG to this day has been used to compute symmetric exchange,^31,217^ anisotropic exchange,^214^ DMI interaction,^213,218^ biquadratic exchange^219^ … The formalism behind this approach is too complex to be introduced here but the interested reader can find more information in extensive reviews.^210,211^

#### 4.3.1. Available codes implementing LKAG methods

Probably, the most famous package to calculate exchange constants is TB2J.^214^ This package takes Wannier90^220^ functions or LCAO DFT results to construct a tight-binding Hamiltonian in order to calculate interactions such as isotropic exchange, anisotropic exchange and DMI. TB2J can use directly the results of Siesta and OPENMX, while for plane waves or FLAPW codes, localized functions have to be constructed *via* Wannier90.

Jx is another code based on the same LKAG and magnetic force theorem approach.^221^ However, the code calculates only the symmetric exchange. Jx is compatible with any output of Wannier90 and directly compatible with OpenMX.

Last but not least, we have Nojij,^222^ capable of calculating the symmetric exchange from Siesta calculations. Nojij is being extended to a package named Grogu^223^ which is capable of calculating symmetric exchange and SIA. Unfortunately, to our best knowledge, Grogu is not yet publicly available.

### 4.4. Exchange mechanisms driving magnetic order in 2D magnets

As we have explained before, the long range magnetic order in 2D magnets with infinite system size has to be stabilized by anisotropy. The stabilization can be done mainly by two different means: single ion anisotropy or anisotropic exchange. For example, CrI_3_ is an out of plane ferromagnet, but its magnetic order is stabilized by two-ion anisotropic exchange along the *z* direction since the Cr atoms SIA is very small.^6,25^ The same behaviour has been obtained^25,224^ for CrBr_3_. Contrary to these cases, the in-plane ferromagnetism of monolayer CrSBr is stabilized by SIA since its anisotropic exchange results negligible from DFT calculations.^99^ However, SIA is not the only interaction driving its magnetic order since dipolar interactions have been proved to affect it.^51^

One of the most important steps when modeling the magnetism of the material is the selection of the atomistic Hamiltonian and the included interactions. All the examples in the paragraph above were capable of giving such a prediction because those interactions were included in their model magnetic Hamiltonian. It is common to see in literature how certain interactions are assumed to vanish. For example, there are many studies in which the two-ion anisotropy is assumed to be zero along the *x* and *y* directions. For obvious reasons, this assumption is incompatible with the potential prediction of an in plane ferromagnetism driven by anisotropic exchange. Analogously, the SIA is usually taken to be along the *z* axis *i.e.* out of plane direction. However, this assumption can be wrong, and the clear example is monolayer CrSBr which has an in-plane easy axis and in-plane ferromagnetism driven by SIA.

In conclusion, choosing an adequate amount of parameters to include in the model is equally important as their calculation. As the widespread quote says: “you can only be as good as your model”.

## 5. Calculation of the Curie temperature

Several methods exist for calculating the critical temperature of a material based on a known magnetic Hamiltonian, each with varying levels of accuracy, applicable regimes, and distinct advantages and limitations. A comprehensive understanding of these methods is valuable for interpreting results effectively and, where appropriate, for combining techniques to improve accuracy or achieve rapid estimations of the critical temperature. In this section, we will introduce the most famous ones for ferromagnetic materials (although most of them can be extended for antiferromagnets as well), highlighting their limitations and how they can be use in synergy with others.

### 5.1. Using mean field theory

Mean Field Theory (MFT) for ferromagnetism^32,225^ introduces the concept of an effective, uniform magnetic field acting on all ions in a lattice, referred to as the molecular field. This molecular field is assumed to be directly proportional to the magnetization of the system, expressed as 
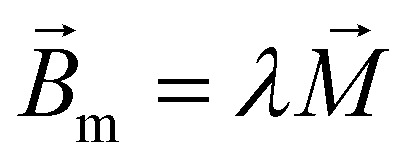
, where λ is a proportionality constant. In MFT, this molecular field affects all ions uniformly, allowing the ferromagnetic system to be treated as a paramagnet subject to an internal magnetic field equivalent to the molecular field.^36,225^ The primary function of the molecular field is to align all spins parallel to it, effectively substituting the ferromagnet's exchange coupling. For the model to be self-consistent, λ must be positive.

In a lattice of identical magnetic ions, the critical temperature with MFT is as follows:^226^39
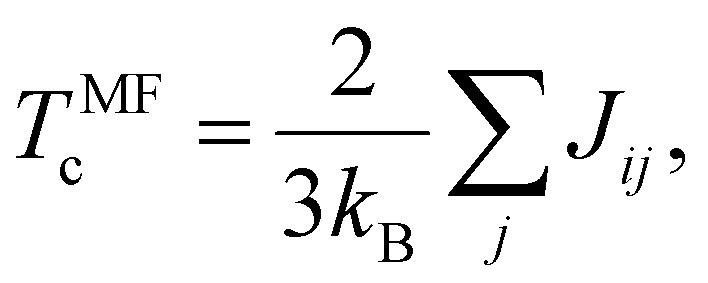
where 
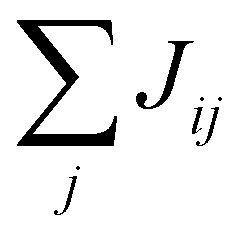
 represents the sum of exchange constants between nearest neighbors. Some sources, such as,^15,32^ include an additional factor, *S*(*S* + 1), to account for the spin of the ions. This factor is omitted here since, in our convention, the exchange constants are expressed in energy units, and spins are treated as unit vectors.

In two-dimensional materials, the presence of magnetic order necessitates anisotropy. However, eqn (39) does not account for either anisotropy or dimensional effects, rendering it inapplicable for precise critical temperature calculations in low-dimensional systems. Nevertheless, this equation can still serve as a rough estimate or an upper bound for the magnetization. Studies indicate that eqn (39) tends to yield a critical temperature roughly twice that obtained from Metropolis Monte Carlo simulations.^5,14^ Such simulations generally require an as input parameter the maximum temperature to be considered. For this use case, eqn (39) provides a convenient and systematic method to establish a maximum temperature to be simulated in Monte Carlo simulation or equivalently, an upper bound for the critical temperature.

### 5.2. The formulas for the 2D lattices with the Ising model

Consider the Ising model with no anisotropy and only nearest-neighbor exchange interactions. This model depends solely on the exchange parameter *J*, assumed constant across all nearest neighbors, and on the number of nearest neighbors, which is determined by the lattice. Consequently, the critical temperature in this model is a function only of the lattice type and the value of *J*. The relationship between critical temperature, lattice structure, and *J* has been investigated both numerically^227^ and analytically.^228^Table 2 provides critical temperatures for common lattice types.

**Table 2:** Relation between the critical temperature and the *J* exchange constant with the Ising model for several lattices

Lattice	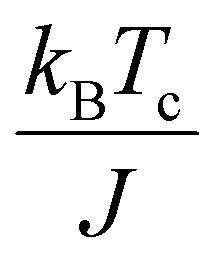
Square	2.2692
Hexagonal	1.5186
Honeycomb	3.6410

The Mermin–Wagner theorem^4^ implies that magnetic anisotropy is required for long-range magnetic order in two-dimensional materials with infinite system size. The Ising model, however, implicitly assumes an infinitely strong single-ion anisotropy, which can lead to an overestimation of the critical temperature in two-dimensional systems, as noted in previous studies.^15,205,229^ For instance, in ref. 205, the calculated Ising model critical temperature for both bulk and monolayer CrI_3_ was found to exceed experimental values significantly – more than twice the experimental value for monolayer CrI_3_ and nearly four times that of the bulk material. Similarly, ref. 230 reported a critical temperature of 161 K for CrI_3_ using the Ising model, far above the experimentally observed 45 K.^1^

Despite these discrepancies, the Ising model's critical temperature remains a practical upper bound when the lattice structure is one of the studied cases, and the exchange parameters are known. This upper bound is useful for setting a maximum temperature in simulations of magnetization *versus* temperature, such as Metropolis Monte Carlo methods, providing a systematic approach for simulation limits.

### 5.3. Methods based on calculating the magnetization with temperature

It is well established that ferromagnets exhibit zero magnetization at and above the critical temperature. Consequently, the critical temperature can be estimated by analyzing the behavior of the system's magnetization as a function of temperature. In this section, we outline various theoretical approaches and methodologies for calculating the magnetization–temperature relationship, spanning multiple levels of theory. In the following section, we will discuss methods for fitting the resulting data to extract precise estimates of the critical temperature.

#### 5.3.1. Using spin-wave theory

Spin-wave theory studies magnons, quasiparticles that describe the collective motion of the spins in a wave-like behaviour. Magnons can appear as thermal excitations, and they are capable of breaking the long range magnetic order in magnetic materials. Hence, studying their population in the material can lead to the value of the critical temperature. One important remark is that in this section, the spins will be considered as quantum operators instead of unit vectors.

In spin-wave theory, the traditional spin operators in the atomistic magnetic Hamiltonian are substituted by the so called Holstein–Primakoff (HP) spin operators. These are:^231,232^40
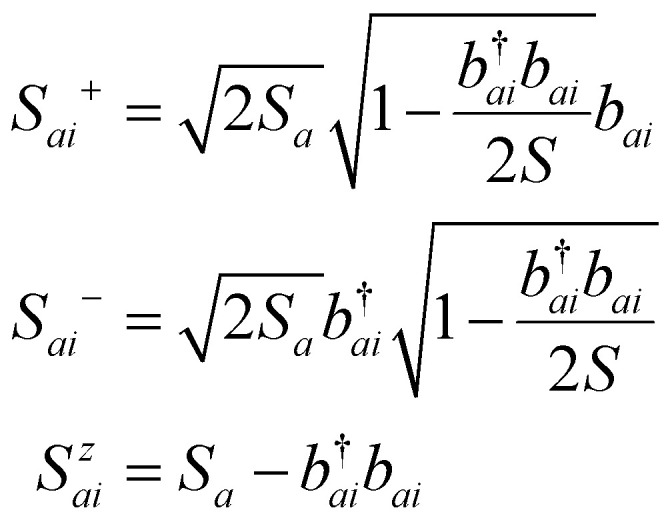
where the subindex *ai* represents the magnetic ion *a* in the unit cell *i* and *b*^†^_*ai*_ (*b*_*ai*_) are the bosonic creation (annihilation) operators. This representation is the most common in the literature although there are some others that serve the same purpose such as the Dyson–Maleev,^233–235^ the finite differences^236^ representation^237^ or the differential equations approach in ref. 238. This representation can also be generalized to antiferromagnets.^32^

The physical intuition of these operators is that the spin state |*S*,*m*_*S*_ = *S* 〉 is mapped to the vacuum bosonic state. Then, the states with lower *m*_*S*_ are obtained as excitations of the vacuum bosonic state *i.e.* acting with *b*^†^_*i*_ creates a boson and decreases *m*_*S*_ by one. As a consequence of the (2*S* + 1) possible values of *m*_*S*_, there can be at most 2*S* bosons when considering a single magnetic ion. The appearance of the square root term has two functions. The first one is to maintain the commutation relations of the spin operators *i.e.* [*S*^+^*S*^−^] = 2*S*^*z*^ and [*S*^*z*^*S*^±^] = ±*S*^±^. The second one, to keep the number of bosons to be 2*S* at most. Consider the state |*S*,*m*_*S*_ = −*S*〉. In the bosonic picture, this state corresponds to |2*S*〉_B_*i.e.* 2*S* bosons. If we want to add one more boson, equivalently, applying the *S*^−^ operator, we get *S*^−^|2*S*〉_B_ = 0 since the term in the square root 
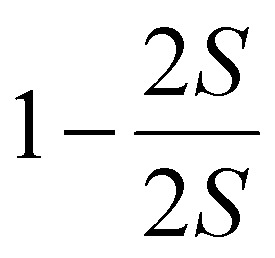
 vanishes. This way, the number of possible bosonic states is kept to (2*S* + 1) and the map between the spin states and the bosonic states is biyective. With this approach, the Holstein–Primakoff operators are equal to the traditional spin operators in the sense that they have the same matrix elements.

In the bosonic picture we have described before, the bosons are interpreted as magnons which break the magnetic order.

In practice, these operators are substituted in the desired magnetic Hamiltonian and then, expanded with their first order Taylor term:41
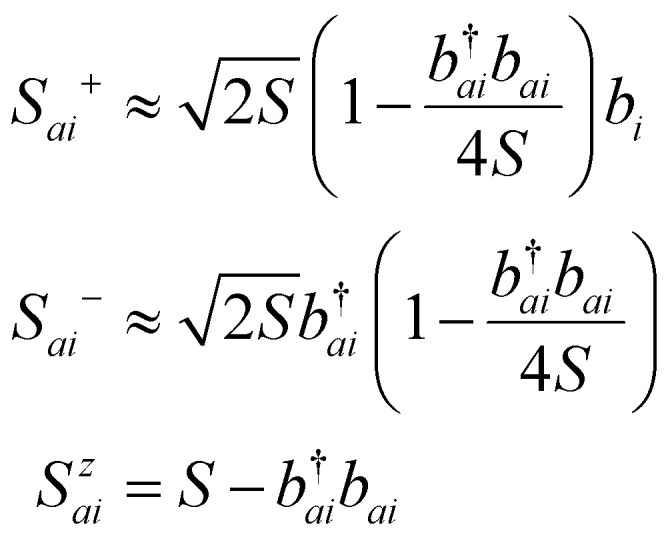


Operating this expansion leads to a sum of products of bosonic terms times powers of 
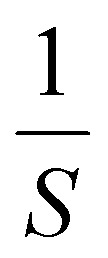
. Products of four or more bosonic operators which correspond to magnon interactions are usually neglected. This is called linear spin-wave theory because only the linear terms are included. This approximation is reasonable at low temperatures, when these interactions are not dominant^6,15,52^ or at high values of *S*, where the multiplying factor 
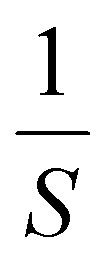
 makes these contributions negligible. When interactions are included, the process is referred as nonlinear spin-wave theory. The main problem of nonlinear spin-wave theory is that despite including interactions, usually they have to be treated in a Hartree–Fock-like or mean field manner^6,52,232,239^ and the final results end up being equally inaccurate at high temperatures as shown in ref. 15 for both the linear and nonlinear case.

Either approach and the usage of Bloch's theorem^240^ leads to the energy dispersion relation *E*(**k**) that can be used to count the total number of magnons. Using linear spin-wave theory on ferromagnets leads to a dispersion relation for low |**k**| as:

42 *E*(**k**) ≈ *Δ*_0_ + *ρk*^2^

In this context, *Δ*_0_ denotes the spin-wave gap, while *ρ* represents the spin-wave stiffness. The spin-wave gap *Δ*_0_ depends on both single-ion anisotropy and exchange anisotropy.^6,15,241^ However, the precise functional form of this dependence is determined by the specific magnetic Hamiltonian employed. This dependency has a physically intuitive basis: in the absence of anisotropy, the system exhibits isotropy in spin orientation, implying that all spin directions are energetically equivalent. Consequently, it is possible to rotate all spins in the system without altering the total energy. Under these conditions, a spin may be excited with minimal energy to deviate from its initial state, thereby enabling the generation of magnons at very low energies. In two dimensions, this facilitates the destabilization of magnetic order due to low-energy magnon excitations. This interpretation is consistent with the fact that the critical temperature increases with increasing spin-wave bandgap.^6^

Nonlinear spin-wave approaches lead to an energy dispersion that depends on the total number of magnons:^15,32,225,232^

43 *E*(**k**) = *E*(**k**;*N*_magnons_)

But since magnons are spin 1 bosons, the obey Bose–Einstein statistics and the total number follows the Bose–Einstein distribution:^15,232^44
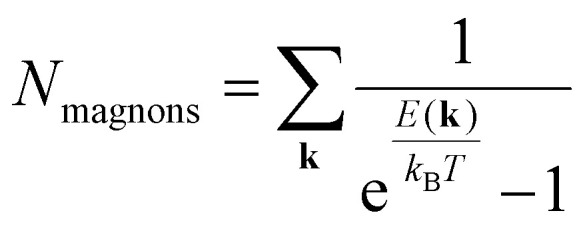
where the sum runs over all the **k** points in the first Brillouin zone. For a small enough spacing between **k** states, we can approximate the first Brillouin zone by a continuum. By doing so, the number of magnons is:^32,225^45
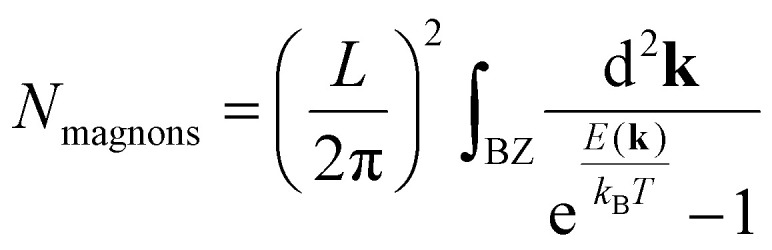
where the prefactor 
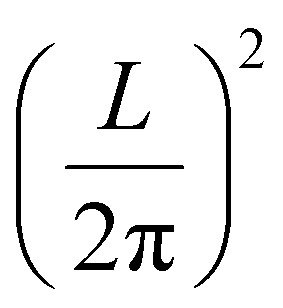
 is to account for the density of **k** states *i.e.* every **k** state occupies an area of 
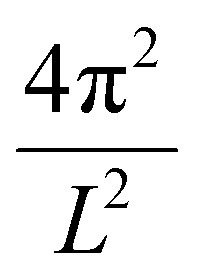
 in the reciprocal space. So in the end, the total number of magnons depends simultaneously on the energy dispersion. This forces the need of solving *E*(**k**) and (44) or (45) in a self-consistent manner when nonlinear spin-wave theory is used.

Since magnons are spin 1 bosons, we can calculate the total magnetization (assuming a single magnetic ion per unit cell) as:46
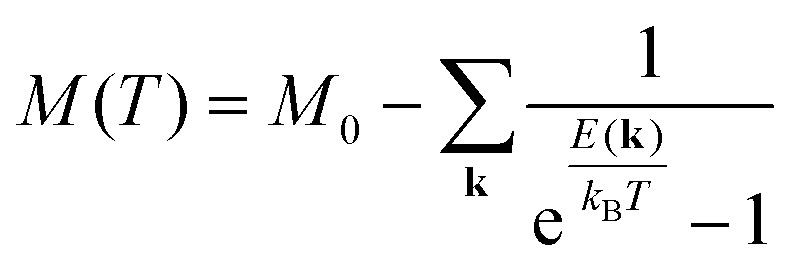
where *M*_0_ is the saturation magnetization of the system. This expression must be consequently modified when more than one magnetic atom is considered in the unit cell. The analogous expression for a continuous approximation is:47
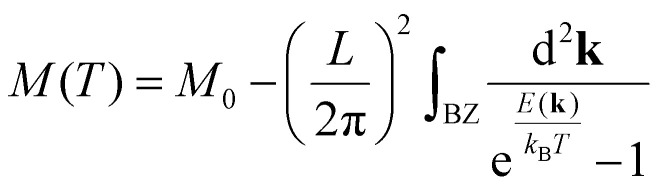


And in the end, obtaining the magnetization with temperature is about solving (46) or (47) numerically as a function of temperature. When nonlinear spin-wave theory is applied, the solution of (43) and (46) or (47) must be done self-consistently. Afterwards, the critical temperature can be detected by inspecting where the magnetization or the spin-wave gap vanish.

Even though spin-wave theory fails to describe properties at high temperatures, in particular the critical temperature, we include it here for the sake of completeness and because its accuracy at low temperatures can be very useful when aiming to calculate magnitudes that depend on the magnetization and its derivatives.

##### 5.3.1.1. Alternative representations to the Holstein–Primakoff representation

In the non-linear spin-wave theory approach, the ladder operators were expanded using the Taylor expansion of the square root (41). The expansion was truncated, keeping terms with up to four bosonic operators that were approximated afterwards in a Hartree–Fock like approach. This truncation leads to neglecting magnon–magnon interactions.

However, as noted in ref. 237, this truncation also breaks the commutation relations of the spin operators and eliminates the restriction *S*^−^|2*S*〉_B_ = 0 since now *S*^−^ is just a truncation of what it originally was. Hence, after the truncation, one can get bosonic states with more than 2*S* bosons, and those states are unphysical.

In an attempt to tackle this issue, in ref. 237 they propose to expand the square root with Newton finite differences^236^ instead of a Taylor expansion. They show that the square root can be written as:48
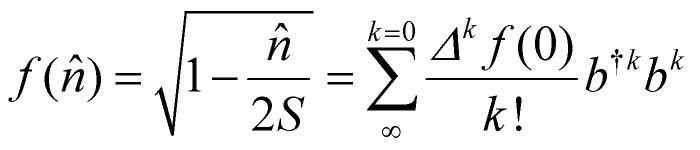
where *n̂* = *b*^†^*b* is the number operator, Δ*f*(*x*) = *f*(*x* + 1) − *f*(*x*) and *Δ*^*k*^ = (*Δ*)^*k*^. Consider an *r* order expansion of (48) and let us call *S*_*r*_^+^ and *S*_*r*_^−^ the resulting expansions of the ladder operators. The authors found that the resulting commutation relations are:49
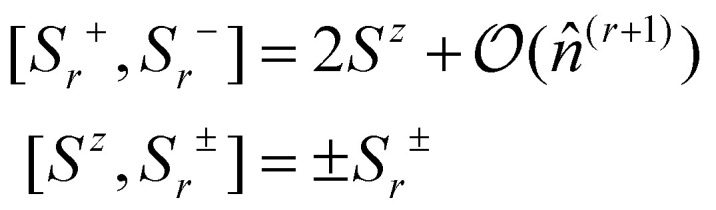
where *n̂*^(*k*)^ = *n̂*(*n̂* − 1)…(*n̂* − *k* + 1). Note that *n̂*^(*r*+1)^ vanishes unless there are at least *r* + 1 bosons.^237^ Hence, the first commutation relation in (49) yields the correct result when 2*S* ≤ *r* and with that condition, the operators given by an order *r* truncation respect the original spin commutation relations. An order *r* truncation of the Taylor expansion yields 
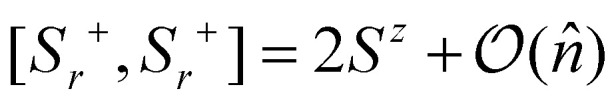
 and hence, the commutation relation does not improve when *r* increases, contrarily to the finited differences expansion.^237^

Recall that in the context of the HP operators, a magnetic ion of spin *S* can have at most 2*S* magnons. Then the results in (49) proof that truncating (48) for *r* = 2*S* and plugging it into the HP operators in (40) gives a set of spin operators that follow exactly the traditional commutation relations and are equal to the spin operators matrix-element-wise. Therefore, this would be an exact representation.

The presented advantages should lead to an improvement when carrying out spin-wave theory with this finite differences representation. However, to our best knowledge, there is no quantitative comparison with respect to the usual Taylor expansion approach.

There have been other approaches towards the obtention of an exact spin representation up to a certain number of bosons as done in ref. 238 by using a set of differential equations. The interested reader can find more information in ref. 238 and the references therein.

#### 5.3.2. Using Metropolis Montecarlo (MMC)

Consider any of the magnetic Hamiltonians discussed in this review. For any lattice, the number of possible collinear spin microstates for the system is 2^*N*^, where *N* is the number of magnetic ions in the lattice. Therefore, if we aim to find a configuration that minimizes the energy, examining each configuration individually becomes infeasible.

The Metropolis Monte Carlo algorithm^33,242,243^ (MMC) addresses this challenge by following a systematic criterion to explore various spin configurations of the system. First, assume that all spins behave as classical particles, a premise whose validity will be discussed later. The probability of finding the system in a microstate *k* with energy *ε*_*k*_ is given by Boltzmann statistics:50
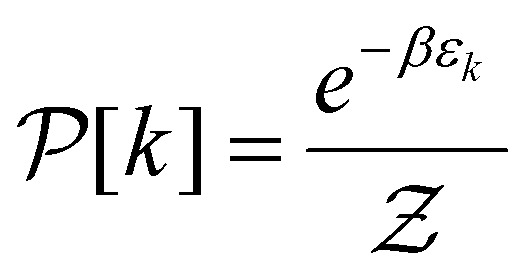
where the sum runs over all microstates with energy *ε*. Here, 
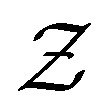
 is the partition function of the system:51
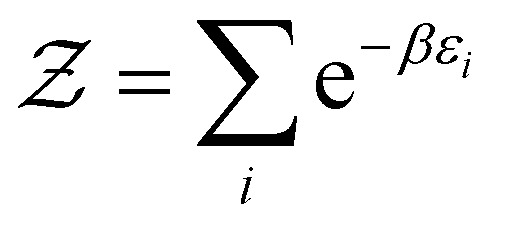
where the index *i* runs over all possible microstates of the system. Thus, computing the partition function and 
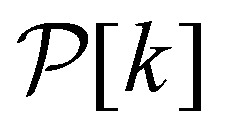
 is impractical for real systems. However, we can still determine whether one configuration is more probable than another by calculating the ratio:52
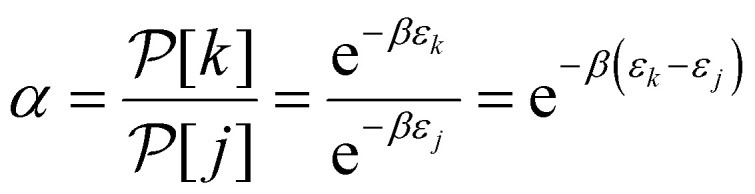


When this ratio is greater than one, it follows that 
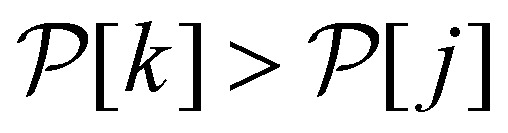
, or equivalently, *ε*_*k*_ < *ε*_*j*_. The Metropolis algorithm thus attempts to find the system's ground state by systematically sweeping through spin configurations and evaluating which microstate is more probable by repeatedly calculating the ratio in (52). The full algorithm proceeds as follows:

1. Initialize the system in a specific microstate, *e.g.*, a ferromagnetic configuration.

2. Select one spin of the system and rotate it.

3. Calculate the ratio (52)*α*. Choose a random number *r* in (0,1).

4. If *n* > *r*, update the microstate of the system.

5. Repeat steps 2–4 until the system stabilizes.

6. Calculate desired quantities: total energy, magnetization, *etc*.

Note that step 4 is designed such that some less probable configurations can still be accepted. The optimal value for this acceptance rate is 0.5.^33,242^ The acceptance rate is controlled by the algorithm that rotates the spins. A naive approach would involve simply performing a 180-degree flip, resulting in an Ising-like simulation. The Ising model implicitly assumes infinite single-ion anisotropy (SIA). Since anisotropy is responsible for long-range magnetic order in two dimensions, this approach would lead to an overestimation of the critical temperature.^205^ This can be confirmed with the examples shown in Table 3.

**Table 3:** Critical temperatures of chromium based 2D ferromagnets obtained with Ising based Monte Carlo *vs.* experiment

Material	*T*_c_Ising__/K	*T*_c_ (experimental)/K
CrI_3_	107^244^	45^245^
CrBr_3_	86^244^	27,^245^ 34^246^
CrCl_3_	66^244^	16^245^ (bilayer)

The specific algorithm used to rotate the spins significantly impacts the efficiency and accuracy of Monte Carlo simulations. Various methods have been developed to rotate spins continuously, aiming to achieve an optimal acceptance rate of approximately 0.5.^247^ For a detailed discussion on these approaches, see ref. 243, 247 and 248.

It is essential to note that this method relies on the assumption that spins behave as classical particles. This classical approximation becomes invalid at low temperatures, where quantum effects play a significant role.^205,249^ Consequently, the Metropolis Monte Carlo approach may yield inaccurate results for magnetization values at temperatures near absolute zero or far from the critical point. This limitation should be considered when calculating temperature- and magnetization-dependent properties.

As temperature increases, thermal fluctuations dominate, effectively reducing the significance of quantum effects. At sufficiently high temperatures, the classical approximation becomes appropriate, allowing the Metropolis algorithm to perform well in estimating critical temperatures and magnetization.^14^ The primary limitation of this method lies in its computational expense, which can become significant for large or complex systems.

One last important aspect to consider is the fact that MMC takes as an input a finite size system and the Mermin–Wagner theorem applies only to infinite size system. Therefore, the convergence of the MMC result as a function of the system size must always be taken into account to analyze finite size effects. This has been studied in ref. 250, where they showed that finite size effects can even stabilize 2D magnetism in absence of anisotropy.

##### 5.3.2.1. Empirical rescaling of the temperature

As explained previously, the MMC method yields accurate values for the magnetization for high and close to *T*_c_ temperatures. The values it provides for the magnetization at low temperatures are not accurate and this can turn into an obstacle when calculating quantities that depend on the magnetization or its derivatives. Evans *et al.* managed to solve this issue with a simple rescaling of the temperature of the MMC.^249^ Since MMC considers spins as classical particles, the real temperature we would need to obtain the same results (making quantum effects negligible) in a laboratory should be higher. The quantitative relation they suggest is:53
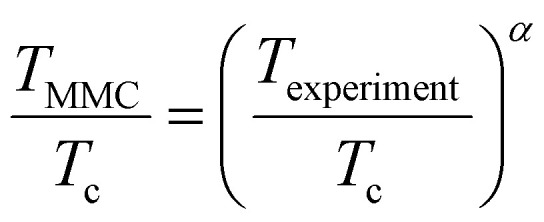
where *T*_MMC_ is the temperature of the MMC and *T*_experiment_ represents the real temperature that we would need in an experiment. The parameter *α* is just the effective rescaling factor that can be obtained from experimental measurements of the magnetization with temperature. Eqn (53) is called the quantum rescaling of the temperature in the MMC.

**Fig. 7:**
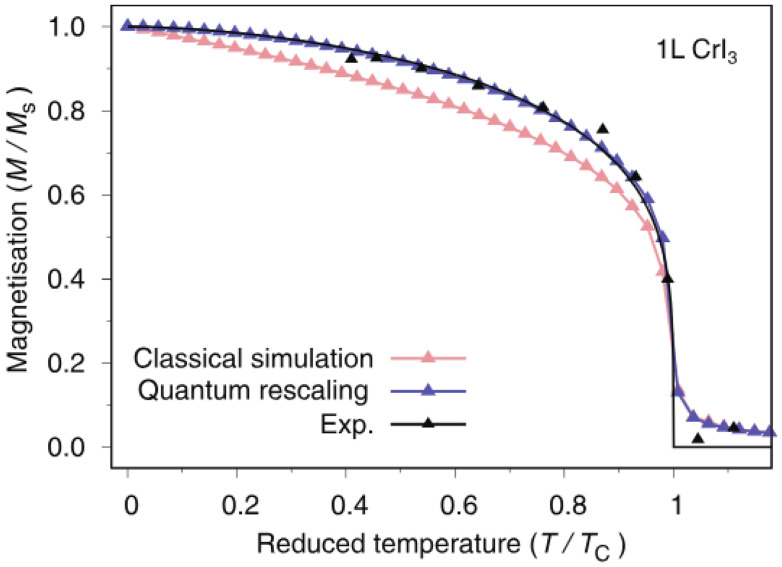
Comparison of the experimental magnetization and the one obtained from a Metropolis Monte Carlo method with the non-Heisenberg Hamiltonian in ref. 205 before and after the temperature rescaling of eqn (53). The non-Heisenberg Hamiltonian is a Heisenberg Hamiltonian with SIA, exchange anisotropy on the *z*-axis and biquadratic exchange. The symmetric exchange and the anisotropic exchange were taken up to three nearest-neighbours where as the biquadratic exchange was considered only to nearest neighbours. Figure extracted from ref. 205 © 2020 Wiley-VCH GmbH.

The results of applying this method are shown in Fig. 7 for 2D CrI_3_.^205^ The plot clearly shows that the magnetization given by the MMC underestimates the experimental magnetization up to the critical point, featuring an unphysical linear decrease of the magnetization for low temperatures. However, after the quantum rescaling, the magnetization overlaps with the experimental one even for low temperatures. Same trend is observed for bulk CrI_3_ in ref. 205 and for bulk metals Co, Fe, Ni, Gd in ref. 249.

To summarize, when looking for values of the magnetization at low temperatures, the MMC method will not give accurate results. Fortunately, when some experimental data is available, the quantum rescaling helps to recover the whole magnetization *vs. T* plot trivially by using (53).

##### 5.3.2.2. Available codes implementing Monte Carlo spin dinamics

There are already available codes implementing MMC for spin lattices. Some examples are VAMPIRE,^33,249,251,252^ SPIRIT^253,254^ and FIDIMAG.^255^ The code UPPASD^256^ incorporates the thermal relaxation by Langevin Dynamics^257^ instead of MMC, but this approach leads to analogous results for the critical temperature.

VAMPIRE features an extended manual describing all the available options. The developers have implemented an adaptative spin rotation scheme for the MMC that aims dynamically for a 0.5 acceptance rate.^247^ It also includes a tensorial interaction input that allows to insert manually the strength of the interactions. Hence, the user can insert manually as many interactions as needed.

SPIRIT stands out for featuring its own GUI and AiiDA^120–122^ plugin package,^254^ allowing an immediate insertion in automated workflows (more information in the corresponding section).

FIDIMAG MMC is contrained to considering only symmetric exchange interactions, DMI, cubic anisotropy and an external magnetic field.

For more details about the individual codes, the reader is referred to the corresponding references.

### 5.4. The Green's functions method

The magnetization with temperature can be calculated by employing Green's functions. Let us define the retarded Green Function of Operators *A*(*t*) and *B*(*t*′) in the Heisenberg picture:^34,225^

54 *G*_AB_(*t*,*t*′) := 〈〈*A*(*t*); *B*(*t*′)〉〉 := −*iθ*(*t* − *t*′)〈[*A*(*t*),*B*(*t*′)]〉

where *θ*(*t*) is the step function. And we are writing:55
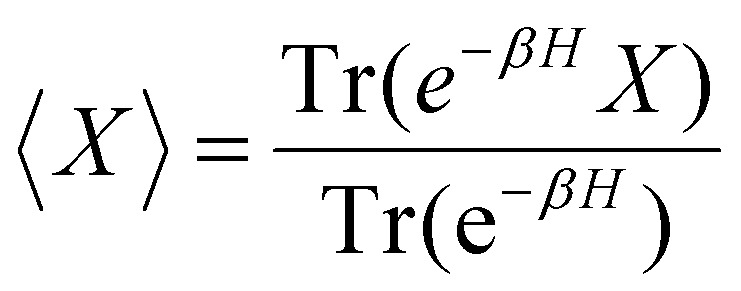


Exploiting the properties of these functions and their Fourier transforms, the following equation can be obtained:56
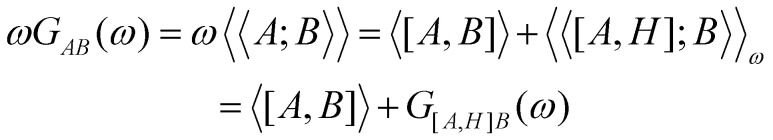


Which is an algebraic equation and is therefore easier to solve than a differential equation. We can see how this equation relates one Green function with another one that involves a commutation with the Hamiltonian. We say in this context that the equation is relating a Green function with a higher order Green function.^34^ This relation could be extended indefinitely for higher order Green functions so in practice, the chain has to be stopped at a certain order so that a system of equations is obtained that yields the Green functions. The chain is stopped by factoring the Green function of a certain order in terms of those of less order. This process is called decoupling.^34^ The utility of these decouplings in our context lies on how they can isolate the value of the 〈*Ŝ*^*z*^〉 which serves to calculate the magnetization with temperature of a ferromagnet thanks to the translation symmetry.

Consider the operators *S*_*i*_^−^ and *S*_*i*_^+^ and let us denote *G*_*ij*_(*ω*) = 〈〈*S*_*i*_^−^;*S*_*i*_^+^〉〉. We are omitting the hat over the spin operators on purpose for the sake of simplicity.57
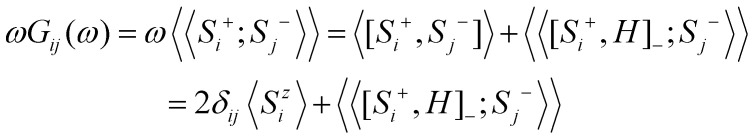
where we have used:58
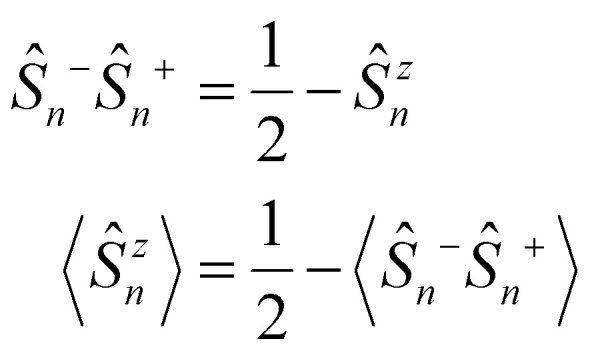


Before proceeding on, we need to specify a magnetic atomistic Hamiltonian. We will follow the process and notation in ref. 34 for a Heisenberg Hamiltonian with single ion anisotropy along the *z*-axis:59
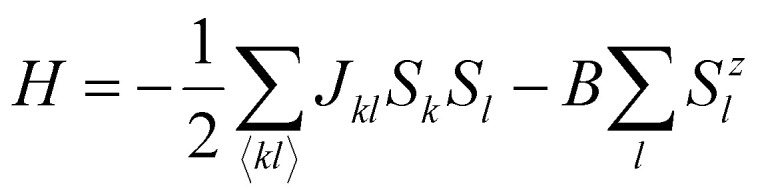
60
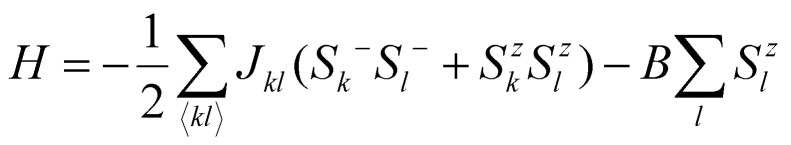


The next term to compute is:61
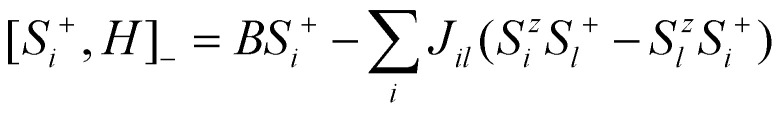


With this equality we can write (57) as:62
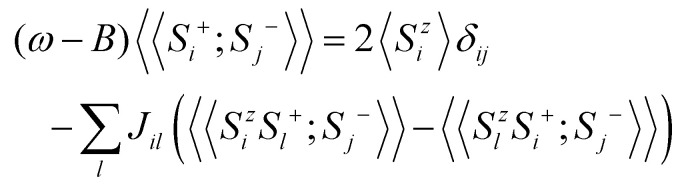


In order to simplify further, we need to introduce a decoupling. The decoupling known as Random Phase Approximation (RPA) goes as follows:63
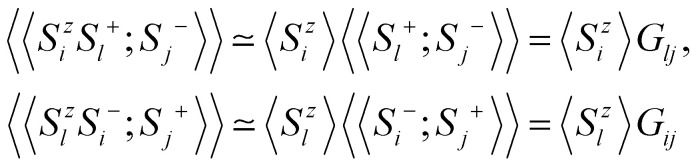


And the justification for this approach is purely heuristic.^86^ Note that implicitly, the RPA is setting *S*^*z*^ = 〈*S*^*z*^〉, which is an assumption that is valid only at low or far from the critical point temperatures. Using the RPA (63) in (62), the Fourier expansions and the translational invariance of the ferromagnet (〈*S^z^_i_*〉 = 〈*S^z^_j_*〉 = 〈*S*^*z*^〉), one can arrive to the system of equations:64
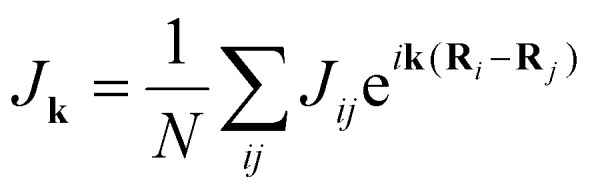

65 *ω*_**k**_^RPA^ = *B* + 〈*S*^*z*^〉(*J*_0_ − *J*_**k**_)

66
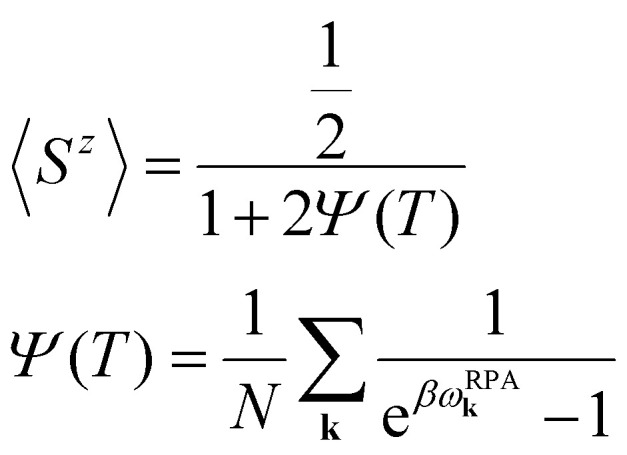


So finally, the Green functions method with the RPA yields a system of eqn (65) and (66) to be solved self-consistently in order to obtain the total magnetization of the ferromagnet with temperature.

The Random Phase approximation can be extended for systems with arbitrary spin, yielding:^225,232^67
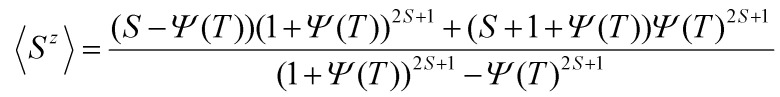


Another famous decoupling scheme is the Callen decoupling^34,258^

68 〈〈*S^z^_i_S*_*l*_^+^;*S*_*j*_^−^〉〉 ≃ 〈*S^z^_i_*〉〈〈*S*_*l*_^+^;*S*_*j*_^−^〉〉 − *α*〈*S*_*i*_^−^*S*_*l*_^+^〉〈〈*S*_*i*_^+^;*S*_*j*_^−^〉〉

where the parameter *α* is commonly set to *α* = 〈*S*^*z*^〉/2*S*^2^. The Callen decoupling leads to a system of equations similar to that for the RPA:69
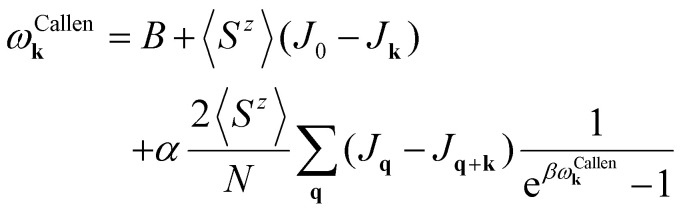
70
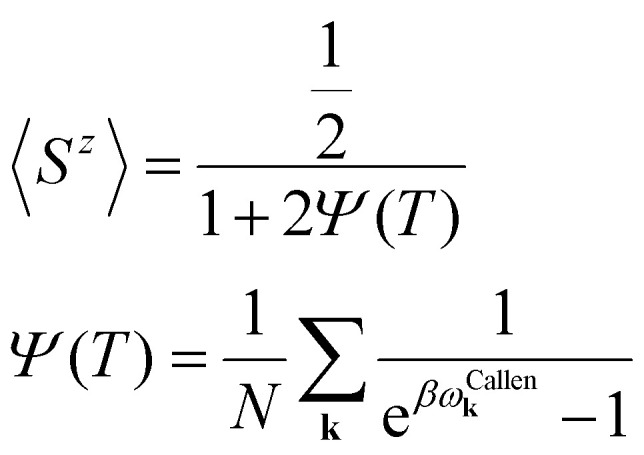


Which has the same form as for the RPA but the Callen decoupling introduces an additionally term in *ω*_**k**_ to be considered along the self-consistency process.

So in the end, using the Green's functions approach is about finding an appropriate decoupling scheme in order to arrive to a system of equations to be solved self-consistently to obtain the magnetization of the system. The decoupling schemes we have introduced here do not require using higher-order Green's functions but this is not a constrain and the philosophy can be extended to higher order Green's functions.

One of the main disadvantages of this approach is that every single atomistic magnetic Hamiltonian has to be studied separately, including both the derivation of the equation as well as their implementation into a code. Additionally, the decoupling process can be regarded as opaque in comparison with other methods that have a more clear physical meaning. Last but not least, every single decoupling scheme has a temperature regime in which it makes sense, but the extent on this validity interval is unknown and so far the only choice we have to test the method is *via* benchmarking and comparison with other methods.^14,232^

Despite the inconveniences, the Green's functions approach is widely used as well to compute critical temperatures of 2D magnetic materials as shown in recent literature.^14,229,232,259^

#### 5.4.1. Models to fit the magnetization

We already know that from the critical temperature and above, ferromagnets present zero magnetization. Therefore, the critical temperature can be estimated by observing the evolution of the magnetization of the system as a function of the temperature. For a fully systematic calculation of the Curie temperature, it is essential to have a model to fit the magnetization curve. The dependence of the magnetization at low temperatures can be obtained with linear spin-wave theory. Using eqn (47). For example, for a 3D material with a dispersion 
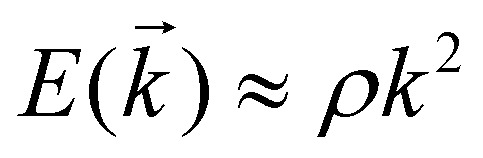
 for low 
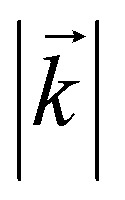
 leads to the famous Bloch equation^32,225^ and including more terms in the expansion of results in the appearance of terms proportional to *T*^5/2^ and *T*^7/2^.^32^

For 2D magnets, using eqn (47) with a dispersion relation as (42) without spin-wave gap results in no magnetic order at nonzero temperature. With nonzero spin-wave bandgap, the dependence can be obtained by solving (47), which gives the dependence for low temperature.

The magnetization in the vicinity of the critical point is predicted by renormalization group theory, which predicts for the Ising and Heisenberg Hamiltonians that the magnetization close to the critical point evolves as 
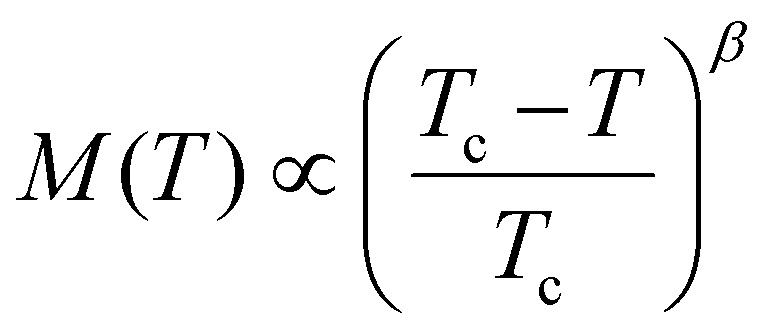
 where *β* is the so called critical exponent.^260^ In fact, for these magnetic Hamiltonians, the magnetization close to the critical temperature is well described by the equation:71
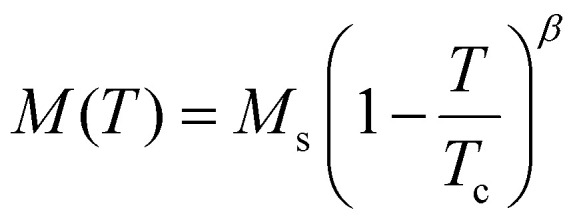
where *M*_s_ is the saturation magnetization, the value of the critical exponent depends on the dimensionality of the system and the dimensionality of the spin lattice. For a 2D material, the exponent is predicted to be *β* = 1/2 for an Ising Hamiltonian.^13,261^ There is no critical exponent for 2D materials and a Heisenberg Hamiltonian since the Mermin–Wagner theorem forbids the existence of long range magnetic order in this situation (for an infinite system size). However, there are already known critical exponents for the 3D counterpart.

It is reasonable to assume that a more general Hamiltonian would follow a similar behaviour to (71) in the vicinity of the critical point. However, the exact value of the exponent is unknown and in fact it will depend on the exact form of the Hamiltonian. However, we can use the value of the critical exponent as a metric of how Ising-like the material is *i.e.* the closer the critical exponent is to 1/8, the more Ising like it is.

Using the temperature rescaling in (53) in (71) leads to the so called Curie–Bloch equation:^249^72
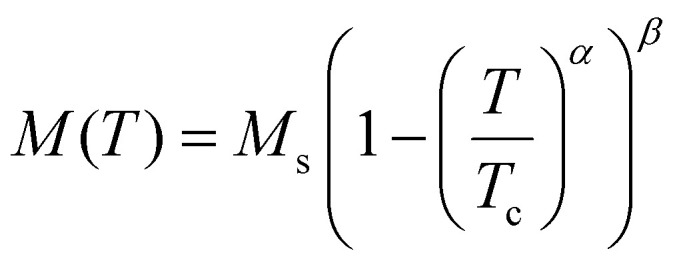


This new model solves the problem of the difference in shape of the experimental *vs.* calculated *M*(*T*) curves for low temperatures. The magnetic Hamiltonians and the statistical distribution in the MMC are of classical nature and therefore, they do not precisely take into account quantum effects that are relevant at low temperatures. By carrying out this temperature rescaling, the shape of the *M*(*T*) curve is improved, replicating the shape of experimental curves.^205^

### 5.5. Critical temperatures fitted from Montecarlo results

**Fig. 8:**
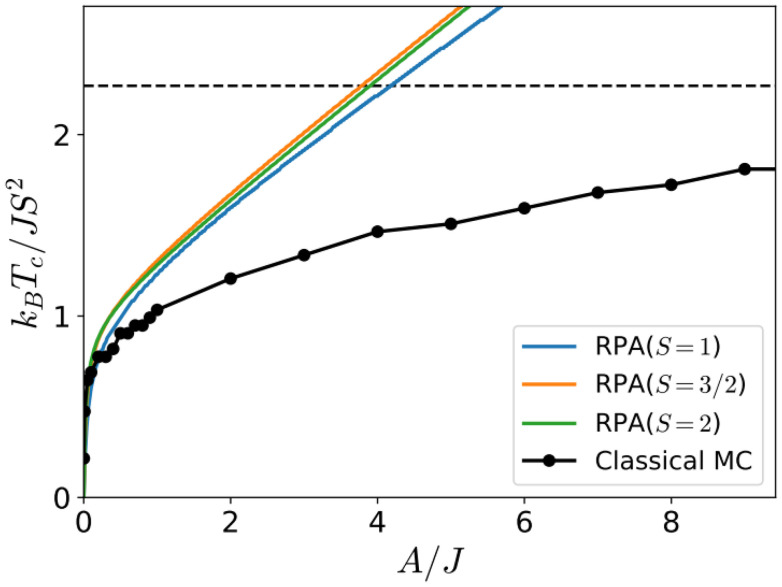
Critical temperature for a square lattice as a function of rescaled SIA (*A*/*J*) calculated for *S* = 1, *S* = 3/2, and *S* = 2 with ferromagnetic exchange coupling using the RPA (In this context, RPA refers to nonlinear spin wave theory). The dashed line represents the critical temperature given by the Ising Hamiltonian, which sets the case for infinite SIA. Figure extracted from ref. 15 D. Torelli and T. Olsen, Calculating critical temperatures for ferromagnetic order in two-dimensional materials, *2D Materials*, 2018, **6**, 015028. Published 17 December 2018. DOI: 10.1088/2053-1583/aaf06d. © IOP Publishing. Reproduced with permission. All rights reserved.

MMC is computationally expensive and its cost increases with the number of atoms and the number of interactions considered. However, its physical input is just the magnetic Hamiltonian and therefore, its results should depend only on its parameters. Two different systems with the same magnetic Hamiltonian should give the same result. This motivates the goal of having a closed formula relating the critical temperature with the Hamiltonian parameters, even if it is empirical. This was the goal of Torelli and Olsen.^15^ Using the following Hamiltonian that includes both SIA and exchange anisotropy for a single magnetic ion per unit cell:73
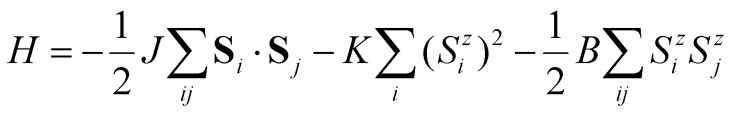
they carried out nonlinear spin-wave theory and MMC simulations for different ratios of the Hamiltonian parameters and different 2D lattices. Then, they fitted the results to an empirical model. The results for the case *B* = 0 for a square lattice are shown in Fig. 8.

The first remarkable result is that nonlinear spin-wave theory dramatically fails for strong SIA ratio *A*/*J*. The authors attribute this failure to the appearance of strong magnon–magnon interactions that are not well described by the characteristic mean field treatment in nonlinear spin-wave theory. The failure is so bad that the predicted critical temperature even surpasses the Ising temperature which corresponds to the infinte SIA case.

However, nonlinear spin-wave theory agrees well with MMC at low SIA ratio. In fact, it does a better job for low SIA ratio since it predicts a 0 Curie temperature for no SIA (completely isotropic system in this case) where as MMC predicts a nonzero *T*_c_, contradicting the Mermin–Wagner theorem.

**Fig. 9:**
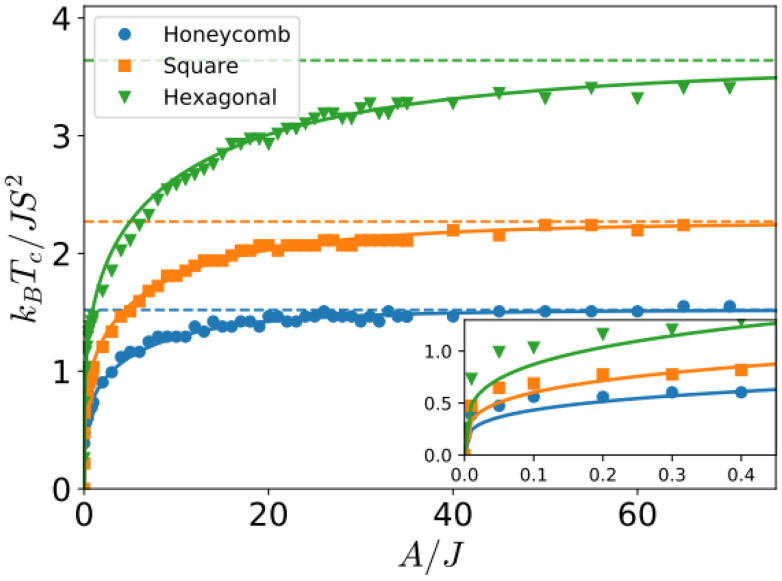
Critical temperature as a function of scaled SIA (*A*/*J*) calculated with classical Monte Carlo simulations for honeycomb, square, and hexagonal lattices with ferromagnetic exchange. The solid lines are obtained from the empirical fitting function (74). The Ising limit is indicated by dashed lines for the three lattices. Figure extracted from ref. 15 D. Torelli and T. Olsen, Calculating critical temperatures for ferromagnetic order in two-dimensional materials, *2D Materials*, 2018, **6**, 015028. Published 17 December 2018. DOI: 10.1088/2053-1583/aaf06d. © IOP Publishing. Reproduced with permission. All rights reserved.

The critical temperatures obtained by MMC are shown in Fig. 9 for three types of 2D lattices. The MMC results agree with the Ising limit (dashed lines) for high SIA ratio. The key result in ref. 15 is the expression of the critical temperature obtained by fitting the results of the MMC in Fig. 9:74
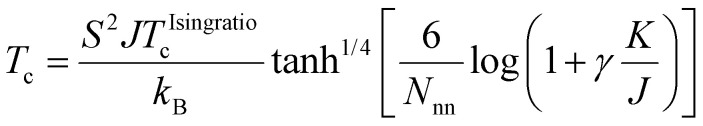
where *N*_nn_ is the number of nearest neighbours, *γ* = 0.033 and *T*^Isingratio^_c_ is the value of 
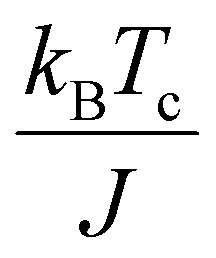
 for the Ising model and the corresponding lattice (see Table 2). It is important to remark that the empirical formula (74) applies for systems with the given lattices and the Hamiltonian in (73) with *B* = 0 and with a single magnetic ion per unit cell. Still, the formula serves as a cheap first approximation of the critical temperature and can be combined to obtain a rough estimation of the necessary upper bound of an MMC simulation.

When *B* ≠ 0 and *S* ≠ 1/2, they found an analogous empirical relation:75
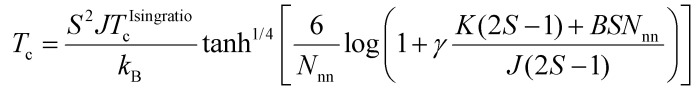


### 5.6. Critical temperatures fitted from different methods

At this point, many ways of calculating the Curie point of 2D ferromagnets have been introduced and one may wonder how compatible the results of the different methods can be. In the each method's section we have discussed their limitations in a qualitative manner, but a more ambitious scientist might still want to have a quantitative relation or a way to compare the output Curie point of the different methods. We will discuss the work of Tiwari *et al.*^14^ for this purpose. In this work, they calculated the Curie point both with computational methods and with a closed formula they propose for the 2D magnets featured in the Computational 2D Materials Database^22,23^ (C2DB). The three methods they used were: non-linear spin-wave theory, the Green's functions method and MMC.

The magnetic Hamiltonian they proposed to model the ferromagnets is:76
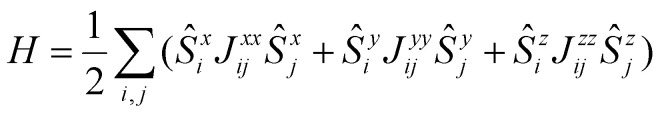
77
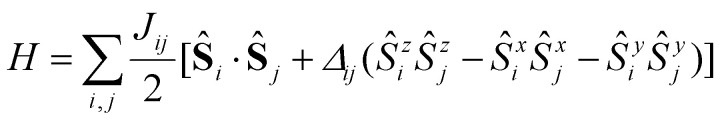


Which features only two-ion anisotropy (no SIA). The following notation has been used: 
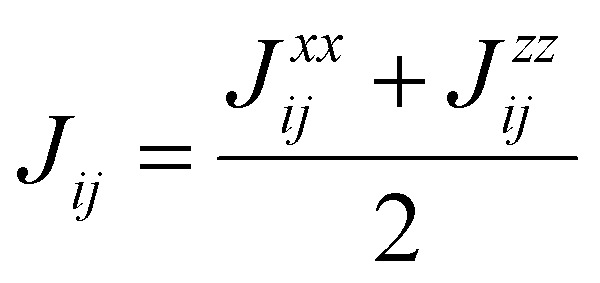
 and 
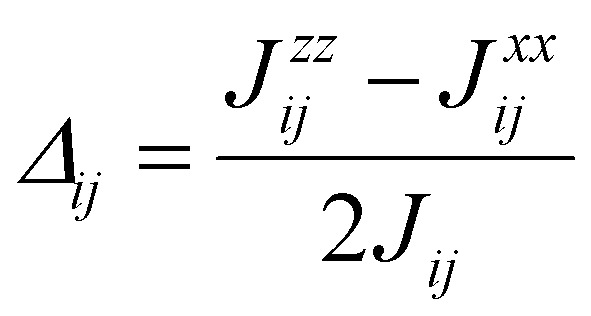
 is the out-of-plane anisotropic exchange strength. With this notation *J^xx^_ij_* = *J^yy^_ij_* = *J*_*ij*_(1 − *Δ*_*ij*_) and *J^zz^_ij_* = *J*_*ij*_(1 + *Δ*_*ij*_).

Tiwari *et al.* looked for 2D magnets in C2DB with positive first neighbours exchange constant *J*_*ij*_ := *J* and anisotropic exchange strenght *Δ*_*ij*_ := *Δ*_NN_. They found 34 2D magnets with *J* > 0 and *Δ*_NN_ > 0. For those 34 magnets, they calculated the critical temperature using non-linear spin-wave theory, the Green's functions method and MMC. With the resulting *T*_c_ values, they fitted the results to the following model:78
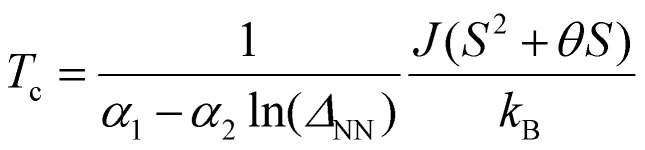
where *θ* = 1 for the Green's functions method and spin-wave theory and *θ* = 0 for the MMC. *α*_1_ and *α*_2_ are parameters to fit as a function of *J*,*S* and *Δ*_nn_ for each method and type of lattice. The results they obtained for these two parameters are shown in Table 4 for the Honeycomb, hexagonal and square lattices. The authors state that this formula is only valid when *Δ*_nn_ ≤ 0.2 since it is based on the theoretical approach in ref. 262.

**Table 4:** Resulting parameters for eqn (78) across different lattices and methods. Data extracted from ref. 14

Lattice	Parameter	MC	Green	RNSW
Honeycomb	*α*_1_	0.49	0.07	0.40
	*α*_2_	0.14	0.37	0.62
Hexagonal	*α*_1_	0.24	0.24	0.32
	*α*_2_	0.045	0.14	0.21
Square	*α*_1_	0.37	0.34	0.43
	*α*_2_	0.08	0.24	0.36

**Fig. 10:**
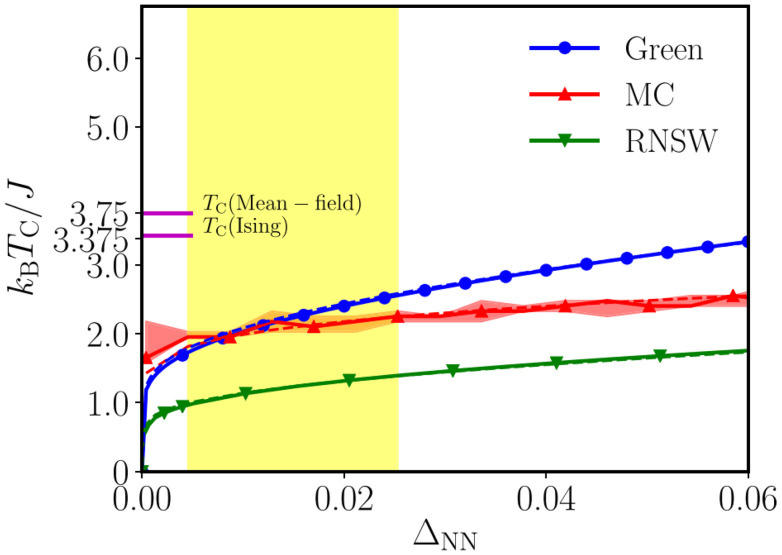
Comparison of the calculated critical temperatures with the Green's functions methods, the MMC and non-linear spin-wave theory (RNSW) as a function of the nearest neighbour anisotropic exchange strength *Δ*_NN_ for a honeycomb lattice. The dashed lines represent the fit using eqn (78). The solid line for the MC shows 25th to 75th percentile of the calculated Curie temperature. The yellow shaded area shows the region where the MC and the Green's function methods have a difference of less than 10%. The horizontal tick show the Ising limit (1.52*S*^2^, with *S* = 3/2) and the quantum mean field (1/3[*S*(*S* + 1)*N*_NN_], *N*_NN_ = 3 for honeycomb lattice). Figure extracted from ref. 14 without changes under Creative Commons Attribution 4.0 International License https://creativecommons.org/licenses/by/4.0/.

The calculated values for *T*_c_ with each method and the resulting fit for a honeycomb lattice are plotted in Fig. 10. The same plot for an hexagonal and a square lattice can be found in the ESI of ref. 14 but they follow the same trends as Fig. 10, hence, we discuss only the results for the honeycomb lattice.

Fig. 10 has some noteworthy results. Firstly, among the three methods, MMC is the only one that predicts a nonzero critical temperature in the absence on anisotropy, contradicting the Mermin–Wagner theorem. Secondly, nonlinear spin-wave theory gives always a lower bound of the critical temperature with respect to the other two methods. Additionally, the Green's functions method serves as an upper bound for the MMC for high anisotropic exchange strength. Last but not least, there is an interval of anisotropic exchange strength (the yellow shaded area in Fig. 10) in which the three methods differ in less than 10%. These results, combined with those in Fig. 8 suggest that spin-wave theory is not an appropriate method when treating high SIA or exchange anisotropy.

### 5.7. Brief review of the methods and conclusion

We would like to close this section with a brief discussion about the limitations and the use cases of the methods explained above.

We started introducing the mean field theory expression for the critical temperature and the critical temperatures for the Ising model in 2D lattices. The first one does not account for dimensionality nor anisotropy effects and the second one assumes infinite single ion anisotropy. These facts make them both usually huge overestimations of the critical temperature of the 2D magnets as shown in Fig. 10. Moreover, these formulas are obtained when considering only isotropic exchange and *z*-component of the anisotropic exchange tensor respectively. Therefore, they do not take into account other effects such as DMI. Their main advantage is the fact that computing them is trivial and we can use their overestimation as an upper bound for the critical temperature. This upper bound can be used for example as an input for the maximum temperature for a Metropolis Monte Carlo or for a spin-wave theory algorithm.

Spin wave theory in both its linear and nonlinear flavours has the strong disadvantage that its explicit development has to be done for every different atomistic magnetic Hamiltonian. We are then forced to write the equations in advance before writing an automatic code. Moreover, the expressions can get long and complicated when including all the anisotropies we explained in this text. Additionally, the truncation of the Holstein–Primakoff expansion leads to neglecting the magnon–magnon interactions that can have importance at high temperatures. This is the reason why the magnetization obtained with spin-wave theory at high temperatures will not be reliable. Moreover, in ref. 15 and Fig. 8, it is shown how spin wave theory fails to give a sensible value of the critical temperature for high SIA/*J* situations. The authors attribute this result to magnon–magnon interactions introduced by SIA/*J* that are not correctly described within NLSW theory. However, for low SIA/*J* regimes, Fig. 8 and 10 show how spin-wave theory yields results compatible with MMC. In fact, it gives 0 *T*_c_ for zero anisotropy, something that MMC does not show. Additionally, there is an anisotropic exchange window shown in Fig. 10 in which MMC and NLSW theory differ no more than 10%. For all these reasons, we consider spin-wave theory suitable only for low SIA/*J* and exchange anisotropy cases, which in the end will give low *T*_c_ values.

For cases with higher SIA/*J*, MMC is more suitable as concluded from Fig. 8 and 9. We also want to highlight that treating the spins as classical particles is an assumption with validity only when quantum effects become unimportant because they are quenched by temperature. Consequently, we consider MMC the most appropriate method when looking for above room temperature 2D magnets. Its main downsides are its extremely high computational cost and its unphysical low magnetization obtained for below *T*_c_ temperatures as explained for Fig. 7. However, this unusual low magnetization is not a problem if we are only interested on obtaining *T*_c_.

The Green's functions method presents similar problems to those of spin-wave theory. Firstly, the equations have to be derived in advance for every atomistic magnetic Hamiltonian, and these get more and more complex when the number of different interactions is increased. Secondly, the decoupling scheme dictates the validity of the approach. For example, the RPA decoupling sets *S*^*z*^ = 〈*S*^*z*^〉, which is an assumption valid only at low temperatures when fluctuations are low. The Callen decoupling serves as an interpolation between the RPA and a high temperature regime^34^ and therefore, there is no other way to estimate its accuracy rather than doing benchmarks as done in ref. 14 with Fig. 10. This figure along with Fig. 8 discards spin-wave theory as a good method in cases with high SIA/*J* or exchange anisotropy and suggest that there is a regime of exchange anisotropy in which MMC and the Green's functions method give compatible results. The comparison of the methods as a function of SIA/*J* is done in ref. 232 and shown in Fig. 12 for a honeycomb lattice. This figure finally shows that for low SIA/*J* and zero anisotropic exchange, all methods give comparable results. It is worth noting that NLSW theory remains being a lower bound for the rest of the methods where as the RPA serves as an upper bound.

In conclusion, once the parameters of the chosen Hamiltonian are known, choosing the method to calculate the Curie temperature is not an arbitrary choice. The researcher has to choose the method depending on the value of the parameters, its estimation of the Curie temperature with the mean field approach and the availability of computational resources. Last but not least, we want to clarify that the methods we introduced above in section 5 can be extended to antiferromagnetic materials.

## 6. Summary and outlook

Despite remarkable advances, simulating 2D magnetic materials from first principles remains a formidable challenge due to the extreme sensitivity of magnetic interactions to subtle structural, electronic, and computational parameters. The reduced dimensionality of these systems introduces unique theoretical and practical difficulties, particularly in capturing the intricate anisotropy mechanisms, and often delicate magnetic ordering. Unlike their 3D counterparts, 2D magnets rely critically on anisotropic interactions – such as single-ion anisotropy and different types of anisotropic interactions – to stabilize long-range magnetic order against thermal fluctuations, in line with the Mermin–Wagner theorem. However, these energy contributions typically lie in the sub-meV regime, pushing the limits of accuracy for standard DFT methods. Furthermore, the layered nature of many 2D materials introduces complex indirect exchange pathways mediated by ligands or chalcogen atoms, requiring careful electronic structure modeling. While approaches like DFT+*U* and hybrid functionals have improved our ability to capture localized electron behavior, the absence of universal, *ab initio* protocols for determining magnetic ground states (especially noncollinear or incommensurate orders) remains a major bottleneck. Addressing these challenges will require more systematic treatments of magnetic anisotropies, automate the exploration of large configuration spaces (*e.g.*, *via* generalized Bloch theorem or spin-constrained DFT), and expand beyond traditional collinear models. Future progress will hinge on combining accurate, systematically parameter-free methods (*e.g.*, self-consistent DFT+*U*/*V*, hybrid functionals, and generalized Bloch theorem-based approaches) with machine learning-driven optimization and high-throughput screening will be crucial to identify new candidate materials. Coupling these advances with high-throughput workflows and experimental feedback will be key to uncovering novel 2D magnets with robust and tunable properties, particularly those that can operate above room temperature for next-generation spintronic and quantum devices.

**Fig. 11:**
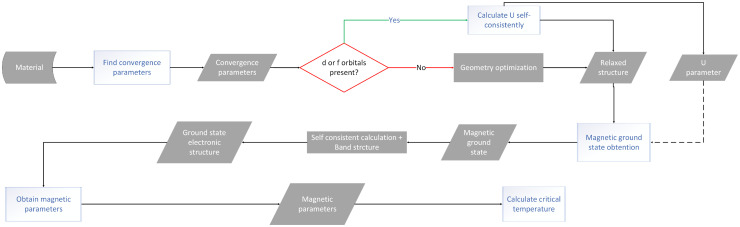
Sketch of the worfklow to follow when modelling 2D magnetic materials from DFT to the calculation of their critical temperature. All the steps in this workflow are covered throughout this review paper.

As a summary, in this review we have revisited the fundamentals of magnetic interactions in 2D materials from the approximated description of these systems using DFT to the picture of atomistic magnetic models. We have discussed the main critical points to consider in the simulation of magnetism in this kind of materials, which introduce important challenges driven by the small energies of both exchange and anisotropies and the complexity of the models that involve spin–orbit effects, correlation and van der Waals interactions. We present a guide to address the important challenges related to the modelling of 2D materials, mainly the selection of the Hubbard parameters, descriptions of the van der Waals interactions and approaches to properly model the magnetic ground state and compute exchange parameters, anisotropies and critical temperatures. This review also presents a comparison of the different DFT codes available, highlighting their relevant features for magnetism in DFT. In the end, the whole work compiles the necessary tools to model 2D magnetic materials from DFT to their critical temperature following the steps shown in Fig. 11.

**Fig. 12:**
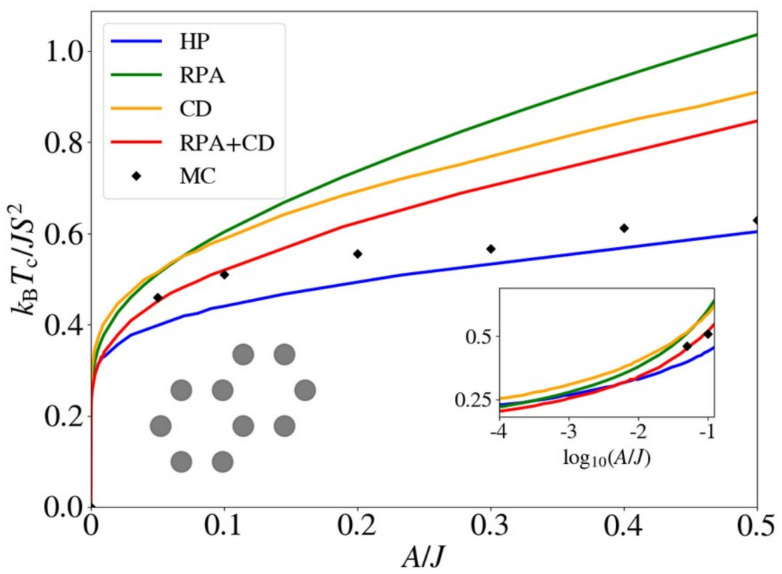
Comparison of the critical temperatures obtained for a 2D honeycomb lattice as a function of the SIA/*J* ratio (*A* represents the SIA). The anisotropic exchange is set to 0. HP represents nonlinear spin-wave theory with the Holstein–Primakoff expansion. RPA and CD refer to the Green's functions method with the random phase approximation and the Callen decoupling respectively. RPA + CD refers to a combination of both and MC refers to the Metropolis Monte Carlo algorithm. Figure extracted from ref. 232: Varun Rajeev Pavizhakumari, Thorbjørn Skovhus and Thomas Olsen, Beyond the random phase approximation for calculating Curie temperatures in ferromagnets: application to Fe, Ni, Co and monolayer CrI_3_, *Journal of Physics: Condensed Matter*, 2025. Accepted manuscript online 6 January 2025. DOI: 10.1088/2053-1583/aaf06d. © IOP Publishing. Reproduced with permission. All rights reserved.

We finally like to highlight the importance of all the content, tools and guidelines introduced in this section by comparing computed critical temperatures from DFT with the values obtained experimentally. Table 5 contains the critical temperature of several 2D magnets obtained from DFT as well as the DFT flavour used and the method to compute the transition temperature. The critical temperature is a good magnitude to illustrate the complexity of the modeling because it suffers from the propagation of all the errors that have appeared during the modeling process.

**Table 5:** Compilation of calculated critical temperatures from DFT results

Monolayers	Code	DFT flavour (*U* or standard)	*T*_c_/*T*_N_ (K)	*T*_c_/*T*_N_ method	Experimental *T*_c_/*T*_N_ (K)	*J*_1_ (meV)	Ref.
CrI_3_	VASP	PBE	38.42	Montecarlo fit (75)	45^1^	1.9	25
	VASP	PBE+*U* (*U* = 2)	46.06	Montecarlo fit (75)		1.92	25
	VASP	PBE+*U* (*U* = 1.7)	42.2	Montecarlo		1.06	224
	GPAW	PBE	28	Montecarlo fit (75)		0.97	16
	GPAW	PBE^23^	41	Green's Functions		1.025^23^	14
	GPAW	PBE^23^	31	Montecarlo		1.025^23^	14
	GPAW	PBE^23^	23	NLSW		1.025^23^	14
	Quantum Espresso	PBE+*U* (4.55)	94.2	Green's Functions		3.197	124
VSe_2_-2H	Quantum ATK	PBE+*U* (*U* = 2)	249.69	Montecarlo	418^263^ (multilayer)	19.52	5
	VASP	PBE+*U* (*U* = 2, *J* = 0.87)	291	Montecarlo		38.8	264
	VASP	PBE	359	Montecarlo		18.93	265
	VASP	PBE	427.8	Exchange-mean-field		44.85	266
CrSBr	Quantum Espresso	PBE+*U* (*U* = 3)	122	NLSW	146^162^	3.54	125
	VASP	PBE+*U* (*U* = 3)	160	Montecarlo		3.87	267
	VASP	PBE+*U* (*U* = 4, *J* = 0.9)	175	Montecarlo		3.65	50
	VASP	PBE+*U* (*U* = 3)	168	NLSW		5.68	268
CrBr_3_	VASP	PBE	23.53	Montecarlo fit (75)	27,^269^ 34^270^	1.24	25
	VASP	PBE+*U* (*U* = 2)	27.52	Montecarlo fit (75)		2.56	25
	VASP	PBE+*U* (*U* = 1.7)	23.1	Montecarlo		0.67	224
	Quantum Espresso	PBE+*U* (4.20)	55.3	Green's Functions		2.682	124
VTe_2_-2H	Quantum ATK	PBE+*U* (*U* = 2)	126.61	Montecarlo	Not done yet to our best knowledge	9.84	5
	GPAW	PBE^23^	387.6	Green's Functions		21.96^23^	14
	GPAW	PBE^23^	371.1	Montecarlo		21.96^23^	14
	GPAW	PBE^23^	263.4	NLSW		21.96^23^	14
	VASP	PBE+*U* (*U* = 2 *J* = 0.87)	553	Montecarlo		44.3	264
	VASP	PBE+*U* (*U* = 2)	301	Mean field theory		29.25	271
CrCl_3_	VASP	PBE	13.49	Montecarlo fit (75)	12.95,^272^ 17 (2 layers)^269^	0.89	25
	VASP	PBE+*U* (*U* = 2)	16.77	Montecarlo fit (75)		1.2	25
	VASP	PBE+*U* (*U* = 1.7)	12.1	Montecarlo		0.39	224
	GPAW	PBE	9.2	Montecarlo fit (75)		0.64	16
Fe_3_GaTe_2_	VASP	LDA	320	Montecarlo	240^273^ 367 (few layers)^274^	57.18	165
	QE	GGA	320	Montecarlo		35.52	164
Fe_3_GeTe_2_	Wien2k	LDA+*U* (*U* = 4)	152	Montecarlo	130^3^	11.91	275
	VASP	LDA	154	NLSW		1.21	276

**Fig. 13:**
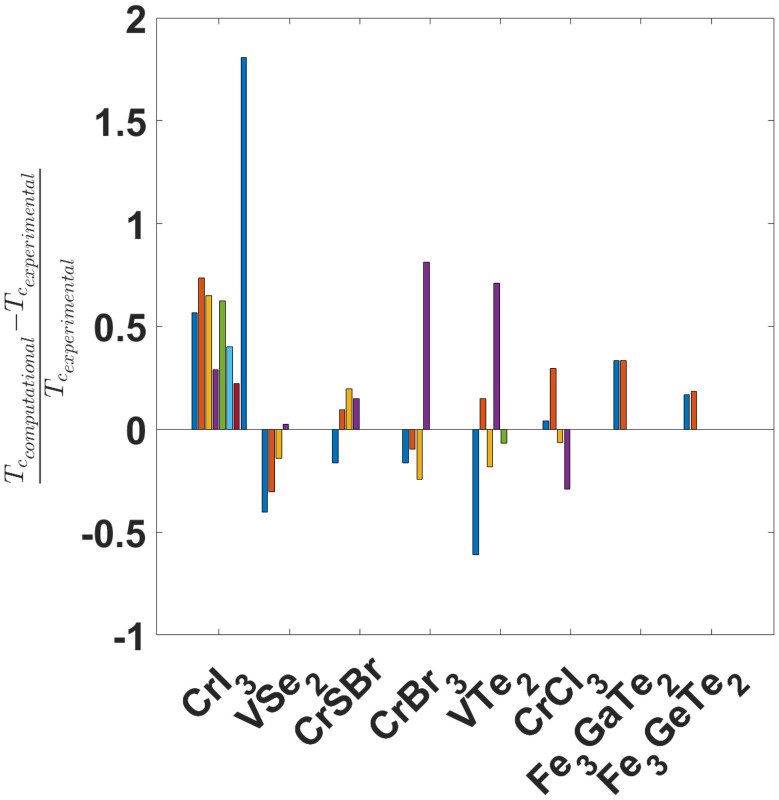
Comparison of calculated critical temperatures of 2D magnets with the experimental value. The data shown comes from Table 5. Since we are not aware of any experimental estimation of the critical temperature of VTe_2_, we take its value as the mean from those in Table 5.

The normalized deviation of the computed critical temperature from the experimental value is shown in Fig. 13. The plot shows how some approaches have lead to a rather good estimation of the critical temperature (with error of less than 20% or even 10%) while others fail dramatically. Our take home message with this figure is that full control during the whole modeling process is essential to end up getting accurate results and to understand the limitations of the work done.

We expect this review to motivate future readers, to get familiar with the critical steps during the process of materials simulation using DFT and to be aware of the challenges and limitations of the state of the art.

## Author contributions

Jose-Hugo García was responsible of the conceptualization, funding acquisition, project administration, supervision, validation, writing, reviewing and editing. S. Roche participates in the project administration and editing. Stephan Roche improved the manuscript and contributed to the broad scope discussion. Dorye. L Esteras and Jaime Garrido Aldea have contributed with the investigation, the original draft preparation and have reviewed and edited the original draft.

## Conflicts of interest

The authors declare that there are no conflicts of interest that could have influenced the content, analysis, or conclusions of this work.

## Data Availability

No new primary research data, software, or code were generated or analyzed in this review. All data supporting the findings discussed in this article are derived from previously published studies, which are cited appropriately in the text and reference section. Additionally, images included in this review have been reproduced from other sources after obtaining the necessary copyright permissions.

## References

[cit1] Huang B., Clark G., Navarro-Moratalla E., Klein D. R., Cheng R., Seyler K. L., Zhong D., Schmidgall E., McGuire M. A., Cobden D. H., Yao W., Xiao D., Jarillo-Herrero P., Xu X. (2017). Nature.

[cit2] Gong C., Li L., Li Z., Ji H., Stern A., Xia Y., Cao T., Bao W., Wang C., Wang Y., Qiu Z. Q., Cava R. J., Louie S. G., Xia J., Zhang X. (2017). Nature.

[cit3] Fei Z., Huang B., Malinowski P., Wang W., Song T., Sanchez J., Yao W., Xiao D., Zhu X., May A. F., Wu W., Cobden D. H., Chu J.-H., Xu X. (2018). Nat. Mater..

[cit4] Mermin N. D., Wagner H. (1966). Phys. Rev. Lett..

[cit5] Jafari M., Rudziński W., Barnaś J., Dyrdał A. (2023). Sci. Rep..

[cit6] Lado J. L., Fernández-Rossier J. (2017). 2D Mater..

[cit7] Kajale S. N., Hanna J., Jang K., Sarkar D. (2024). Nano Res..

[cit8] Elahi E., Dastgeer G., Nazir G., Nisar S., Bashir M., Akhter Qureshi H., kee Kim D., Aziz J., Aslam M., Hussain K., Assiri M. A., Imran M. (2022). Comput. Mater. Sci..

[cit9] Khan Y., Obaidulla S. M., Habib M. R., Gayen A., Liang T., Wang X., Xu M. (2020). Nano Today.

[cit10] Kobernik T. N., Kartsev A. I. (2024). J. Phys. Chem. Lett..

[cit11] Chowde Gowda C., Kartsev A., Tiwari N., Safronov A. A., Pandey P., Roy A. K., Ajayan P. M., Galvão D. S., Tiwary C. S. (2024). J. Mater. Chem. C.

[cit12] Huang X., Zhang L., Tong L., Li Z., Peng Z., Lin R., Shi W., Xue K.-H., Dai H., Cheng H., de Camargo Branco D., Xu J., Han J., Cheng G. J., Miao X., Ye L. (2023). Nat. Commun..

[cit13] Wang Q. H., Bedoya-Pinto A., Blei M., Dismukes A. H., Hamo A., Jenkins S., Koperski M., Liu Y., Sun Q.-C., Telford E. J., Kim H. H., Augustin M., Vool U., Yin J.-X., Li L. H., Falin A., Dean C. R., Casanova F., Evans R. F. L., Chshiev M., Mishchenko A., Petrovic C., He R., Zhao L., Tsen A. W., Gerardot B. D., Brotons-Gisbert M., Guguchia Z., Roy X., Tongay S., Wang Z., Hasan M. Z., Wrachtrup J., Yacoby A., Fert A., Parkin S., Novoselov K. S., Dai P., Balicas L., Santos E. J. G. (2022). ACS Nano.

[cit14] Tiwari S., Vanherck J., Van de Put M. L., Vandenberghe W. G., Sorée B. (2021). Phys. Rev. Res..

[cit15] Torelli D., Olsen T. (2018). 2D Mater..

[cit16] Torelli D., Moustafa H., Jacobsen K. W., Olsen T. (2020). npj Comput. Mater..

[cit17] Torelli D., Thygesen K. S., Olsen T. (2019). 2D Mater..

[cit18] Torelli D., Thygesen K. S., Olsen T. (2019). 2D Mater..

[cit19] Kabiraj A., Kumar M., Mahapatra S. (2020). npj Comput. Mater..

[cit20] Wang K., Ren K., Hou Y., Cheng Y., Zhang G. (2023). J. Appl. Phys..

[cit21] Giustino F., Lee J. H., Trier F., Bibes M., Winter S. M., Valentí R., Son Y.-W., Taillefer L., Heil C., Figueroa A. I., Plaçais B., Wu Q., Yazyev O. V., Bakkers E. P. A. M., Nygård J., Forn-Díaz P., Franceschi S. D., McIver J. W., Torres L. E. F. F., Low T., Kumar A., Galceran R., Valenzuela S. O., Costache M. V., Manchon A., Kim E.-A., Schleder G. R., Fazzio A., Roche S. (2021). J. Phys.: Mater..

[cit22] Haastrup S., Strange M., Pandey M., Deilmann T., Schmidt P. S., Hinsche N. F., Gjerding M. N., Torelli D., Larsen P. M., Riis-Jensen A. C., Gath J., Jacobsen K. W., Mortensen J. J., Olsen T., Thygesen K. S. (2018). 2D Mater..

[cit23] Gjerding M. N., Taghizadeh A., Rasmussen A., Ali S., Bertoldo F., Deilmann T., Knøsgaard N. R., Kruse M., Larsen A. H., Manti S., Pedersen T. G., Petralanda U., Skovhus T., Svendsen M. K., Mortensen J. J., Olsen T., Thygesen K. S. (2021). 2D Mater..

[cit24] Zhang H. (2021). Electron. Struct..

[cit25] Wines D., Choudhary K., Tavazza F. (2023). J. Phys. Chem. C.

[cit26] Kohn W., Sham L. J. (1965). Phys. Rev..

[cit27] Martin R. M. , Electronic Structure: Basic Theory and Practical Methods, Cambridge University Press, 2004

[cit28] Sødequist J., Olsen T. (2023). 2D Mater..

[cit29] Sødequist J., Olsen T. (2024). npj Comput. Mater..

[cit30] Xiang H., Lee C., Koo H.-J., Gong X., Whangbo M.-H. (2013). Dalton Trans..

[cit31] Liechtenstein A., Katsnelson M., Antropov V., Gubanov V. (1987). J. Magn. Magn. Mater..

[cit32] Yosida K. , Theory of magnetism, Springer, 1996

[cit33] Evans R. F. L., Fan W. J., Chureemart P., Ostler T. A., Ellis M. O. A., Chantrell R. W. (2014). J. Phys.: Condens. Matter.

[cit34] Fröbrich P., Kuntz P. (2006). Phys. Rep..

[cit35] White R. M. , Quantum Theory of Magnetism, Springer, Berlin, Heidelberg, 3rd edn, 2007

[cit36] Blundell S. , Magnetism in Condensed Matter, Oxford University Press, 1st edn, 2001

[cit37] Jiang X., Liu Q., Xing J., Liu N., Guo Y., Liu Z., Zhao J. (2021). Appl. Phys. Rev..

[cit38] Bloch F. (1929). Z. Phys..

[cit39] Madelung O. , Introduction to Solid-State Theory, Springer, Berlin, Heidelberg, 1978, vol. 2

[cit40] Perdew J. P., Burke K., Ernzerhof M. (1996). Phys. Rev. Lett..

[cit41] Arbuznikov A. V. (2007). J. Struct. Chem..

[cit42] Ising E. (1925). Z. Phys..

[cit43] Dzyaloshinskii I. (1957). et al.. Sov. Phys. JETP.

[cit44] Moriya T. (1960). Phys. Rev..

[cit45] Moriya T. (1960). Phys. Rev. Lett..

[cit46] Blügel S. and Bihlmayer G., in Magnetism of Low-dimensional Systems: Theory, John Wiley & Sons, Ltd, 2007

[cit47] Kartsev A. I., Obraztsov K. V., Lega P. V. (2023). J. Commun. Technol. Electron..

[cit48] Kartsev A., Augustin M., Evans R. F. L., Novoselov K. S., Santos E. J. G. (2020). npj Comput. Mater..

[cit49] Kartsev A., Malkovsky S., Chibisov A. (2021). Nanomaterials.

[cit50] Yang K., Wang G., Liu L., Lu D., Wu H. (2021). Phys. Rev. B.

[cit51] Boix-Constant C., Mañas-Valero S., Ruiz A. M., Rybakov A., Konieczny K. A., Pillet S., Baldoví J. J., Coronado E. (2022). Adv. Mater..

[cit52] Bruno P. (1991). Phys. Rev. B: Condens. Matter Mater. Phys..

[cit53] Spaldin N. A. , in Ferrimagnetism, Cambridge University Press, 2010, pp. 113–129

[cit54] Uhl M., Sandratskii L., Kübler J. (1992). J. Magn. Magn. Mater..

[cit55] Knöpfle K., Sandratskii L. M., Kübler J. (2000). Phys. Rev. B: Condens. Matter Mater. Phys..

[cit56] Sjöstedt E., Nordström L. (2002). Phys. Rev. B: Condens. Matter Mater. Phys..

[cit57] Mankovsky S., Fecher G. H., Ebert H. (2011). Phys. Rev. B: Condens. Matter Mater. Phys..

[cit58] Li Y., Yang B., Xu S., Huang B., Duan W. (2022). ACS Appl. Electron. Mater..

[cit59] Hohenberg P., Kohn W. (1964). Phys. Rev..

[cit60] Becke A. D. (2014). J. Chem. Phys..

[cit61] Thomas L. H. , Mathematical proceedings of the Cambridge philosophical society, 1927, pp. 542–548

[cit62] Fermi E. (1927). Rend. Accad. Naz. Lincei.

[cit63] Dirac P. A. , Mathematical proceedings of the Cambridge philosophical society, 1930, pp. 376–385

[cit64] Yang W. (1986). Phys. Rev. A.

[cit65] Hohenberg P., Kohn W. (1964). Phys. Rev..

[cit66] Perdew J. P., Burke K., Ernzerhof M. (1996). Phys. Rev. Lett..

[cit67] Perdew J. P., Ruzsinszky A., Csonka G. I., Vydrov O. A., Scuseria G. E., Constantin L. A., Zhou X., Burke K. (2008). Phys. Rev. Lett..

[cit68] Van Noorden R., Maher B., Nuzzo R. (2014). Nature.

[cit69] Kubler J., Hock K. H., Sticht J., Williams A. R. (1988). J. Phys. F: Met. Phys..

[cit70] Cuadrado R., Cerdá J. I. (2012). J. Phys.: Condens. Matter.

[cit71] Gómez-Ortiz F., Carral-Sainz N., Sifuna J., Monteseguro V., Cuadrado R., García-Fernández P., Junquera J. (2023). Comput. Phys. Commun..

[cit72] Fu C. L., Ho K. M. (1983). Phys. Rev. B: Condens. Matter Mater. Phys..

[cit73] Methfessel M., Paxton A. T. (1989). Phys. Rev. B: Condens. Matter Mater. Phys..

[cit74] Marzari N., Vanderbilt D., De Vita A., Payne M. C. (1999). Phys. Rev. Lett..

[cit75] Grimme S. (2006). J. Comput. Chem..

[cit76] Tkatchenko A., Scheffler M. (2009). Phys. Rev. Lett..

[cit77] Rydberg H., Dion M., Jacobson N., Schröder E., Hyldgaard P., Simak S. I., Langreth D. C., Lundqvist B. I. (2003). Phys. Rev. Lett..

[cit78] Nogueira F. , Castro A. and Marques M. A. L., in A Tutorial on Density Functional Theory, ed. C. Fiolhais, F. Nogueira and M. A. L. Marques, Springer Berlin Heidelberg, Berlin, Heidelberg, 2003, pp. 218–256

[cit79] Mori-Sánchez P., Cohen A. J., Yang W. (2006). J. Chem. Phys..

[cit80] Mori-Sánchez P., Cohen A. J., Yang W. (2008). Phys. Rev. Lett..

[cit81] Mori-Sánchez P., Cohen A. J., Yang W. (2009). Phys. Rev. Lett..

[cit82] Mori-Sánchez P., Cohen A. J. (2014). Phys. Chem. Chem. Phys..

[cit83] Anisimov V. I., Zaanen J., Andersen O. K. (1991). Phys. Rev. B: Condens. Matter Mater. Phys..

[cit84] Anisimov V. I., Solovyev I. V., Korotin M. A., Czyżyk M. T., Sawatzky G. A. (1993). Phys. Rev. B: Condens. Matter Mater. Phys..

[cit85] Solovyev I. V., Dederichs P. H., Anisimov V. I. (1994). Phys. Rev. B: Condens. Matter Mater. Phys..

[cit86] Himmetoglu B., Floris A., de Gironcoli S., Cococcioni M. (2014). Int. J. Quantum Chem..

[cit87] Campo V. L., Cococcioni M. (2010). J. Phys.: Condens. Matter.

[cit88] Perdew J. P., Parr R. G., Levy M., Balduz J. L. (1982). Phys. Rev. Lett..

[cit89] Towler M. D., Allan N. L., Harrison N. M., Saunders V. R., Mackrodt W. C., Aprà E. (1994). Phys. Rev. B: Condens. Matter Mater. Phys..

[cit90] Bengone O., Alouani M., Blöchl P., Hugel J. (2000). Phys. Rev. B: Condens. Matter Mater. Phys..

[cit91] Mazin I. I., Anisimov V. I. (1997). Phys. Rev. B: Condens. Matter Mater. Phys..

[cit92] Fang Z., Terakura K., Sawada H., Miyazaki T., Solovyev I. (1998). Phys. Rev. Lett..

[cit93] Fang Z., Solovyev I. V., Sawada H., Terakura K. (1999). Phys. Rev. B: Condens. Matter Mater. Phys..

[cit94] Liechtenstein A. I., Anisimov V. I., Zaanen J. (1995). Phys. Rev. B: Condens. Matter Mater. Phys..

[cit95] Hubbard J., Flowers B. H. (1963). Proc. R. Soc. London, Ser. A.

[cit96] Liechtenstein A. I., Anisimov V. I., Zaanen J. (1995). Phys. Rev. B: Condens. Matter Mater. Phys..

[cit97] Dudarev S. L., Botton G. A., Savrasov S. Y., Humphreys C. J., Sutton A. P. (1998). Phys. Rev. B: Condens. Matter Mater. Phys..

[cit98] Menescardi F., Ceresoli D. (2021). Appl. Sci..

[cit99] Rudenko A. N., Rösner M., Katsnelson M. I. (2023). npj Comput. Mater..

[cit100] Cococcioni M., de Gironcoli S. (2005). Phys. Rev. B: Condens. Matter Mater. Phys..

[cit101] Mosey N. J., Carter E. A. (2007). Phys. Rev. B: Condens. Matter Mater. Phys..

[cit102] Mosey N. J., Liao P., Carter E. A. (2008). J. Chem. Phys..

[cit103] Timrov I., Marzari N., Cococcioni M. (2018). Phys. Rev. B.

[cit104] Timrov I., Marzari N., Cococcioni M. (2021). Phys. Rev. B.

[cit105] Timrov I., Marzari N., Cococcioni M. (2022). Comput. Phys. Commun..

[cit106] Aryasetiawan F., Imada M., Georges A., Kotliar G., Biermann S., Lichtenstein A. I. (2004). Phys. Rev. B: Condens. Matter Mater. Phys..

[cit107] Aryasetiawan F., Karlsson K., Jepsen O., Schönberger U. (2006). Phys. Rev. B: Condens. Matter Mater. Phys..

[cit108] Amadon B., Applencourt T., Bruneval F. (2014). Phys. Rev. B: Condens. Matter Mater. Phys..

[cit109] Yu M., Yang S., Wu C., Marom N. (2020). npj Comput. Mater..

[cit110] Yu W., Zhang Z., Wan X., Guo H., Gui Q., Peng Y., Li Y., Fu W., Lu D., Ye Y., Guo Y. (2023). J. Chem. Theory Comput..

[cit111] Shahriari B., Swersky K., Wang Z., Adams R. P., de Freitas N. (2016). Proc. IEEE.

[cit112] Wang X., Jin Y., Schmitt S., Olhofer M. (2023). ACM Comput. Surv..

[cit113] Baroni S., de Gironcoli S., Dal Corso A., Giannozzi P. (2001). Rev. Mod. Phys..

[cit114] Giannozzi P., Baroni S., Bonini N., Calandra M., Car R., Cavazzoni C., Ceresoli D., Chiarotti G. L., Cococcioni M., Dabo I., Corso A. D., de Gironcoli S., Fabris S., Fratesi G., Gebauer R., Gerstmann U., Gougoussis C., Kokalj A., Lazzeri M., Martin-Samos L., Marzari N., Mauri F., Mazzarello R., Paolini S., Pasquarello A., Paulatto L., Sbraccia C., Scandolo S., Sclauzero G., Seitsonen A. P., Smogunov A., Umari P., Wentzcovitch R. M. (2009). J. Phys.: Condens. Matter.

[cit115] Cococcioni M., Marzari N. (2019). Phys. Rev. Mater..

[cit116] Timrov I., Aquilante F., Cococcioni M., Marzari N. (2022). PRX Energy.

[cit117] Naveas N., Pulido R., Marini C., Hernández-Montelongo J., Silván M. M. (2023). iScience.

[cit118] Giannozzi P., Andreussi O., Brumme T., Bunau O., Nardelli M. B., Calandra M., Car R., Cavazzoni C., Ceresoli D., Cococcioni M., Colonna N., Carnimeo I., Corso A. D., de Gironcoli S., Delugas P., Jr R. A. D., Ferretti A., Floris A., Fratesi G., Fugallo G., Gebauer R., Gerstmann U., Giustino F., Gorni T., Jia J., Kawamura M., Ko H.-Y., Kokalj A., Küçükbenli E., Lazzeri M., Marsili M., Marzari N., Mauri F., Nguyen N. L., Nguyen H.-V., de-la Roza A. O., Paulatto L., Poncé S., Rocca D., Sabatini R., Santra B., Schlipf M., Seitsonen A. P., Smogunov A., Timrov I., Thonhauser T., Umari P., Vast N., Wu X., Baroni S. (2017). J. Phys.: Condens. Matter.

[cit119] Giannozzi P., Baseggio O., Bonfà P., Brunato D., Car R., Carnimeo I., Cavazzoni C., de Gironcoli S., Delugas P., Ferrari Ruffino F., Ferretti A., Marzari N., Timrov I., Urru A., Baroni S. (2020). J. Chem. Phys..

[cit120] Pizzi G., Cepellotti A., Sabatini R., Marzari N., Kozinsky B. (2016). Comput. Mater. Sci..

[cit121] Huber S. P., Zoupanos S., Uhrin M., Talirz L., Kahle L., Häuselmann R., Gresch D., Müller T., Yakutovich A. V., Andersen C. W., Ramirez F. F., Adorf C. S., Gargiulo F., Kumbhar S., Passaro E., Johnston C., Merkys A., Cepellotti A., Mounet N., Marzari N., Kozinsky B., Pizzi G. (2020). Sci. Data.

[cit122] Uhrin M., Huber S. P., Yu J., Marzari N., Pizzi G. (2021). Comput. Mater. Sci..

[cit123] Macke E., Timrov I., Marzari N., Ciacchi L. C. (2024). J. Chem. Theory Comput..

[cit124] Esteras D. L., Baldoví J. J. (2023). Mater. Today Electron..

[cit125] Esteras D. L., Rybakov A., Ruiz A. M., Baldoví J. J. (2022). Nano Lett..

[cit126] Wang Y., Luo N., Zeng J., Tang L.-M., Chen K.-Q. (2023). Phys. Rev. B.

[cit127] Verma P., Truhlar D. G. (2016). Theor. Chem. Acc..

[cit128] Feng X.-B., Harrison N. M. (2004). Phys. Rev. B: Condens. Matter Mater. Phys..

[cit129] Moreira I. de P. R., Illas F., Martin R. L. (2002). Phys. Rev. B: Condens. Matter Mater. Phys..

[cit130] Grimme S., Hansen A., Brandenburg J. G., Bannwarth C. (2016). Chem. Rev..

[cit131] Kim M., Kim W. J., Lee E. K., Lebègue S., Kim H. (2016). Int. J. Quantum Chem..

[cit132] Kushchuk L. I., Veretimus D. K., Lega P. V., Antonenkova A. Y., Kartsev A. I. (2024). J. Surf. Invest.: X-Ray, Synchrotron Neutron Tech..

[cit133] Chittari B. L., Park Y., Lee D., Han M., MacDonald A. H., Hwang E., Jung J. (2016). Phys. Rev. B.

[cit134] Cuadrado R., Pruneda M., García A., Ordejón P. (2018). J. Phys.: Mater..

[cit135] Jiang X., Liu Q., Xing J., Liu N., Guo Y., Liu Z., Zhao J. (2021). Appl. Phys. Rev..

[cit136] Zheng F., Zhang P. (2021). Comput. Phys. Commun..

[cit137] Horton M. K., Montoya J. H., Liu M., Persson K. A. (2019). npj Comput. Mater..

[cit138] Sandratskii L. M. (1986). Phys. Status Solidi B.

[cit139] Sandratskii L. M. (1991). J. Phys.: Condens. Matter.

[cit140] Kurz P., Förster F., Nordström L., Bihlmayer G., Blügel S. (2004). Phys. Rev. B: Condens. Matter Mater. Phys..

[cit141] Bihlmayer G. , in Density-functional Theory of Magnetism, John Wiley & Sons, Ltd, 2007

[cit142] Olsen T. (2016). Phys. Rev. B.

[cit143] Sandratskii L. M. (2017). Phys. Rev. B.

[cit144] Zimmermann B., Bihlmayer G., Böttcher M., Bouhassoune M., Lounis S., Sinova J., Heinze S., Blügel S., Dupé B. (2019). Phys. Rev. B.

[cit145] Dupé M., Heinze S., Sinova J., Dupé B. (2018). Phys. Rev. B.

[cit146] Mortensen J. J., Hansen L. B., Jacobsen K. W. (2005). Phys. Rev. B: Condens. Matter Mater. Phys..

[cit147] Enkovaara J., Rostgaard C., Mortensen J. J., Chen J., Dułak M., Ferrighi L., Gavnholt J., Glinsvad C., Haikola V., Hansen H. A., Kristoffersen H. H., Kuisma M., Larsen A. H., Lehtovaara L., Ljungberg M., Lopez-Acevedo O., Moses P. G., Ojanen J., Olsen T., Petzold V., Romero N. A., Stausholm-Møller J., Strange M., Tritsaris G. A., Vanin M., Walter M., Hammer B., Häkkinen H., Madsen G. K. H., Nieminen R. M., Nørskov J. K., Puska M., Rantala T. T., Schiøtz J., Thygesen K. S., Jacobsen K. W. (2010). J. Phys.: Condens. Matter.

[cit148] Mortensen J. J., Larsen A. H., Kuisma M., Ivanov A. V., Taghizadeh A., Peterson A., Haldar A., Dohn A. O., Schäfer C., Jónsson E. Ö., Hermes E. D., Nilsson F. A., Kastlunger G., Levi G., Jónsson H., Häkkinen H., Fojt J., Kangsabanik J., Sødequist J., Lehtomäki J., Heske J., Enkovaara J., Winther K. T., Dulak M., Melander M. M., Ovesen M., Louhivuori M., Walter M., Gjerding M., Lopez-Acevedo O., Erhart P., Warmbier R., Würdemann R., Kaappa S., Latini S., Boland T. M., Bligaard T., Skovhus T., Susi T., Maxson T., Rossi T., Chen X., Schmerwitz Y. L. A., Schiøtz J., Olsen T., Jacobsen K. W., Thygesen K. S. (2024). J. Chem. Phys..

[cit149] Larsen A. H., Mortensen J. J., Blomqvist J., Castelli I. E., Christensen R., Dułak M., Friis J., Groves M. N., Hammer B., Hargus C., Hermes E. D., Jennings P. C., Jensen P. B., Kermode J., Kitchin J. R., Kolsbjerg E. L., Kubal J., Kaasbjerg K., Lysgaard S., Maronsson J. B., Maxson T., Olsen T., Pastewka L., Peterson A., Rostgaard C., Schiøtz J., Schütt O., Strange M., Thygesen K. S., Vegge T., Vilhelmsen L., Walter M., Zeng Z., Jacobsen K. W. (2017). J. Phys.: Condens. Matter.

[cit150] Kresse G., Hafner J. (1993). Phys. Rev. B: Condens. Matter Mater. Phys..

[cit151] Kresse G., Furthmüller J. (1996). Phys. Rev. B: Condens. Matter Mater. Phys..

[cit152] The FLEUR project, https://www.flapw.de/

[cit153] Wortmann D. , Michalicek G., Baadji N., Betzinger M., Bihlmayer G., Bröder J., Burnus T., Enkovaara J., Freimuth F., Friedrich C., Gerhorst C.-R., Granberg Cauchi S., Grytsiuk U., Hanke A., Hanke J.-P., Heide M., Heinze S., Hilgers R., Janssen H., Klüppelberg D. A., Kovacik R., Kurz P., Lezaic M., Madsen G. K. H., Mokrousov Y., Neukirchen A., Redies M., Rost S., Schlipf M., Schindlmayr A., Winkelmann M. and Blügel S., FLEUR, Zenodo, 2023, 10.5281/zenodo.7576163

[cit154] Ozaki T. (2003). Phys. Rev. B: Condens. Matter Mater. Phys..

[cit155] Ozaki T., Kino H. (2004). Phys. Rev. B: Condens. Matter Mater. Phys..

[cit156] Ozaki T., Kino H. (2005). Phys. Rev. B: Condens. Matter Mater. Phys..

[cit157] Prayitno T. B., Ishii F. (2018). J. Phys. Soc. Jpn..

[cit158] Prayitno T. B., Ishii F. (2019). J. Phys. Soc. Jpn..

[cit159] Zhu Y., Kong X., Rhone T. D., Guo H. (2018). Phys. Rev. Mater..

[cit160] Yang K., Wang G., Liu L., Lu D., Wu H. (2021). Phys. Rev. B.

[cit161] Ruiz A. M., Esteras D. L., Rybakov A., Baldoví J. J. (2022). Dalton Trans..

[cit162] Lee K., Dismukes A. H., Telford E. J., Wiscons R. A., Wang J., Xu X., Nuckolls C., Dean C. R., Roy X., Zhu X. (2021). Nano Lett..

[cit163] Lee K., Dismukes A. H., Telford E. J., Wiscons R. A., Wang J., Xu X., Nuckolls C., Dean C. R., Roy X., Zhu X. (2021). Nano Lett..

[cit164] Ruiz A. M., Esteras D. L., López-Alcalá D., Baldoví J. J. (2024). Nano Lett..

[cit165] Li X., Zhu M., Wang Y., Zheng F., Dong J., Zhou Y., You L., Zhang J. (2023). Appl. Phys. Lett..

[cit166] Ma P.-W., Dudarev S. L. (2015). Phys. Rev. B: Condens. Matter Mater. Phys..

[cit167] Grimme S. (2006). J. Comput. Chem..

[cit168] Grimme S., Antony J., Ehrlich S., Krieg H. (2010). J. Chem. Phys..

[cit169] Grimme S., Ehrlich S., Goerigk L. (2011). J. Comput. Chem..

[cit170] Caldeweyher E., Ehlert S., Hansen A., Neugebauer H., Spicher S., Bannwarth C., Grimme S. (2019). J. Chem. Phys..

[cit171] Hermann J., Stöhr M., Góger S., Chaudhuri S., Aradi B., Maurer R. J., Tkatchenko A. (2023). J. Chem. Phys..

[cit172] Tkatchenko A., Scheffler M. (2009). Phys. Rev. Lett..

[cit173] Bučko T., Lebègue S., Hafner J., Ángyán J. G. (2013). J. Chem. Theory Comput..

[cit174] Bučko T., Lebègue S., Ángyán J. G., Hafner J. (2014). J. Chem. Phys..

[cit175] Tkatchenko A., DiStasio R. A., Car R., Scheffler M. (2012). Phys. Rev. Lett..

[cit176] Ambrosetti A., Reilly A. M., DiStasio J., Robert A., Tkatchenko A. (2014). J. Chem. Phys..

[cit177] Gould T., Bučko T. (2016). J. Chem. Theory Comput..

[cit178] Gould T., Lebègue S., Ángyán J. G., Bučko T. (2016). J. Chem. Theory Comput..

[cit179] Soler J. M., Artacho E., Gale J. D., Garcia A., Junquera J., Ordejon P., Sanchez-Portal D. (2002). J. Phys.: Condens. Matter.

[cit180] Dion M., Rydberg H., Schröder E., Langreth D. C., Lundqvist B. I. (2004). Phys. Rev. Lett..

[cit181] Lee K., Murray E. D., Kong L., Lundqvist B. I., Langreth D. C. (2010). Phys. Rev. B: Condens. Matter Mater. Phys..

[cit182] Klimeš J., Bowler D. R., Michaelides A. (2009). J. Phys.: Condens. Matter.

[cit183] Cooper V. R. (2010). Phys. Rev. B: Condens. Matter Mater. Phys..

[cit184] Berland K., Hyldgaard P. (2014). Phys. Rev. B: Condens. Matter Mater. Phys..

[cit185] Vydrov O. A., Van Voorhis T. (2010). J. Chem. Phys..

[cit186] Heide M., Bihlmayer G., Blügel S. (2009). Phys. B.

[cit187] Shick A. B., Liechtenstein A. I., Pickett W. E. (1999). Phys. Rev. B: Condens. Matter Mater. Phys..

[cit188] Barone V., Casarin M., Forrer D., Pavone M., Sambi M., Vittadini A. (2009). J. Comput. Chem..

[cit189] Becke A. D., Johnson E. R. (2007). J. Chem. Phys..

[cit190] Otero-de-la Roza A., Johnson E. R. (2012). J. Chem. Phys..

[cit191] Han M. J., Ozaki T., Yu J. (2006). Phys. Rev. B: Condens. Matter Mater. Phys..

[cit192] Ryee S., Han M. J. (2018). J. Phys.: Condens. Matter.

[cit193] Dion M., Rydberg H., Schröder E., Langreth D. C., Lundqvist B. I. (2005). Phys. Rev. Lett..

[cit194] Gonze X., Amadon B., Antonius G., Arnardi F., Baguet L., Beuken J.-M., Bieder J., Bottin F., Bouchet J., Bousquet E., Brouwer N., Bruneval F., Brunin G., Cavignac T., Charraud J.-B., Chen W., Côté M., Cottenier S., Denier J., Geneste G., Ghosez P., Giantomassi M., Gillet Y., Gingras O., Hamann D. R., Hautier G., He X., Helbig N., Holzwarth N., Jia Y., Jollet F., Lafargue-Dit-Hauret W., Lejaeghere K., Marques M. A. L., Martin A., Martins C., Miranda H. P. C., Naccarato F., Persson K., Petretto G., Planes V., Pouillon Y., Prokhorenko S., Ricci F., Rignanese G.-M., Romero A. H., Schmitt M. M., Torrent M., van Setten M. J., Troeye B. V., Verstraete M. J., Zérah G., Zwanziger J. W. (2020). Comput. Phys. Commun..

[cit195] Gonze X., Seddon B., Elliott J. A., Tantardini C., Shapeev A. V. (2022). J. Chem. Theory Comput..

[cit196] Van Troeye B., Torrent M., Gonze X. (2016). Phys. Rev. B.

[cit197] Amadon B., Jollet F., Torrent M. (2008). Phys. Rev. B: Condens. Matter Mater. Phys..

[cit198] Clark S. J., Segall M. D., Pickard C. J., Hasnip P. J., Probert M. I. J., Refson K., Payne M. C. (2005). Z. Kristallogr. - Cryst. Mater..

[cit199] Ortmann F., Bechstedt F., Schmidt W. G. (2006). Phys. Rev. B: Condens. Matter Mater. Phys..

[cit200] Becke A. D., Johnson E. R. (2007). J. Chem. Phys..

[cit201] E. D. Team, ELK: Embedded Learning Library, 2024, https://elk.sourceforge.io/, accessed: 2024-10-29

[cit202] Blaha P., Schwarz K., Tran F., Laskowski R., Madsen G. K. H., Marks L. D. (2020). J. Chem. Phys..

[cit203] Peng H., Yang Z.-H., Perdew J. P., Sun J. (2016). Phys. Rev. X.

[cit204] Lu X., Fei R., Yang L. (2019). Phys. Rev. B.

[cit205] Wahab D. A., Augustin M., Valero S. M., Kuang W., Jenkins S., Coronado E., Grigorieva I. V., Vera-Marun I. J., Navarro-Moratalla E., Evans F. L., Novoselov K. S., Santos E. J. G. (2020). Adv. Mater..

[cit206] Olsen T. (2017). Phys. Rev. B.

[cit207] Ködderitzsch D., Hergert W., Temmerman W. M., Szotek Z., Ernst A., Winter H. (2002). Phys. Rev. B: Condens. Matter Mater. Phys..

[cit208] Torelli D., Olsen T. (2020). J. Phys.: Condens. Matter.

[cit209] Xu B., Meyer S., Verstraete M. J., Bellaiche L., Dupé B. (2021). Phys. Rev. B.

[cit210] Solovyev I. V. (2024). J. Phys.: Condens. Matter.

[cit211] Szilva A., Kvashnin Y., Stepanov E. A., Nordström L., Eriksson O., Lichtenstein A. I., Katsnelson M. I. (2023). Rev. Mod. Phys..

[cit212] Katsnelson M. I., Lichtenstein A. I. (2000). Phys. Rev. B: Condens. Matter Mater. Phys..

[cit213] Mazurenko V. V., Anisimov V. I. (2005). Phys. Rev. B: Condens. Matter Mater. Phys..

[cit214] He X., Helbig N., Verstraete M. J., Bousquet E. (2021). Comput. Phys. Commun..

[cit215] Korotin D. M., Mazurenko V. V., Anisimov V. I., Streltsov S. V. (2015). Phys. Rev. B: Condens. Matter Mater. Phys..

[cit216] Durhuus F. L., Skovhus T., Olsen T. (2023). J. Phys.: Condens. Matter.

[cit217] Antropov V., Katsnelson M., Liechtenstein A. (1997). Phys. B.

[cit218] Katsnelson M. I., Kvashnin Y. O., Mazurenko V. V., Lichtenstein A. I. (2010). Phys. Rev. B: Condens. Matter Mater. Phys..

[cit219] Szilva A., Costa M., Bergman A., Szunyogh L., Nordström L., Eriksson O. (2013). Phys. Rev. Lett..

[cit220] Pizzi G., Vitale V., Arita R., Blügel S., Freimuth F., Géranton G., Gibertini M., Gresch D., Johnson C., Koretsune T., Ibañez-Azpiroz J., Lee H., Lihm J.-M., Marchand D., Marrazzo A., Mokrousov Y., Mustafa J. I., Nohara Y., Nomura Y., Paulatto L., Poncé S., Ponweiser T., Qiao J., Thöle F., Tsirkin S. S., Wierzbowska M., Marzari N., Vanderbilt D., Souza I., Mostofi A. A., Yates J. R. (2020). J. Phys.: Condens. Matter.

[cit221] Yoon H., Kim T. J., Sim J.-H., Han M. J. (2020). Comput. Phys. Commun..

[cit222] Oroszlány L., Ferrer J., Deák A., Udvardi L., Szunyogh L. (2019). Phys. Rev. B.

[cit223] Martínez-Carracedo G., Oroszlány L., García-Fuente A., Nyári B., Udvardi L., Szunyogh L., Ferrer J. (2023). Phys. Rev. B.

[cit224] Lu X., Fei R., Yang L. (2019). Phys. Rev. B.

[cit225] Majlis N. , The Quantum Theory of Magnetism, World Scientific, 2nd edn, 2007

[cit226] Pajda M., Kudrnovský J., Turek I., Drchal V., Bruno P. (2001). Phys. Rev. B: Condens. Matter Mater. Phys..

[cit227] Malarz K. , Zborek M. and Wrobel B., Curie temperature for an Ising model on Archimedean lattices, 2005

[cit228] Codello A. (2010). J. Phys. A: Math. Theor..

[cit229] Vanherck J., Bacaksiz C., Sorée B., Miloševič M. V., Magnus W. (2020). Appl. Phys. Lett..

[cit230] Zhu Y., Kong X., Rhone T. D., Guo H. (2018). Phys. Rev. Mater..

[cit231] Holstein T., Primakoff H. (1940). Phys. Rev..

[cit232] Rajeev Pavizhakumari V., Skovhus T., Olsen T. (2025). J. Phys.:Condens. Matter.

[cit233] Dyson F. J. (1956). Phys. Rev..

[cit234] Maleev S. V. (1958). Sov. Phys. JETP.

[cit235] Stanek D., Sushkov O. P., Uhrig G. S. (2011). Phys. Rev. B: Condens. Matter Mater. Phys..

[cit236] Jordan C. , Calculus of Finite Differences, American Elsevier Publishing Company, Inc., New York, 2nd edn, 1965

[cit237] König J., Hucht A. (2021). SciPost Phys..

[cit238] Vogl M., Laurell P., Zhang H., Okamoto S., Fiete G. A. (2020). Phys. Rev. Res..

[cit239] Mkhitaryan V. V., Ke L. (2021). Phys. Rev. B.

[cit240] Lima W. P., Araújo F. R. V., da Costa D. R., Sena S. H. R., Pereira J. M. (2022). Braz. J. Phys..

[cit241] Olsen T. (2019). MRS Commun..

[cit242] Krauth W. , Statistical Mechanics Algorithms and Computations, Oxford University Press, 1st edn, 2006

[cit243] Hinzke D., Nowak U. (1999). Comput. Phys. Commun..

[cit244] Liu J., Sun Q., Kawazoe Y., Jena P. (2016). Phys. Chem. Chem. Phys..

[cit245] Kim H. H., Yang B., Li S., Jiang S., Jin C., Tao Z., Nichols G., Sfigakis F., Zhong S., Li C., Tian S., Cory D. G., Miao G.-X., Shan J., Mak K. F., Lei H., Sun K., Zhao L., Tsen A. W. (2019). Proc. Natl. Acad. Sci. U. S. A..

[cit246] Zhang Z., Shang J., Jiang C., Rasmita A., Gao W., Yu T. (2019). Nano Lett..

[cit247] Alzate-Cardona J. D., Sabogal-Suárez D., Evans R. F. L., Restrepo-Parra E. (2019). J. Phys.: Condens. Matter.

[cit248] Zhang Y., Wang B., Guo Y., Li Q., Wang J. (2021). Comput. Mater. Sci..

[cit249] Evans R. F. L., Atxitia U., Chantrell R. W. (2015). Phys. Rev. B: Condens. Matter Mater. Phys..

[cit250] Jenkins S., Rózsa L., Atxitia U., Evans R. F. L., Novoselov K. S., Santos E. J. G. (2022). Nat. Commun..

[cit251] Asselin P., Evans R. F. L., Barker J., Chantrell R. W., Yanes R., Chubykalo-Fesenko O., Hinzke D., Nowak U. (2010). Phys. Rev. B: Condens. Matter Mater. Phys..

[cit252] VAMPIRE software package, version 6, https://vampire.york.ac.uk/

[cit253] Müller G. P., Hoffmann M., Dißelkamp C., Schürhoff D., Mavros S., Sallermann M., Kiselev N. S., Jónsson H., Blügel S. (2019). Phys. Rev. B.

[cit254] Rüßmann P., Ribas Sobreviela J., Sallermann M., Hoffmann M., Rhiem F., Blügel S. (2022). Front. Mater..

[cit255] Bisotti M.-A., Cortés-Ortuño D., Pepper R., Wang W., Beg M., Kluyver T., Fangohr H. (2018). J. Open Res. Softw..

[cit256] Skubic B., Hellsvik J., Nordström L., Eriksson O. (2008). J. Phys.: Condens. Matter.

[cit257] Coffey W. T. and Kalmykov Y. P., The Langevin Equation, World Scientific, 3rd edn, 2012

[cit258] Callen H. B. (1963). Phys. Rev..

[cit259] Vanherck J., Sorée B., Magnus W. (2018). J. Phys.: Condens. Matter.

[cit260] Pelissetto A., Vicari E. (2002). Phys. Rep..

[cit261] Salinas S. R. A. , Introduction to statistical physics, Springer New York, New York, 2001

[cit262] Bander M., Mills D. L. (1988). Phys. Rev. B.

[cit263] Wang X., Li D., Li Z., Wu C., Che C.-M., Chen G., Cui X. (2021). ACS Nano.

[cit264] Fuh H.-R., Chang C.-R., Wang Y.-K., Evans R. F. L., Chantrell R. W., Jeng H.-T. (2016). Sci. Rep..

[cit265] Sheng H., Long H., Zou G., Bai D., Zhang J., Wang J. (2021). J. Mater. Sci..

[cit266] Abdollahi M., Tagani M. B. (2023). Phys. Rev. B.

[cit267] Guo Y., Zhang Y., Yuan S., Wang B., Wang J. (2018). Nanoscale.

[cit268] Wang H., Qi J., Qian X. (2020). Appl. Phys. Lett..

[cit269] Kim H. H., Yang B., Li S., Jiang S., Jin C., Tao Z., Nichols G., Sfigakis F., Zhong S., Li C., Tian S., Cory D. G., Miao G.-X., Shan J., Mak K. F., Lei H., Sun K., Zhao L., Tsen A. W. (2019). Proc. Natl. Acad. Sci. U. S. A..

[cit270] Zhang Z., Shang J., Jiang C., Rasmita A., Gao W., Yu T. (2019). Nano Lett..

[cit271] Chen W., Zhang J. M., Nie Y. Z., Xia Q. L., Guo G. H. (2020). J. Magn. Magn. Mater..

[cit272] Bedoya-Pinto A., Ji J.-R., Pandeya A. K., Gargiani P., Valvidares M., Sessi P., Taylor J. M., Radu F., Chang K., Parkin S. S. P. (2021). Science.

[cit273] Wang M., Lei B., Zhu K., Deng Y., Tian M., Xiang Z., Wu T., Chen X. (2024). npj 2D Mater. Appl..

[cit274] Zhang G., Guo F., Wu H., Wen X., Yang L., Jin W., Zhang W., Chang H. (2022). Nat. Commun..

[cit275] Shen Z.-X., Bo X., Cao K., Wan X., He L. (2021). Phys. Rev. B.

[cit276] Chen X., Lin Z.-Z., Cheng L.-R. (2021). Chin. Phys. B.

